# Green‐Light Activatable BODIPY and Coumarin 5’‐Caps for Oligonucleotide Photocaging

**DOI:** 10.1002/chem.202200477

**Published:** 2022-05-17

**Authors:** Janik Kaufmann, Patricia Müller, Eleni Andreadou, Alexander Heckel

**Affiliations:** ^1^ Institute for Organic Chemistry and Chemical Biology Goethe University Frankfurt Max-von-Laue-Str. 7 60438 Frankfurt Germany

**Keywords:** photochemistry, oligonucleotides, photolabile protecting groups, green gap, photo-tethering, click chemistry, bioconjugation

## Abstract

We synthesized two green‐light activatable 5’‐caps for oligonucleotides based on the BODIPY and coumarin scaffold. Both bear an alkyne functionality allowing their use in numerous biological applications. They were successfully incorporated in oligonucleotides via solid‐phase synthesis. Copper‐catalyzed alkyne‐azide cycloaddition (CuAAC) using a bisazide photo‐tether gave cyclic oligonucleotides that could be relinearized by activation with green light and were shown to exhibit high stability against exonucleases. Chemical ligation as another example for bioconjugation yielded oligonucleotides with an internal strand break site. Irradiation at 530 nm or 565 nm resulted in complete photolysis of both caging groups.

## Graphical abstract

**Green‐light absorbing BODIPY** and coumarin derivatives can be used as phosphoramidites in oligonucleotide synthesis. CuAAC reactions allow cyclization and therefore fast photocaging or the introduction of other photolabile modification at those 5’‐caps. Both caps are readily photocleaved within the green gap (530 and 565 nm), hence they avoid UV damage and offer deeper tissue penetration, especially in plants.

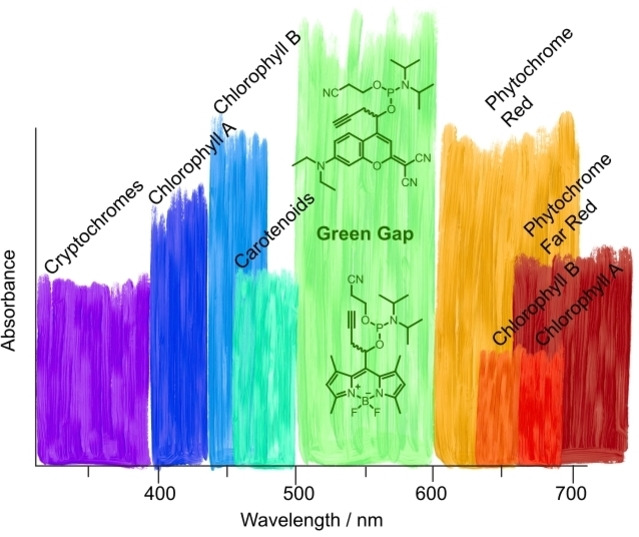

## Introduction

Within the last decades, photocaging has become an important tool to control biological systems. The extensive research on photocages[^1^, ^2^] led to several different strategies and methods[^3^, ^4^] allowing numerous applications from structural investigation^[5]^ to RNA localization^[6]^ up to the regulation of biological processes when attached to proteins^[7]^ or oligonucleotides.[^8^, ^9^] Photocages offer spatio‐temporal control over their targets and are excellent candidates for in vivo use due to their non‐invasive cleavage with light. Light, in contrast to chemical reagents, is an ideal orthogonal trigger signal that does not cause damage to biological systems as long as its wavelength lies beyond the UV region of the electromagnetic spectrum. UV light can cause cell damage^[10]^ and in addition, it has a low penetration depth in biological tissue.^[11]^ Therefore, the need for photocages that can be cleaved above 400 nm keeps growing. One important window along the way to the red region of the visible spectrum is the green gap in plants (500–600 nm) whose chromophore systems and light‐harvesting complexes show absorbance in most other parts of the visible range.^[12]^

BODIPYs are a prominent class of molecules with absorption maxima situated within the green gap. Widely used as fluorophore markers,[^13^, ^14^, ^15^, ^16^, ^17^] laser dyes,[^18^, ^19^] in OLEDs,[^20^, ^21^] or phototherapy,^[22]^ their uncaging properties have been discovered by the groups of Winter,^[23]^ Weinstain^[24]^ and Klán^[25]^ several years ago. Klán was even able to show release of CO in vivo with white light.^[25]^ The effects of different modifications at the BODIPY core have been demonstrated in systematic studies of said groups and helped overcome the poor photorelease quantum yield unsubstituted BODIPYs typically have.^[26]^ Together with their high extinction coefficients and the possibility to shift their absorption maxima up to the red region of the visible spectrum,^[27]^ BODIPY cages have gained more and more attention.

However, the use of BODIPYs in an oligonucleotide context was mostly restricted to fluorescence tracking so far. Solid‐phase synthesis was successful with BODIPY fluorophores being covalently bound to nucleobases^[28]^ or used as a 5’‐end labeling phosphoramidite where long alkyl chains linked the BODIPY moiety to the phosphate group and therefore blocked the caging site.[^29^, ^30^]

Another interesting and well‐known class of photocages are 7‐diethylaminocoumarins. Usually uncaged with wavelengths around 400 nm,^[31]^ they can be modified to shift their absorption maxima to higher wavelengths (up to 680 nm).^[32]^ Commonly used strategies to achieve shifts in absorption maxima are extension of the π‐system in 3‐position[^33^, ^34^] or substitution in 2‐position.^[35]^ As the group of Schnermann recently demonstrated, substitution of the lactone oxygen atom with difluoro moieties resulted in additional bathochromic shifts enabling the synthesis of coumarin fluorophores with absorption maxima even in the red region of visible light.^[32]^

Coumarin cages have been used extensively in oligonucleotide caging. Our group presented the synthesis of a strand break coumarin phosphoramidite,^[36]^ as well as alkyne‐modified coumarin phosphoramidites for direct incorporation in solid‐phase synthesis.[^37^, ^38^] The latter ones enabled cyclization or so‐called “photo‐tethering” with bisazide linkers under copper catalysis. Temporary cyclization with a photocleavable linker is a formidable way to prevent an oligonucleotide from forming an active conformation or especially also from hybridization to a counter strand. With fewer cages needed compared to local nucleobase caging, minimized synthetic effort and shorter irradiation times, photo‐tethering is a suitable strategy to even control the function of long oligonucleotides. Deiters et al. gave another example of oligonucleotide cyclization when modulating morpholinos in zebrafish, introducing a green‐light activatable dicyanocoumarin linker post‐synthetically.^[39]^ An additional advantage of cyclic oligonucleotides is the higher stability they exhibit in cell media due to the absence of targets for exonucleases.[^40^, ^41^]

In this publication, we report the transformation of alkyne‐bearing *meso*‐substituted BODIPY and dicyanocoumarin derivatives to phosphoramidites that can be used for 5’‐capping of oligonucleotides. They showed sufficient stability for solid‐phase synthesis and allowed photo‐tethering with minimal synthetic effort. Photolysis was shown to be successful under physiological conditions with green light lying perfectly within the green gap of plants.

## Results and Discussion

**Scheme 1:**
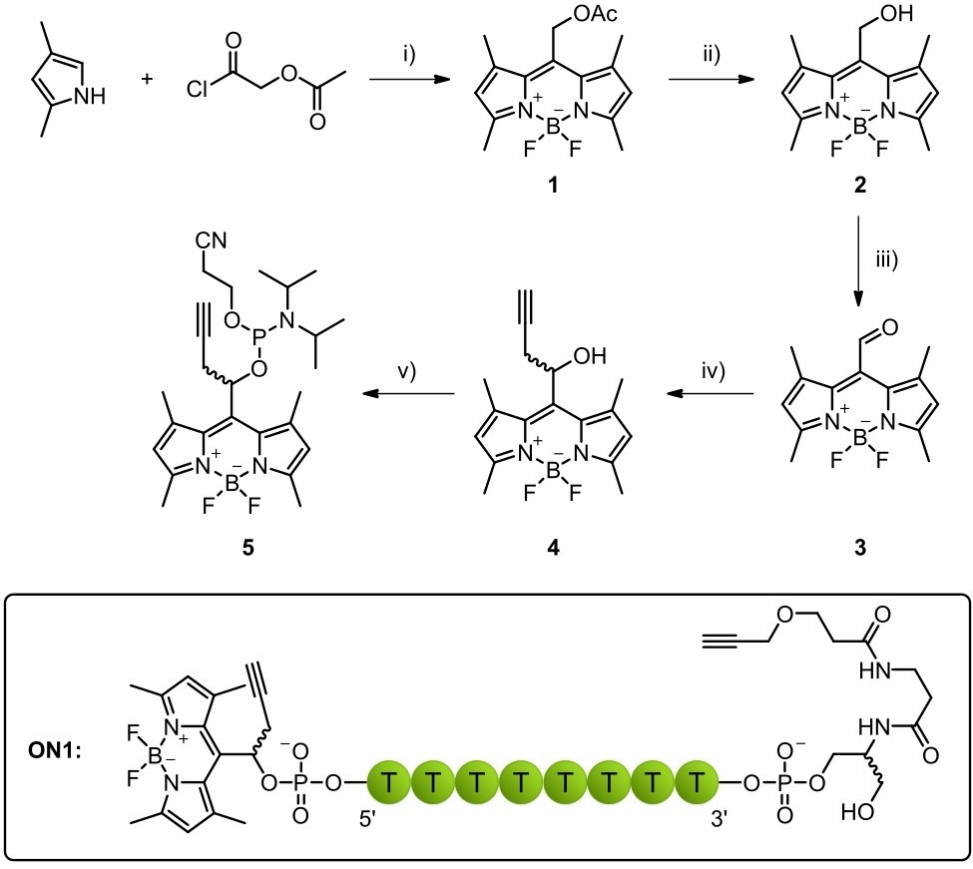
Synthesis of BODIPY phosphoramidite **5**. i) 1. DCM, reflux, 2 h; 2. DIPEA, BF_3_⋅OEt_2_, rt, 30 min, 37 %. ii) NaOH, MeOH/H_2_O, DCM, rt, 5 h, 76 %. iii) DCM, DMP, 0 °C – rt, 2 h, 55 %. iv) DMF, propargyl bromide, Zn*, 0 °C, 30 min, quant. v) DCM, DIPEA, PN(*i*Pr)_2_OCE−Cl, rt, 3 h, 87 %.

Synthesis of BODIPY phosphoramidite **5** started from 2,4‐dimethylpyrrole and acetoxyacetyl chloride following traditional reaction pathways leading to aldehyde **3** (Scheme 1).^[42]^ A propargyl group was introduced in the next step via a Barbier reaction to afford the main alcohol **4**.

With 53 500 M^−1^ cm^−1^ the extinction coefficient of BODIPY alcohol **4** in MeOH was found to lie perfectly within the typical range of similar BODIPY derivatives (Table 1, Figure S3a in Supporting Information).^[26]^ Addition of water even led to a slight increase in the extinction coefficient.

**Table 1:** Photochemical data of the synthesized molecules **4** and **13** and the respective oligonucleotides **ON1** and **ON3**.

compound	λ_abs, max_ [nm]	λ_em, max_ [nm]	ϵ_max_ [M^−1^ cm^−1^]	10^2^⋅Φ_u_	Φ_u_⋅ϵ_max_ [M^−1^ cm^−1^]
**4**	510^[a]^	559^[a]^	53 500^[a]^	–	–
	512^[b]^	562^[b]^	55 100^[b]^	–	–
**13**	484^[a]^	550^[a]^	30 100^[a]^	–	–
	492^[b]^	557^[b]^	35 100^[b]^	–	–
**ON1**	518^[c]^	532^[c]^	–	0.012^[c]^	6.9
**ON3**	502^[c]^	563^[c]^	–	0.26^[c]^	91

[a] in MeOH, [b] in MeOH/1x PBS (1 : 1), [c] in 1x PBS.

Incubation of BODIPY alcohol **4** with commonly used solid‐phase reagents gave a good first insight into the stability of the BODIPY core against the different conditions in solid‐phase synthesis. To that end, a small amount of compound **4** was dissolved in dichloromethane in separate reaction tubes and the solid‐phase reagents were added subsequently. After shaking for 15 min at room temperature, no degradation could be seen when compared to the starting material by TLC (Figure S1a).

**Figure 1:**
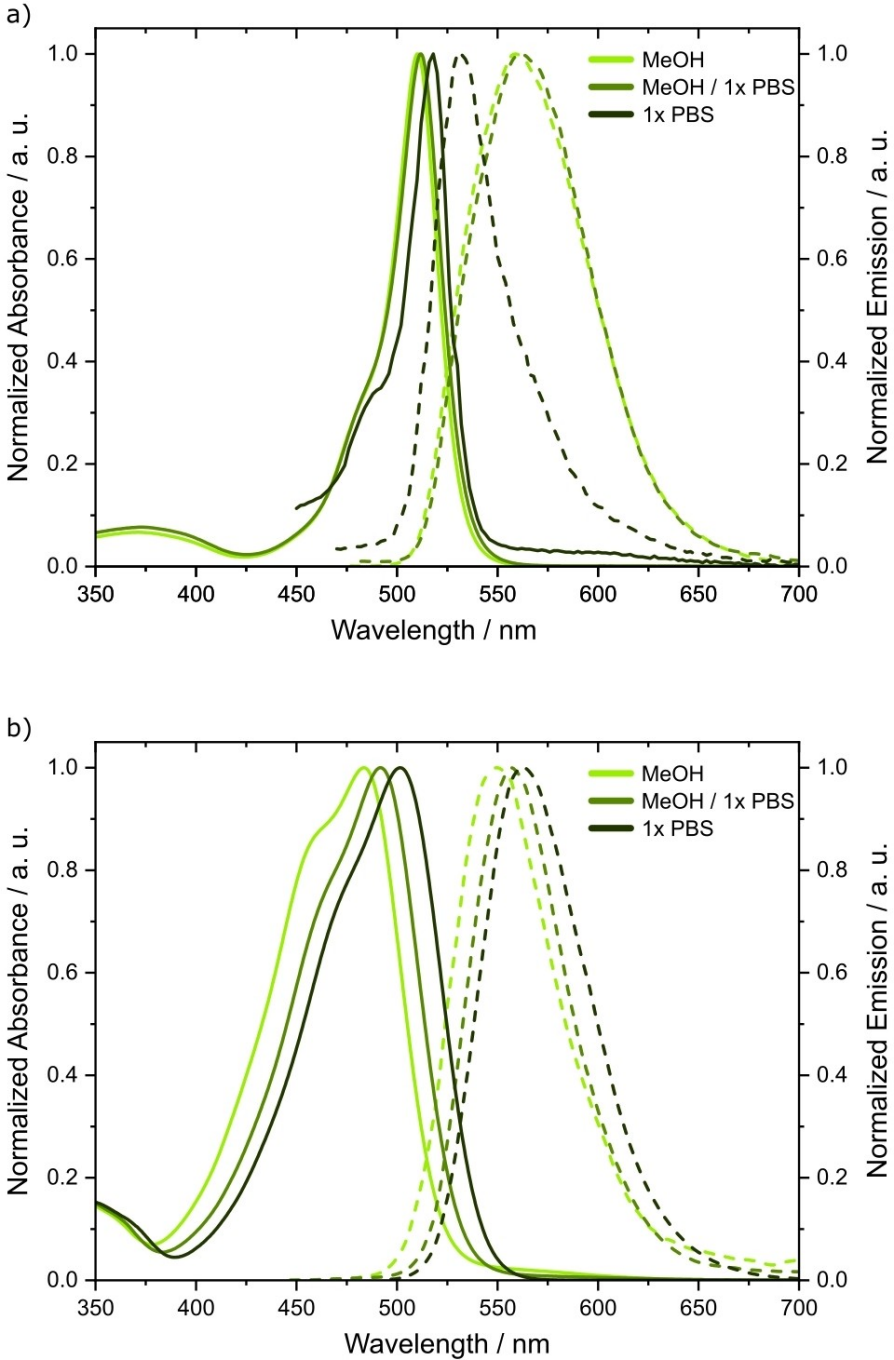
Absorbance (solid lines) and emission spectra (dashed lines) of small molecules in MeOH and MeOH/1x PBS (1 : 1) and oligonucleotides in 1x PBS. a) Measured spectra for BODIPY alcohol **4** and BODIPY 5’‐capped **ON1**. b) Measured spectra for coumarin alcohol **13** and coumarin 5’‐capped **ON3**.

Motivated by these first results, BODIPY alcohol **4** was converted to phosphoramidite **5**, which was then used to synthesize oligonucleotide sequence **ON1** with a BODIPY 5’‐cap. A commercially available serinol alkyne modifier was incorporated at the 3’‐end (Scheme 1) to allow cyclization reactions in later stages. The coupling time of the BODIPY phosphoramidite **5** was extended to 12 min. In contrast to other publications where BODIPY was used as a fluorophore,[^28^, ^30^] cleavage from the solid support in this case was not successful when carried out with 30 % NH_3_ solution. Since phosphate served as a good leaving group for the BODIPY cage, complete hydrolysis occurred within the first hour of incubation, leaving behind a 5’‐phosphate modified oligonucleotide (data not shown). This problem could be circumvented by cleavage with 0.05 m K_2_CO_3_ in MeOH. Under these conditions, only minor amounts of hydrolyzed side products were found, and with the HPLC gradient used, two signals showing a 520 nm absorbance band were visible. Both signals shared the same mass, suggesting that they are diastereomers of **ON1**. Compared to the absorption and fluorescence spectra measured with BODIPY alcohol **4** in MeOH, the absorption maximum of **ON1** in 1x PBS was shifted to higher wavelengths (Figure 1a, Table 1).

First irradiation experiments showed that BODIPY in **ON1** can be cleaved in 1x PBS using a 530 nm or even 565 nm LED (Figure S6a). The quantum yield for the release of the BODIPY cage from **ON1** was determined to be 0.012 % in 1x PBS (Table 1, Figure S4a, Figure S5a) using our recently published fulgide actinometry procedure,^[43]^ a value which is not untypical for this class of cages in aqueous media.^[44]^

**Scheme 2:**
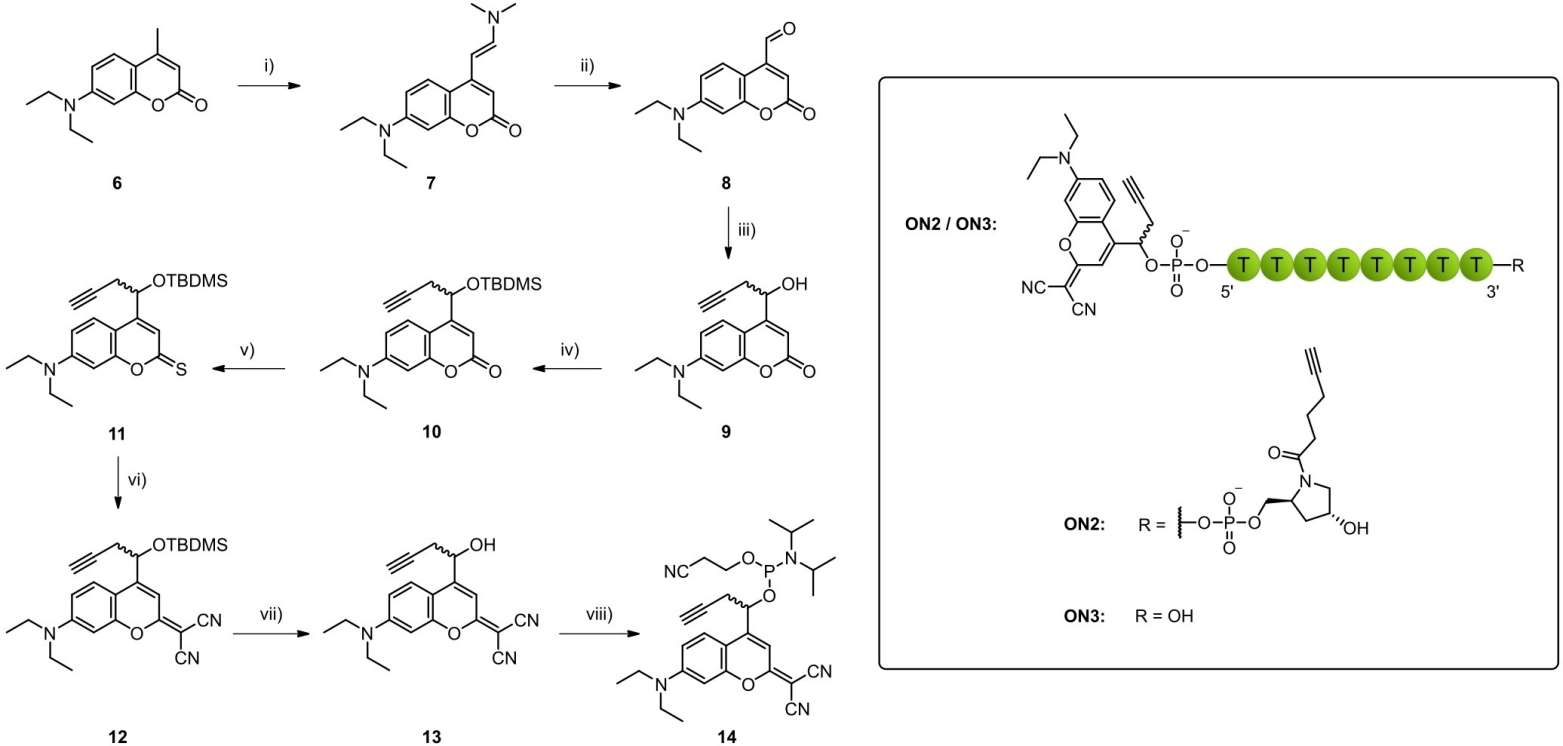
Synthesis of coumarin phosphoramidite **14**. i) DMF, DMF‐DMA, reflux, 8 h, 94 % (crude). ii) THF/H_2_O, NaIO_4_, rt, 4 h, quant. (crude). iii) DMF, propargyl bromide, Zn*, 0 °C, 30 min, 58 %. iv) DMF, TBDMS−Cl, imidazole, rt, 72 h, 75 %. v) toluene, Lawesson's reagent, reflux – rt, 70 h, 74 %. vi) toluene, DMAP, PbO, malononitrile, 90 °C, 24 h, 49 %. vii) THF, AcOH, TBAF, rt, 72 h, 90 %. viii) DCM, DIPEA, PN(*i*Pr)_2_OCE−Cl, rt, 19 h, 80 %.

Motivated by these good results obtained with BODIPY, we turned our view to another cage that can be activated with green light. Dicyanomethylene substitution is a known modification for coumarins shifting their absorption maxima to higher wavelengths. Compared to unsubstituted BODIPYs, dicyanocoumarins exhibit lower extinction coefficients around 33 000 but their uncaging quantum yields are usually much higher.^[35]^ Also, coumarin‐caged phosphate groups have already been shown to be compatible with solid‐phase synthesis as well as cleavage and deprotection conditions making them another promising class of molecules for oligonucleotide caging within the green gap.[^36^, ^37^] The dicyanosubstituted coumarin derivative introduced by Jullien et al. was chosen as a starting point for our second green‐light activatable 5’‐cap (Scheme 2).^[35]^ The original synthetic pathway was varied starting with 7‐diethylamino‐4‐methylcoumarin **6** and following the procedure published by Göbel et al.^[45]^ to obtain coumarin aldehyde **8**. At this point, the propargyl residue could be introduced again using propargyl bromide and zinc. The resulting hydroxy group in coumarin alcohol **9** was TBDMS‐protected before introduction of the dicyano moiety within two steps. Dicyanocoumarin **12** was then deprotected affording alcohol **13** that could be further converted to phosphoramidite **14**.

As expected, the extinction coefficient of compound **13** was lower than for BODIPY **4** but with around 30 000 M^−1^ cm^−1^ still high (Table 1, Figure S3b). Also, a larger bathochromic shift to the green gap than in the case of BODIPY **4** was observed when increasing the water ratio in the solvent mixture (Table 1, Figure 1b). Coumarin **13** showed good stability against solid‐phase reagents as well (Figure S1b) so that compound **14** was then used for oligonucleotide synthesis.

A cyclizable oligonucleotide **ON2** was synthesized on a hydroxyprolinol alkyne solid support along with a second oligonucleotide **ON3** that lacked the 3’‐modification (Scheme 2). While BODIPY showed hydrolysis upon the basic conditions in 30 % NH_3_ solution, coumarin phosphate cages seemed more stable under the same conditions (data not shown). Still, partly hydrolysis was observable so that cleavage and deprotection was also carried out in 0.05 M K_2_CO_3_ solution. Again, a bathochromic shift in the absorption maximum of the cage was observed when measured in 1x PBS, reaching the green gap with 502 nm (Table 1, Figure 1b). Also, similar to **ON1**, **ON2** and **ON3** showed two peaks in the HPLC analysis which could be attributed to diastereomers (Figure 3b, Figure S5b).

As in the case of BODIPY, first photolysis attempts of **ON2** and **ON3** were successful using a 530 nm or 565 nm LED (Figure S6b). Compared to **ON1**, photolysis of the coumarin cage in **ON2** and **ON3** was much faster. Hence, we performed a direct comparison of BODIPY‐caged **ON1** and coumarin‐caged **ON2**. The setup consisted of a 530 nm LED focused on a cover glass. Three small wells with a volume of 13 μL were glued to the cover glass so that they were all placed in the focal area of the light source and could be irradiated at the same time (Figure S7).

**Figure 2:**
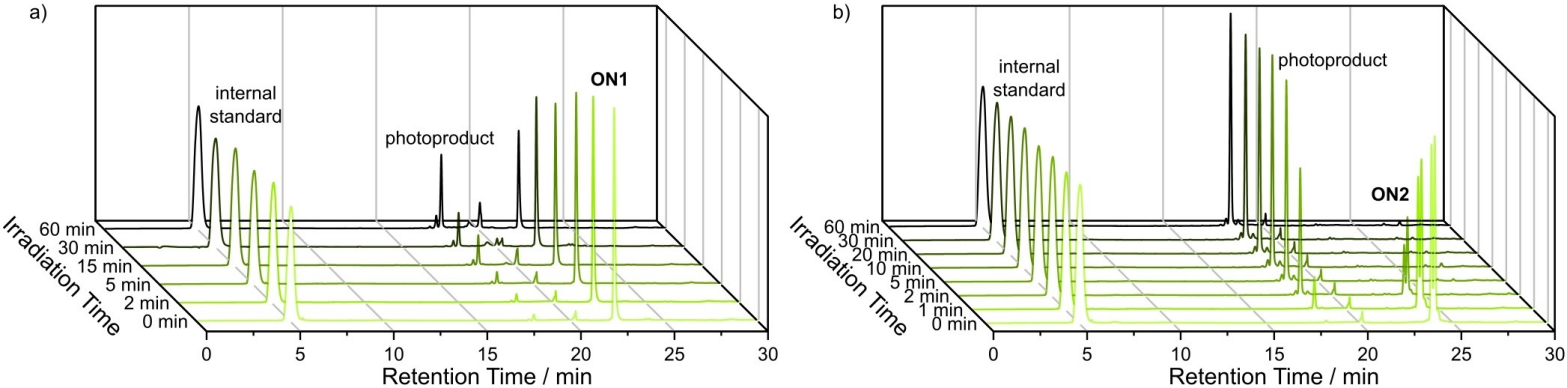
Photolysis comparison of a) BODIPY‐capped oligonucleotide **ON1** and b) coumarin‐capped oligonucleotide **ON2** over the time course of 60 min, irradiated simultaneously under the same conditions (13 μL, 83 μM, 27 mW, 530 nm LED).

Equal amounts of 1x PBS‐buffered oligonucleotide solutions were pipetted in separate wells and irradiated for multiple time periods (13 μL, 83 μM, 27 mW). After irradiation, the samples were analyzed by HPLC (Figure 2) and mass spectrometry. A strong peak for the photoproduct was detected after only 2 min of irradiation in the case of coumarin‐caged **ON2**. In the case of BODIPY‐caged **ON1**, the same signal was visible and after 60 min approximately half of the amount of **ON1** was uncaged. **ON2** on the other hand showed faster cleavage and deprotection was complete after only 5 min. The faster cleavage of the coumarin cage could be validated by photorelease quantum yield determination of **ON3** in 1x PBS giving 0.26 % (Table 1, Figure S4b, Figure S5b).

**Figure 3:**
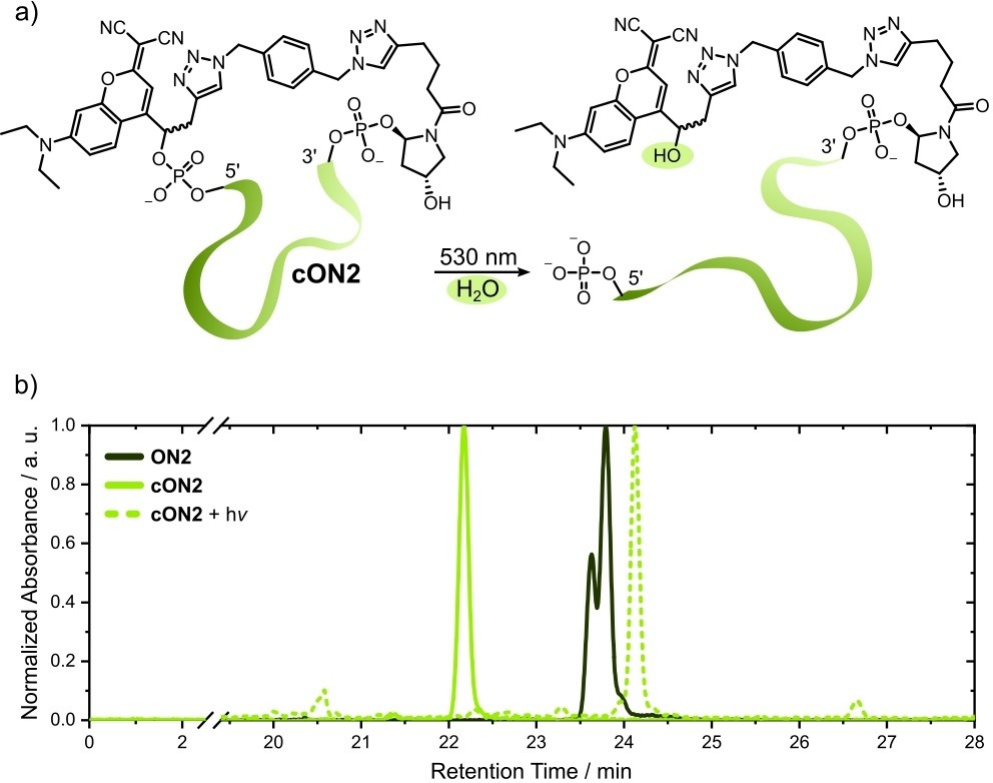
a) Uncaging mechanism of **cON2** with addition of water as proof for cyclization. b) RP‐HPLC chromatograms of the uncaging process of **cON2** in comparison to its linear form **ON2**.

Alkyne groups in biomolecules allow for a variety of bioconjugation reactions with CuAAC click chemistry. One of them is oligonucleotide photocaging by photo‐tethering, a method shown to be very effective for rapid and easy oligonucleotide caging.[^37^, ^38^] Any bidentate azide can act as a potential photo‐tether for **ON1** or **ON2** and allow end‐to‐end cyclization. Both oligonucleotides reacted readily under Cu‐catalysis in presence of 1,4‐bis(azidomethyl)benzene (AMB) to cyclic oligonucleotides **cON1** and **cON2**. A shift to smaller retention times in the HPLC chromatograms indicated the conformational change in the oligonucleotide and irradiation of that newly formed product at 530 nm resulted in another shift to even higher retention times than the linear starting materials (Figure 3b, Figure S8).

The reason for this shift to higher retention times is that uncaging of the photo‐tethered product results in an oligonucleotide where the caging group is released from the 5’‐end but still linked to the triazole moiety at the 3’‐end.

One problem with symmetric CuAAC photo‐tethering is that there is no change in mass in the second intramolecular cycloaddition. This makes it difficult to prove that the oligonucleotide has in fact been cyclized. Also in our case, the still linear product of the first cycloaddition step shows the same mass as the desired cyclic product.

Following the postulated uncaging mechanisms for BODIPYs and coumarins,[^23^, ^46^] the photoproducts of our cyclic oligonucleotides have a 17 Da higher mass due to photosolvolysis (Figure 3a). Photolysis of the linear oligonucleotides would simply lead to a loss of the BODIPY or coumarin moiety from the oligonucleotides. In our experiments, the peak at >24 min (Figure 3b) still showed absorbance at 500 nm and the expected mass difference that comes with the addition of water. This simple uncaging experiment not only demonstrated that relinearization and therefore modulation of the oligonucleotide's potential activity is possible but also at the same time served as a proof for successful cyclization in the first place.

Besides intramolecular photo‐tethering, any kind of azide‐modified molecule can be introduced at the 5’‐end in an intermolecular CuAAC reaction with the herein presented 5’‐caps. Tang et al. recently showed good modulation of siRNA activity by attaching a single vitamin E moiety to the 5’‐end of siRNA.^[47]^ In combination with a photocleavable unit between the last nucleotide and the vitamin E moiety, siRNA activity was fully restored after irradiation with UV light in their experiments. Using our 5’‐caps, siRNA modulation would be possible with harmless visible light.

Another alternative caging concept to photo‐tethering or steric blocking by bulky residues is the incorporation of strand break units into oligonucleotides.^[36]^ When a sense strand is linked to a complementary antisense strand via such a photocleavable loop, light‐activation causes cleavage. Subsequent dissociation of the antisense fragment then frees the active sense strand.

**Figure 4:**
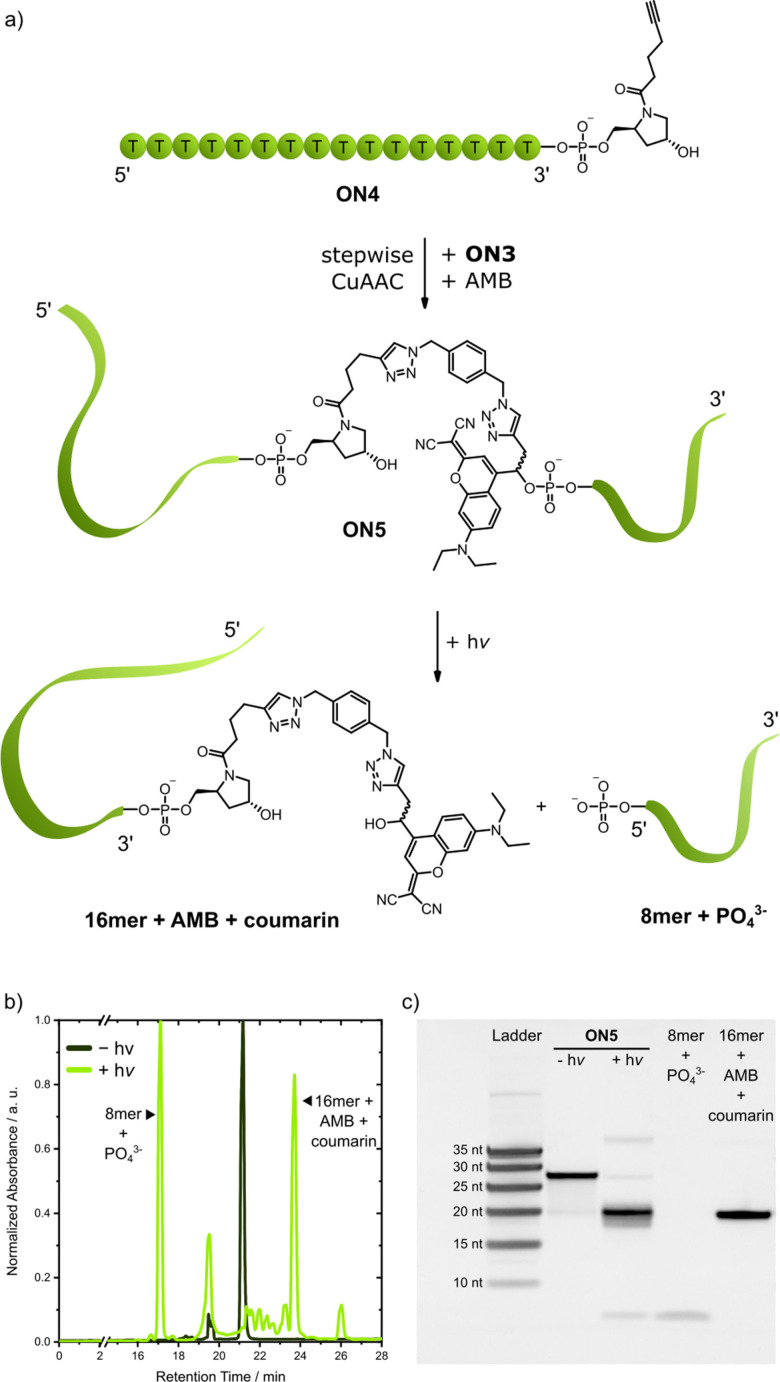
a) CuAAC ligation of **ON4**, **ON3** and AMB. Photolysis results in the smaller fragments shown. b) HPLC chromatogram of **ON5** photolysis (internal uridine standard not shown). c) 20 % denaturing polyacrylamide gel of **ON5** before and after irradiation. The photoproducts (8mer+PO_4_^3−^, 16mer+AMB+coumarin) found in HPLC were applied to the gel as a reference.

To adapt this approach to our 5’‐caps, we synthesized **ON4** with the previously used hydroxyprolinol alkyne modification at the 3’‐end (Figure 4a). For the coupling to **ON3** containing the photolabile 5’‐cap, we had to use a stepwise approach that was inspired by previous work of Seela et al.^[48]^ To that end, **ON4** was at first conjugated to AMB. To prevent dimerization, an excess of AMB was used and the concentration of **ON4** was lower than in photo‐tethering reactions. In the second step, AMB‐conjugated **ON4** was then coupled to **ON3**, affording **ON5**. As confirmed by PAGE, HPLC, and mass spectrometry, irradiation at 530 nm cleaved the oligonucleotide and released the expected shorter fragments (Figure 4b, c).

**Figure 5:**
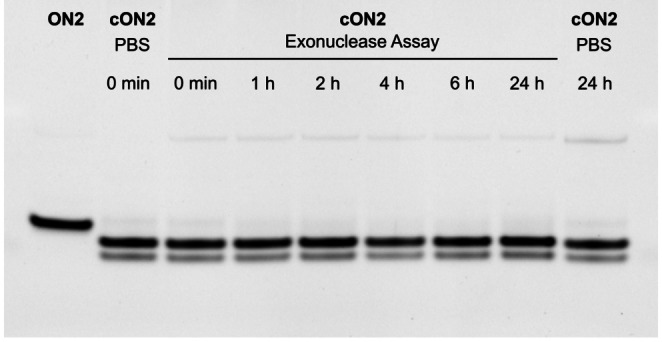
20 % denaturing polyacrylamide gel of photo‐tethered **cON2** after incubation with exonuclease VII at 37 °C.

We could show that photo‐tethering and CuAAC‐conjugation is possible with our newly designed photocages. Additionally, the herein presented photocaging method enhances the stability of oligonucleotides against exonucleases. Exonucleases are ubiquitous and one of the main reasons for the poor stability of exogenously applied oligonucleotides in plants or animals. The addition of non‐natural nucleotides at the 3’‐ and 5’‐ends shields the oligonucleotide from exonuclease attacks and cyclization has been shown to enhance stability of oligonucleotides.[^40^, ^41^] To demonstrate the stability of our photo‐tethered oligonucleotide, we incubated **ON2** and **cON2** with exonuclease VII at 37 °C for up to 24 h and compared it to a linear poly‐dT 20mer as a control. Different time points were taken and imaged in a polyacrylamide gel. While the native 20mer was already fully digested after the shortest time point taken after 1 h, modified **ON2** showed much higher stability simply due to 3’‐ and 5’‐modification alone (Figure S9). Photo‐tethering of **ON2** seemed to have an additional stabilizing effect where gel bands remained strong and sharp for 24 h (Figure 5). The second, weaker appearing band visible in the gel could be attributed to minor amounts of alkyne‐alkyne coupled Glaser side product.^[49]^ The formation of this Glaser product could be provoked by incubation of **ON2** under CuAAC conditions without AMB linker (Figure S10b). AMB‐linked **cON2** and the Glaser product both showed the same retention time in RP‐HPLC but could be distinguished by ESI‐MS. However, both oligonucleotides are cyclic versions of **ON2** and therefore show higher stability in the exonuclease VII assay. Also, irradiation of both oligonucleotides with green light resulted in relinearization (Figure S10a, c). Enhanced stability achieved through the chosen modification can be advantageous when later applying the cages and the caging concept to antisense oligonucleotides used in vivo.

## Conclusion

In summary, we have demonstrated the synthesis of two green‐light activatable 5’‐caps that are multifunctional in use through the possibility of copper‐catalyzed bioconjugation at the incorporated alkyne group. Both the BODIPY‐based as well as the coumarin‐based photocages showed sufficient stability in solid‐phase synthesis for direct incorporation in oligonucleotides. Isolation of the full‐length product was successful under UltraMild conditions. Photolysis of both photocages could then be induced with LEDs at 530 nm and 565 nm.

CuAAC reactions were used for chemical ligation as well as cyclization of oligonucleotides. After ligation, the resulting longer oligonucleotide carried an internal strand break unit that allowed separation of the two fragments by light irradiation. Photo‐tethering on the other hand resulted in cyclic oligonucleotides that could be relinearized again with green light and showed high stability against exonuclease VII. Different caging strategies can therefore be easily accessed and allow a wide variety of application fields. The alkyne functionality of the cages makes them a versatile tool for bioconjugation to any azide‐containing molecule while at the same time offering their control with visible light. Both caging groups are placed within the green gap of plants and considered as promising candidates to further drive bioorthogonal and spatio‐temporal gene regulation in milder wavelength regions than traditional UV cages, especially for photoregulation in plants. This will be the subject of further studies from our group.

## Experimental Section

### Synthesis

In general, all reactions were performed in dry solvents under an argon atmosphere unless otherwise stated. Solvents and reagents were purchased from commercial sources. Reaction progress was monitored by TLC (silica gel 60‐coated aluminum sheets, UV254 marker, Macherey‐Nagel) and crude products were purified by column chromatography (silica gel 60, Macherey‐Nagel). NMR spectra were recorded on Bruker Instruments (400 MHz, 500 MHz, or 600 MHz) and ESI mass spectra were obtained on a ThermoFisher Surveyor MSQ. High‐resolution mass spectrometry (HRMS) was performed on a LTQ Orbitrap XL by ThermoScientific.

### Photochemical Measurements

Absorption and fluorescence spectra were recorded in quartz glass cuvettes with either 10 mm or 2 mm optical path length (Hellma Analytics) on a Jasco V‐650 UV‐vis spectrophotometer and a Hitachi F‐4500 fluorometer. Absorption spectra were measured at an optical density (OD) close to 1. OD values for fluorescence spectra measurements were set to 0.1 to 0.15. Data for **ON1** was obtained on a Tecan Infinite M200 Pro plate reader.

Uncaging quantum yields were determined using our recently published fulgide actinometry setup controlled by our in‐house programmed software PHITS (Photoswitch Irradiation Test Suite) written with LabVIEW.^[43]^ A concentrated indolylfulgide photoswitch solution in toluene served as a reference for the chemical actinometry. Its absorption spectrum was tracked (Ocean Optics DH‐mini light source, Ocean Optics USB4000 or Thorlabs CCS200/M detector) while converting the fulgide from its closed form to the *Z*‐form through irradiation with a 530 nm LED (M530 L3, Thorlabs). The caged oligonucleotide was then irradiated in the same setup with known photon flux. Last, the photolysis rate was determined by integration of the RP‐HPLC signals.

### Oligonucleotide Synthesis

All oligonucleotides were synthesized on an ABI 392 DNA/RNA synthesizer at a 1 μmol scale using UltraMild phosphoramidites in combination with UltraMild capping reagents (emp Biotech). 0.3 M BTT (emp Biotech) was used as an activator. Standard coupling protocols with DMTon strategy were used and coupling times for the 5’‐cap phosphoramidites were extended to 12 min. Cleavage from the solid support was carried out with 0.05 M K_2_CO_3_ solution in MeOH within 4 h. The cleavage solution was then desalted with illustra NAP columns (GE Healthcare) and RNase‐free water. Purification of the resulting oligonucleotide solution was carried out by RP‐HPLC after vacuum concentration. ESI‐MS was performed on a Bruker micrOTOF‐QII.

## Conflict of interest

The authors declare no conflict of interest.

1

## Supporting information

As a service to our authors and readers, this journal provides supporting information supplied by the authors. Such materials are peer reviewed and may be re‐organized for online delivery, but are not copy‐edited or typeset. Technical support issues arising from supporting information (other than missing files) should be addressed to the authors.

Supporting Information

## Data Availability

The data that support the findings of this study are available in the supplementary material of this article.
