# Supplementary material for: Green‐Light Activatable BODIPY and Coumarin 5’‐Caps for Oligonucleotide Photocaging
Source: Chemistry. 2022 May 17;28(36):e202200477. doi: 10.1002/chem.202200477 (PMC9322594; doi:10.1002/chem.202200477)
Supplement: Supplementary file 1 — Supporting Information [file CHEM-28-0-s001.pdf]

Chemistry–A European Journal

Supporting Information

Green-Light Activatable BODIPY and Coumarin 5′-Caps for Oligonucleotide Photocaging

Janik Kaufmann, Patricia Müller, Eleni Andreadou, and Alexander Heckel*

Table of Contents

1 Chemical Synthesis 2

1.1 Materials and Methods 2

1.2 BODIPY Synthesis 3

1.3 Coumarin Synthesis 6

2 Oligonucleotide Synthesis 12

3 Photochemical Experiments 15

3.1 Absorption and Fluorescence Spectra 15

3.2 Actinometry and Photolysis Quantum Yield 16

3.3 Uncaging Experiments 18

4 CuAAC Click Reactions 21

5 Exonuclease VII Stability Assay 23

6 NMR Spectra 25

6.1 BODIPY NMR Spectra 25

6.2 Coumarin NMR Spectra 29

7 Mass Spectra 37

7.1 BODIPY Mass Spectra 37

7.2 Coumarin Mass Spectra 39

7.3 Oligonucleotide Mass Spectra 43

8 References 46

1 Chemical Synthesis

1.1 Materials and Methods

Reactions where dry solvents were involved were performed under a protective argon atmosphere. All reagents and solvents were purchased from commercial sources and used without further purification. Dry solvents were purchased over molecular sieves (Acros Organics).

Reactions were monitored using silica gel 60-coated TLC sheets and silica gel 60 (0.04–0.063 mm) was used for purification by silica gel columns. For the purification of phosphoramidites, the silica gel column was washed with the respective eluent containing 1% triethylamine (TEA) before application of the crude product.

NMR spectra were recorded in $CDCl_3$ or dimethyl sulfoxide-d_6 (DMSO-d_6) on a Bruker Avance AV 400 MHz, Avance III HD AV500 MHz, or an Avance DRX600 MHz spectrometer at room temperature. All shifts are reported in ppm using the solvent signal as an internal reference (^{1}H: 7.26 ppm $CDCl_3$, 2.50 ppm DMSO-d_6; ^{13}C: 77.16 ppm $CDCl_3$, 39.52 ppm DMSO-d_6). $CDCl_3$ was filtered through basic Al_2O_3 before phosphoramidite spectra were recorded. Following abbreviations were used to describe multiplicities: s = singlet, d = doublet, t = triplet, q = quartet, m = multiplet, (b) = broad signal. Coupling constants are reported in hertz (Hz).

Electrospray ionization (ESI) mass spectra were obtained on a Thermo Fisher Surveyor MSQ device and high-resolution mass spectrometry (HRMS) was conducted on a LTQ Orbitrap XL by Thermo Fisher.

Stability Tests Against Solid-Phase Reagents

To estimate the stabilities of the synthesized BODIPY and coumarin derivatives **4** and **13** in solid-phase synthesis, small amounts of the named compounds were dissolved in dichloromethane (DCM). 50 µL of the respective solution was pipetted to 50 µL of commonly used solid-phase reagents (0.05 M K_2CO_3 in MeOH, 80% AcOH, BTT Activator, Oxidizer, 3% DCA in DCM (Deblock)) and shaken for 15 min at room temperature. All samples were compared to the starting material by TLC.

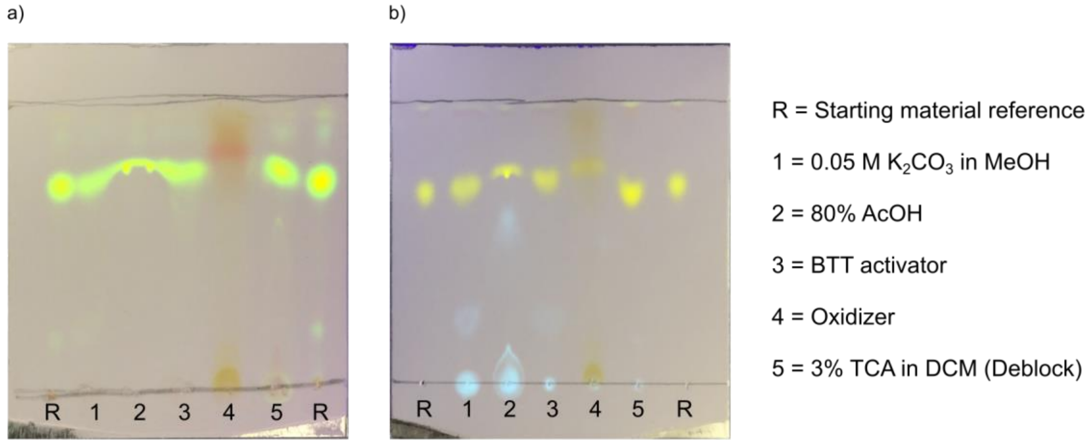

*Figure S1: Stability against solid-phase reagents of a) BODIPY alcohol **4** (DCM) and b) coumarin alcohol **13** (cyclohexane/ethyl acetate 3:2) after 15 min at room temperature.*

1.2 BODIPY Synthesis

BODIPY derivatives **1**, **2** and **3** were synthesized following published procedures.[1]

8-Acetoxymethyl-4,4'-difluoro-1,3,5,7-tetramethyl-4-bora-3a,4a-diaza-*s*-indacene (1)

2,4-Dimethylpyrrole (3.00 mL, 29.1 mmol, 2.0 eq) was dissolved in 50 mL dry DCM and refluxed for 2 h after addition of acetoxyacetyl chloride (1.88 mL, 17.5 mmol, 1.2 eq). The solution was cooled to room temperature and *N*,*N*-diisopropylethylamine (DIPEA) (9.9 mL, 58 mmol, 4.0 eq) was added. After 15 min, boron trifluoride diethyl etherate (7.4 mL, 58 mmol, 4.0 eq) was added over a period of 10 min. After another 15 min at room temperature, the solvent was removed under reduced pressure. The dark crude product was purified by column chromatography (cyclohexane/ethyl acetate 3:1). Compound **1** was obtained as a red solid.

Yield: 1.72 g (5.37 mmol, 37%).

TLC: R_f = 0.58 (cyclohexane/ethyl acetate 3:1).

^{1}H-NMR: (400 MHz, $CDCl_3$): δ (ppm) = 6.09 (s, 2 H, H-2, H-6), 5.30 (s, 2 H, CH_2OAc), 2.53 (s, 6 H, 2x CH_3), 2.36 (s, 6 H, 2x CH_3), 2.13 (s, 3 H, CH_3COO).

ESI-MS: *m/z* calculated for $C_{16}H_{18}BF_2N_2O_2$ $[M-H]^-$ 319.14, found 319.23.

4,4'-Difluoro-8-hydroxymethyl-1,3,5,7-tetramethyl-4-bora-3a,4a-diaza-*s*-indacene (2)

180 mL MeOH was mixed with 38 mL of a 0.1 M NaOH solution (0.4 eq) and stirred at room temperature for 10 min. The mixture was then added to a solution of compound **1** (3.03 g, 9.46 mmol, 1.0 eq) in 80 mL DCM and stirred for an additional 5 h. MeOH was removed under reduced pressure and the residue was extracted with ethyl acetate. The combined organic layers were washed with 1 M HCl and brine and dried over Na_2SO_4. After removal of the solvent and purification by silica gel column chromatography (cyclohexane/ethyl acetate 2:1), compound **2** was obtained as a red solid.

Yield: 2.00 g (7.19 mmol, 76%).

TLC: R_f = 0.36 (cyclohexane/ethyl acetate 2:1).

^{1}H-NMR: (600 MHz, $CDCl_3$): δ (ppm) = 6.09 (s, 2 H, H-2, H-6), 4.91 (s, 2 H, C*H₂*OH), 2.53 (s, 6 H, 2x CH_3), 2.51 (s, 6 H, 2x CH_3).

ESI-MS: *m/z* calculated for $C_{14}H_{16}BF_2N_2O$ $[M-H]^-$ 277.13, found 277.20.

4,4'-Difluoro-8-formyl-1,3,5,7-tetramethyl-4-bora-3a,4a-diaza-*s*-indacene (3)

To a cooled solution of Dess-Martin periodinane (3.27 g, 7.71 mmol, 1.5 eq) in 60 mL dry DCM, a solution of alcohol **2** (1.43 g, 5.14 mmol, 1.0 eq) in 25 mL dry DCM was slowly added over a period of 10 min at 0 °C. The mixture was stirred for 10 min at 0 °C and the ice bath was removed. A second portion of Dess-Martin periodinane (3.27 g, 7.71 mmol, 1.5 eq) was added after 1 h at room temperature. After an additional 30 min at room temperature, the mixture was washed with conc. $Na_2S_2O_3$ solution, conc. $NaHCO_3$ solution and brine. The combined organic layers were then dried over Na_2SO_4 and the solvent was removed under reduced pressure. Silica gel column chromatography (DCM) gave aldehyde **3** as a purple solid.

Yield: 779 mg (2.28 mmol, 55%).

TLC: R_f = 0.87 (DCM).

^{1}H-NMR: (600 MHz, $CDCl_3$): δ (ppm) = 10.57 (s, 1 H, C*H*O), 6.08 (s, 2 H, H-2, H-6), 2.54 (s, 6 H, 2x CH_3), 2.13 (s, 6 H, 2x CH_3).

4,4’-Difluoro-8-(1-hydroxybut-3-in-1-yl)-1,3,5,7-tetramethyl-4-bora-3a,4a-diaza-*s*-indacene (4)

Activated zinc granules (461 mg, 7.05 mmol, 3.3 eq) were suspended in 10 mL dry dimethylformamide (DMF) and cooled to 0 °C. Propargyl bromide (0.34 mL, 3.3 mmol, 1.5 eq) was added and the mixture was then stirred at 0 °C. After 1 h, BODIPY aldehyde **3** (590 mg, 2.14 mmol, 1.0 eq) was dissolved in 15 mL dry DMF and added to the mixture. The reaction was stopped by addition of 5 mL conc. NH_4Cl solution after an additional 30 min at 0 °C. The mixture was extracted with diethyl ether four times and the combined organic layers were dried with Na_2SO_4. After removal of the solvent under reduced pressure, compound **4** was obtained as a red solid without further purification.

Yield: 676 mg (2.14 mmol, quantitative).

TLC: R_f = 0.61 (DCM).

^{1}H-NMR: (500 MHz, $CDCl_3$): δ (ppm) = 6.09 (s, 2 H, H-2, H-6), 5.65 (m, 1 H, C*H*OH), 2.98 (ddd, *J* = 17.3 Hz, 10.2 Hz, 2.6 Hz, 1 H, CH_2CCH), 2.65 (ddd, *J* = 17.3 Hz, 4.4 Hz, 2.7 Hz, 1 H, CH_2CCH), 2.56 (d, *J* = 2.9 Hz, 1 H, O*H*), 2.51–2.49 (m, 12 H, 4x CH_3), 2.18 (t, *J* = 2.6 Hz, 1 H, CH_2CC*H*)

^{11}B{^{1}H}-NMR: (160 MHz, $CDCl_3$): δ (ppm) = 0.5 (t, *J* = 32.8 Hz).

^{13}C{^{1}H}-NMR: (126 MHz, $CDCl_3$): δ (ppm) = 156.0 (C-3, C-5), 143.6 (C-8), 142.0 (C-1, C-7), 131.0 (C-7a, C-8a), 123.4 (C-2, C-6), 80.0 (*C*CH), 72.0 (C*C*H), 67.0 (*C*OH), 26.9 (*C*H_2), 18.4 (C*C*H_3), 14.8 (C*C*H_3).

^{19}F-NMR: (471 MHz, $CDCl_3$): δ (ppm) = −146.1 – −146.4 (m).

MALDI-HRMS: *m/z* calculated for $C_{17}H_{19}BF_2N_2O$ $[M\cdot]^+$ 316.15585, found 316.15544 (Δ_m = 0.00041, Δ_m/m = 1.3 ppm).

2-Cyanoethyl-[1-(4,4’-difluoro-1,3,5,7-tetramethyl-4-bora-3a,4a-diaza-*s*-indacene-8-yl)but-3-yn-1-yl]-*N,N*-diisopropylphosphoramidite (5)

DIPEA (0.30 mL, 1.7 mmol, 5.0 eq) was added to a solution of BODIPY alcohol **9** (109 mg, 345 µmol, 1.0 eq) in 12 mL dry DCM. After 5 min, 2-cyanoethyl *N,N*’-diisopropylchlorophosphoramidite (0.15 mL, 0.69 mmol, 2.0 eq) was added and the solution was stirred for an additional 3 h at room temperature. The reaction mixture was washed with conc. $NaHCO_3$ solution and the aqueous phase was extracted with DCM three times. The combined

organic layers were dried with Na_2SO_4 and the solvent was then removed under reduced pressure. Purification by silica gel column chromatography (DCM) gave phosphoramidite **5** as a red solid.

Yield: 154 mg (298 µmol, 87%).

TLC: R_f = 0.67 (cyclohexane/ethyl acetate 2:1).

^{1}H-NMR: (500 MHz, $CDCl_3$): δ (ppm) = 6.11–6.07 (m, 2 H, H-2, H-6), 5.93–5.85 (m, 1 H, C*H*CH_2CCH), 3.97 (quart, 1 H, *J* = 6.80 Hz, POC*H$_2$*), 3.63–3.56 (m, 1 H, NC*H*(Me)$_2$), 3.55–3.48 (m, 1 H, POC*H$_2$*), 3.42–3.34 (m, 1 H, NC*H*(Me)$_2$), 3.10–3.00 (m, 1 H, C*H$_2$*CCH), 2.79–2.67 (m, 1 H, C*H$_2$*CCH), 2.65–2.64 (m, 1 H, C*H$_2$*CN), 2.58–2.50 (m, 12 H, 4x C*H$_3$*), 2.32–2.28 (m, 1 H, C*H$_2$*CN), 2.12–2.02 (m, 1 H, CH_2CC*H*), 1.22–0.79 (m, 12 H, 4x N(CH(C*H$_3$*)$_2$)$_2$).

^{11}B{^{1}H}-NMR: (160 MHz, $CDCl_3$): δ (ppm) = 0.4 (t, *J* = 32.3 Hz).

^{13}C{^{1}H}-NMR: (126 MHz, $CDCl_3$): δ (ppm) = 156.0 (C-3, C-5), 155.9 (C-3, C-5), 155.8 (C-3, C-5), 155.7 (C-3, C-5), 143.3 (C-8), 143.2 (C-8), 142.6 (C-8), 142.4 (C-8), 141.2 (C-1, C-7), 140.6 (C-1, C-7), 131.2 (C-7a, C-8a), 131.1 (C-7a, C-8a), 131.0 (C-7a, C-8a), 130.6 (C-7a, C-8a), 123.4 (C-2, C-6), 123.3 (C-2, C-6), 123.0 (C-2, C-6), 117.7 (*C*N), 117.6 (*C*N), 80.0 (*C*CH), 79.6 (*C*CH), 71.4 (C*C*H), 71.3 (C*C*H), 69.1 (*C*HCH_2CCH), 68.9 (*C*HCH_2CCH), 67.8 (*C*HCH_2CCH), 67.6 (*C*HCH_2CCH), 59.6 (PO*C*H$_2$), 59.5 (PO*C*H$_2$), 58.7 (PO*C*H$_2$), 58.5 (PO*C*H$_2$), 43.8 (PN(*C*H(CH_3)$_2$)$_2$), 43.7 (PN(*C*H(CH_3)$_2$)$_2$), 26.4 (CH*C*H$_2$CCH), 26.3 (CH*C*H$_2$CCH), 26.2 (CH*C*H$_2$CCH), 24.9 (PN(CH(*C*H$_3$)$_2$)$_2$), 24.8 (PN(CH(*C*H$_3$)$_2$)$_2$), 24.7 (PN(CH(*C*H$_3$)$_2$)$_2$), 24.6 (PN(CH(*C*H$_3$)$_2$)$_2$), 24.5 (PN(CH(*C*H$_3$)$_2$)$_2$), 23.5 (PN(CH(*C*H$_3$)$_2$)$_2$), 20.5 (*C*H$_2$CN), 20.4 (*C*H$_2$CN), 19.9 (*C*H$_2$CN), 18.8 (C*C*H$_3$), 18.6 (C*C*H$_3$), 18.4 (C*C*H$_3$), 14.8 (C*C*H$_3$), 14.7 (C*C*H$_3$).

^{19}F-NMR: (471 MHz, $CDCl_3$): δ (ppm) = −145.4 – −146.9 (m).

^{31}P{^{1}H}-NMR: (202 MHz, $CDCl_3$): δ (ppm) = 153.1 (s), 152.2 (s).

MALDI-HRMS: *m/z* calculated for $C_{26}H_{37}BF_2N_4O_2P$ $[M+H]^+$ 517.27154, found 517.27090 (Δ_m = 0.00064, Δ_m/m = 1.2 ppm).

1.3 Coumarin Synthesis

Synthesis of coumarin derivatives **7** and **8** were synthesized as published by Göbel *et al.*[2] and the alkyne derivative **9** was prepared as previously described by our group.[3]

(*E*)-7-(Diethylamino)-4-[2-(dimethylamino)vinyl]-*2H*-chromen-2-one (7)

7-Diethylamino-4-methylcoumarin **6** (80.0 g, 346 mmol, 1.0 eq) was dissolved in 700 mL dry DMF. After addition of *N*,*N*-dimethylformamide dimethyl acetal (69.2 mL, 519 mmol, 1.5 eq), the solution was refluxed for 8 h and then stirred at room temperature overnight. The reaction mixture was washed with conc. $NaHCO_3$ solution and after extraction of the aqueous phase with DCM, the combined organic layers were dried over Na_2SO_4. Removal of the solvent under reduced pressure gave compound **7** as a brown solid that was used without further purification.

Yield: 93.00 g (crude).

TLC: R_f = 0.73 (DCM/MeOH 9:1).

^{1}H-NMR: (500 MHz, $CDCl_3$): δ (ppm) = 7.52 (d, *J* = 9.1 Hz, 1 H, H-5), 7.21 (d, *J* = 13.0 Hz, 1 H, $Me_2NC\mathit{H}CH$), 6.55 (dd, *J* = 9.0 Hz, 2.7 Hz, 1 H, H-6), 6.40 (d, *J* = 2.7 Hz, 1 H, H-8), 5.85 (s, 1 H, H-3), 5.22 (d, *J* = 13.0 Hz, 1 H, $Me_2NCHC\mathit{H}$), 3.39 (q, *J* = 7.1 Hz, 4 H, $N(C\mathit{H_2}Me)_2$), 2.99 (s, 6 H, $(C\mathit{H_3})_2NCHCH$), 1.19 (t, *J* = 7.1 Hz, 6 H, $N(CH_2C\mathit{H_3})_2$).

ESI-MS: *m/z* calculated for $C_{17}H_{23}N_2O_2$ $[M+H]^+$ 287.18, found 287.11.

7-(Diethylamino)-2-oxo-*2H*-chromene-4-carbaldehyde (8)

Coumarin **7** (93.00 g, 324.8 mmol, 1.0 eq) was suspended in 1.5 L tetrahydrofuran/H_2O (1:1) and $NaIO_4$ (208.37 g, 974.19 mmol, 3.0 eq) was added while the suspension was stirred vigorously. After 4 h at room temperature, the reaction mixture was filtered through silica gel. The silica gel was washed with ethyl acetate and the organic phase was concentrated under reduced pressure. The organic phase was then washed with conc. $NaHCO_3$ solution and the aqueous layer was extracted with DCM five times. Afterward, the organic layers were dried over Na_2SO_4. Removal of the solvent under reduced pressure gave aldehyde **8** as a brown oil. The crude product was used without further purification.

Yield: 81.93 g (crude).

TLC: R_f = 0.44 (DCM/ethyl acetate 19:1).

^{1}H-NMR: (400 MHz, $CDCl_3$): δ (ppm) = 10.03 (s, 1 H, CHO), 8.32 (d, *J* = 9.2 Hz, 1 H, H-5), 6.68 (dd, *J* = 9.2 Hz, 2.6 Hz, 1 H, H-6), 6.55 (d, *J* = 2.6 Hz, 1 H, H-8), 6.46 (s, 1 H, H-3), 3.43 (q, *J* = 7.1 Hz, 4 H, $N(C\mathit{H_2}Me)_2$), 1.22 (t, *J* = 7.1 Hz, 6 H, $N(CH_2C\mathit{H_3})_2$).

ESI-MS: *m/z* calculated for $C_{14}H_{16}NO_3$ $[M+H]^+$ 246.11, found 246.03.

7-(Diethylamino)-4-(1-hydroxybut-3-in-1-yl)-*2H*-chromen-2-one (9)

Activated zinc granules (3.40 g, 51.9 mmol, 2.0 eq) were suspended in 96 mL dry DMF and cooled to 0 °C. Propargyl bromide (4.0 mL, 39 mmol, 1.5 eq) was added and the mixture was stirred at 0 °C for 1 h. A solution of coumarin aldehyde **8** (6.37 g, 26.0 mmol, 1.0 eq) in 20 mL dry DMF was then added to the mixture and stirred for an additional 30 min at 0 °C. The reaction was stopped by addition of conc. NH_4Cl solution and the aqueous phase was extracted with diethyl ether. The combined organic layers were dried with Na_2SO_4 before the solvent was removed under reduced pressure. Purification by silica gel column chromatography (DCM/acetone 10:1) gave alcohol **9** as a brown oil.

Yield: 4.33 g (15.1 mmol, 58%).

TLC: R_f = 0.45 (DCM/acetone 9:1).

^{1}H-NMR: (400 MHz, $CDCl_3$): δ (ppm) = 7.47 (d, *J* = 9.0 Hz, 1 H, H-5), 6.80–6.78 (m, 1 H, H-6), 6.68 (s, 1 H, H-8), 6.37 (s. 1 H, H-3), 5.15–5.13 (m, 1 H, C*H*OH), 3.43 (q, *J* = 7.1 Hz, 4 H, N(CH_2Me)$_2$), 2.84 (ddd, *J* = 17.0 Hz, 4.3 Hz, 2.7 Hz, 1 H, CH_2CCH), 2.70 (s (b), 1 H, OH), 2.66 (ddd, *J* = 17.0 Hz, 7.5 Hz, 2.6 Hz, CH_2CCH), 2.18–2.17 (m, 1 H, CH_2CC*H*), 1.22 (t, *J* = 7.1 Hz, 6 H, N(CH_2CH_3)$_2$).

ESI-MS: *m/z* calculated for $C_{17}H_{20}NO_3$ $[M+H]^+$ 286.14, found 286.01.

4-{1-[(*tert*-Butyldimethylsilyl)oxy]but-3-yn-1-yl}-7-(diethylamino)-*2H*-chromen-2-one (10)

Coumarin alcohol **9** (4.33 g, 15.2 mmol, 1.0 eq) was dissolved in 55 mL dry DMF together with *tert*-butyldimethylsilyl chloride (9.15 g, 60.7 mmol, 4.0 eq) and imidazole (7.23 g, 106 mmol, 7.0 eq) and stirred for 72 h at room temperature. 20 mL ethanol were added to stop the reaction before the solvent was removed under reduced pressure. The residue was dissolved in 300 mL ethyl acetate and subsequently washed with H_2O, 1 M HCl and conc. $NaHCO_3$ solution. The combined organic layers were dried over Na_2SO_4 and the solvent was removed under reduced pressure. Compound **10** was obtained as a brown solid after silica gel column chromatography (cyclohexane/acetone 3:1).

Yield: 4.52 g (11.3 mmol, 75%).

TLC: R_f = 0.45 (DCM/acetone 9:1).

^{1}H-NMR: (500 MHz, $CDCl_3$): δ (ppm) = 7.52 (d, *J* = 9.0 Hz, 1 H, H-5), 6.64–6.63 (m, 1 H, H-6), 6.57 (s, 1 H, H-8), 6.24 (s, 1 H, H-3), 5.00 (dd, *J* = 7.2 Hz, 4.3 Hz, 1 H, C*H*OH), 3.41 (q, *J* = 7.1 Hz, 4 H, N(CH_2Me)$_2$), 2.67 (ddd, *J* = 16.9 Hz, 4.7 Hz, 2.6 Hz, 1 H, CH_2CCH), 2.60 (ddd,

J = 16.9 Hz, 7.3 Hz, 2.7 Hz, 1 H, C*H*$_2$CCH), 2.03 (t, J = 2.7 Hz, 1 H, CH$_2$CC*H*), 1.21 (t, J = 7.1 Hz, N(CH$_2$C*H*$_3$)$_2$). 0.92 (s, 9 H, Si(CH$_3$)$_2$C(C*H*$_3$)$_3$), 0.14 (s, 3 H, Si(C*H*$_3$)$_2$C(CH$_3$)$_3$), 0.02 (s, 3 H, Si(C*H*$_3$)$_2$C(CH$_3$)$_3$).

<u>^{13}C{^{1}H}-NMR:</u> (126 MHz, CDCl$_3$): δ (ppm) = 162.3 (C-2), 156.7 (C-8a), 156.7 (C-4), 150.0 (C-7), 125.3 (C-5), 109.1 (C-6), 107.0 (C-3), 106.6 (C-4a), 98.8 (C-8), 80.6 (*C*CH), 71.3 (C*C*H), 70.6 (*C*HCH$_2$CCH), 45.3 (N(*C*H$_2$CH$_3$)$_2$), 29.1 (CH*C*H$_2$CCH), 25.9 (SiC(*C*H$_3$)$_3$), 18.3 (Si*C*(CH$_3$)$_3$), 12.5 (N(CH$_2$*C*H$_3$)$_2$), −4.7 (Si*C*H$_3$), −4.8 (Si*C*H$_3$).

<u>MALDI-HRMS:</u> *m/z* calculated for $C_{23}H_{34}NO_3Si$ [M+H]$^+$ 400.23080, found 400.22956 (Δ_m = 0.00069, Δ_m/m = 1.7 ppm).

4-{1-[(*tert*-Butyldimethylsilyl)oxy]but-3-yn-1-yl}-7-(diethylamino)-*2H*-chromene-2-thione (11)

Lawesson's reagent (2.97 g, 7.35 mmol, 0.65 eq) was added to a solution of TBDMS-protected coumarin alcohol **10** (4.52 g, 11.3 mmol, 1.0 eq) in 240 mL dry toluene. The mixture was refluxed for 22 h and stirred for an additional 48 h at room temperature. After cooling to room temperature, the solvent was removed under reduced pressure and the crude product was purified by silica gel column chromatography (DCM/cyclohexane 8:1) to give pure compound **11** as an orange solid.

<u>Yield:</u> 3.48 g (8.37 mmol, 74%).

<u>TLC:</u> R_f = 0.79 (DCM).

<u>^{1}H-NMR:</u> (500 MHz, CDCl$_3$): δ (ppm) = 7.62 (d, J = 9.2 Hz, 1 H, H-5), 7.11 (s, 1 H, H-3), 6.69 (d, J = 2.6 Hz, 1 H, H-8), 6.64 (dd, J = 9.1 Hz, 2.6 Hz, 1 H, H-6), 4.97 (t, J = 6.2 Hz, 1 H, C*H*OH), 3.42 (q, J = 7.1 Hz, 4 H, N(C*H*$_2$Me)$_2$), 2.65 (dd, J = 6.2 Hz, 2.6 Hz, 1 H, C*H*$_2$CCH), 2.03 (t, J = 2.6 Hz, 1 H, CH$_2$CC*H*), 1.22 (t, J = 7.1 Hz, 6 H, N(CH$_2$C*H*$_3$)$_2$). 0.91 (s, 9 H, Si(CH$_3$)$_2$C(C*H*$_3$)$_3$), 0.13 (s, 3 H, Si(C*H*$_3$)$_2$C(CH$_3$)$_3$), 0.01 (s, 3 H, Si(C*H*$_3$)$_2$C(CH$_3$)$_3$).

<u>^{13}C{^{1}H}-NMR:</u> (126 MHz, CDCl$_3$): δ (ppm) = 197.5 (C-2), 159.7 (C-8a), 150.9 (C-7), 149.0 (C-4), 125.7 (C-5), 120.9 (C-3), 110.1 (C-6), 108.2 (C-4a), 97.8 (C-8), 80.4 (*C*CH), 71.4 (C*C*H), 70.8 (*C*HCH$_2$CCH), 45.0 (N(*C*H$_2$CH$_3$)$_2$), 29.1 (CH*C*H$_2$CCH), 25.9 (SiC(*C*H$_3$)$_3$), 18.4 (Si*C*(CH$_3$)$_3$), 12.6 (N(CH$_2$*C*H$_3$)$_2$), −4.6 (Si*C*H$_3$), −4.8 (Si*C*H$_3$).

<u>MALDI-HRMS:</u> *m/z* calculated for $C_{23}H_{34}NO_2SSi$ [M+H]$^+$ 416.20741, found 416.20637 (Δ_m = 0.00104, Δ_m/m = 2.5 ppm).

2-(4-{1-[(*tert*-Butyldimethylsilyl)oxy]but-3-yn-1-yl}-7-(diethylamino)-*2H*-chromen-2-ylidene)malononitrile (12)

Coumarin **11** (3.48 g, 8.37 mmol, 1.0 eq) was heated to 90 °C in 200 mL dry toluene for 24 h together with 4-dimethylaminopyridine (2.05 g, 16.7 mmol, 2.0 eq), PbO (3.74 g, 16.7 mmol, 2.0 eq) and malononitrile (1.11 g, 16.7 mmol, 2.0 eq). The black suspension was then filtered through celite and the solvent was removed under reduced pressure. Purification by silica gel column chromatography (DCM/cyclohexane 7:4) gave dicyanocoumarin **12** as an orange solid.

Yield: 1.82 g (4.06 mmol, 49%).

TLC: R_f = 0.68 (DCM).

^{1}H-NMR: (500 MHz, $CDCl_3$): δ (ppm) = 7.54 (d, *J* = 9.2 Hz, 1 H, H-5), 6.89 (s, 1 H, H-3), 6.65 (dd, *J* = 9.2 Hz, 2.7 Hz, 1 H, H-6), 6.61 (d, *J* = 2.6 Hz, 1 H, H-8), 5.04 (dd, *J* = 6.6 Hz, 5.3 Hz, 1 H, C*H*OH), 3.45 (q, *J* = 7.2 Hz, 4 H, N(CH_2Me)$_2$), 2.66 (ddd, *J* = 16.9 Hz, 5.0 Hz, 2.7 Hz, 1 H, CH_2CCH), 2.61 (ddd, *J* = 16.9 Hz, 6.9 Hz, 2.7 Hz, 1 H, CH_2CCH), 2.06 (t, *J* = 2.7 Hz, 1 H, CH_2CC*H*), 1.24 (t, *J* = 7.1 Hz, 6 H, N(CH_2CH_3)$_2$), 0.93 (s, 9 H, Si(CH_3)$_2$C(CH_3)$_3$), 0.15 (s, 3 H, Si(CH_3)$_2$C(CH_3)$_3$), 0.04 (s, 3 H, Si(CH_3)$_2$C(CH_3)$_3$).

^{13}C{^{1}H}-NMR: (126 MHz, $CDCl_3$): δ (ppm) = 172.1 (C-2), 155.5 (C-8a), 153.6 (C-4), 151.5 (C-7), 125.8 (C-5), 114.7 (C*C*N), 114.2 (C*C*N), 110.4 (C-6), 107.0 (C-3), 106.3 (C-4a), 97.7 (C-8), 80.0 (*C*CH), 71.8 (C*C*H), 70.4 (*C*HCH_2CCH), 55.3 (*C*(CN)$_2$), 45.0 (N(*C*H_2CH_3)$_2$), 29.3 (CH*C*H_2CCH), 25.8 (SiC(*C*H_3)$_3$), 18.3 (Si*C*(CH_3)$_3$), 12.6 (N(CH_2*C*H_3)$_2$), −4.6 (Si*C*H_3), −4.8 (Si*C*H_3).

MALDI-HRMS: *m/z* calculated for $C_{26}H_{34}N_3O_2Si$ [M+H]$^+$ 448.24148, found 448.24090 (Δ_m = 0.00058, Δ_m/m = 1.3 ppm).

2-[7-(Diethylamino)-4-(1-hydroxybut-3-yn-1-yl)-*2H*-chromen-2-ylidene]malononitrile (13)

A solution of TBDMS-protected dicyanocoumarin **12** (1.82 g, 4.07 mmol, 1.0 eq) in acetic acid (2.79 mL, 48.8 mmol, 12 eq) and 35 mL dry tetrahydrofuran was cooled to 0 °C in an ice bath. Tetrabutylammonium fluoride (1 M in tetrahydrofuran, 12.2 mL, 12.2 mmol, 3 eq) was added and the mixture was stirred at room temperature. After 72 h, the solvent was removed under reduced pressure and the residue was purified by silica gel column chromatography (DCM/acetone 100:5). Alcohol **13** was obtained as an orange solid.

Yield: 1.22 g (3.66 mmol, 90%).

TLC: R_f = 0.48 (DCM/acetone 9:1).

^{1}H-NMR: (500 MHz, $CDCl_3$): δ (ppm) = 7.46 (d, *J* = 9.1 Hz, 1 H, H-5), 6.94 (s, 1 H, H-3), 6.75 (dd, *J* = 9.1 Hz, 2.3 Hz, 1 H, H-6), 6.65 (d, *J* = 2.4 Hz, 1 H, H-8), 5.18 (dd, *J* = 7.4 Hz, 4.4 Hz, 1 H, C*H*OH), 3.46 (q, *J* = 7.1 Hz, 4 H, N(CH_2Me)$_2$), 2.81 (ddd, *J* = 17.0 Hz, 4.4 Hz, 2.7 Hz, 1 H, CH_2CCH), 2.63 (ddd, *J* = 17.0 Hz, 7.5 Hz, 2.6 Hz, 1 H, CH_2CCH), 2.19 (t, *J* = 2.6 Hz, 1 H, CH_2CC*H*), 1.24 (t, *J* = 7.1 Hz, 6 H, N(CH_2CH_3)$_2$).

^{13}C{^{1}H}-NMR: (126 MHz, $CDCl_3$): δ (ppm) = 172.0 (C-2), 155.2 (C-8a), 152.2 (C-4), 151.0 (C-7), 125.4 (C-5), 114.7 (C*C*N), 114.0 (C*C*N), 111.4 (C-6), 107.7 (C-3), 106.1 (C-4a), 98.5 (C-8), 78.8 (*C*CH), 73.1 (C*C*H), 67.9 (*C*HCH_2CCH), 55.7 (*C*(CN)$_2$), 45.6 (N(*C*$H_2$$CH_3$)$_2$), 28.3 (CH*C*$H_2$CCH), 12.5 (N($CH_2$*C*$H_3$)$_2$).

MALDI-HRMS: *m/z* calculated for $C_{20}H_{20}N_3O_2$ $[M+H]^+$ 334.15501, found 334.15460 (Δ_m = 0.00041, Δ_m/m = 1.2 ppm).

2-Cyanoethyl-{1-[2-(dicyanomethylene)-7-(diethylamino)-*2H*-chromen-4-yl]but-3-yn-1-yl}-*N,N*-diisopropylphosphoramidite (14)

Coumarin alcohol **13** (200 mg, 600 µmol, 1.0 eq) was dissolved in 8 mL dry DCM. DIPEA (0.31 mL, 1.8 mmol, 3.0 eq) was added and the mixture was stirred at room temperature. After 15 min, 2-cyanoethyl *N,N*'-diisopropylchlorophosphoramidite (0.16 mL, 0.72 mmol, 1.2 eq) was added and the mixture was stirred for an additional 19 h. The reaction mixture was washed with conc. $NaHCO_3$ solution, the organic layers were then dried over Na_2SO_4 and the solvent was removed under reduced pressure. Purification by silica gel column chromatography (cyclohexane/ethyl acetate 1:1) gave phosphoramidite **14** as an orange solid.

Yield: 253 mg (474 µmol, 79%).

TLC: R_f = 0.65 (cyclohexane/ethyl acetate 1:1).

^{1}H-NMR: (500 MHz, $CDCl_3$): δ (ppm) = 7.50 (dd, *J* = 9.2 Hz, *J* =1.7 1 H, H-5), 6.90 (s, 1 H, H-3), 6.68–6.44 (m, 1 H, H-6), 6.60 (dd, 1 H, H-8), 5.28–5.18 (m, 1 H, C*H*CH_2CCH), 4.00–3.58 (m, 4 H, PN(C*H*(*i*Pr)$_2$)$_2$, OC$H_2$$CH_2$CN), 3.44 (q, *J* = 7.0 Hz, N(CH_2Me)$_2$), 2.76–2.68 (m, 3 H, CHCH_2CCH, OCH_2CH_2CN), 2.56 (t, *J* = 6.1 Hz, 1 H, OCH_2CH_2CN), 2.06 (dt, *J* = 8.1 Hz, 2.5 Hz, CH_2CC*H*), 1.28–1.11 (m, 18 H, PN(CH(CH_3)$_2$)$_2$, N(CH_2CH_3)$_2$).

^{1}H-NMR: (500 MHz, DMSO-d_6): δ (ppm) = 7.74 (dd, *J* = 9.3 Hz, 3.9 Hz, 1 H, H-5), 6.87–6.83 (m, 2 H, H-3, H-6), 6.67 (dd, *J* = 5.4 Hz, 2.5 Hz, H-8), 5.46–5.38 (m, 1 H, C*H*CH_2CCH), 3.93–3.56 (m, 4 H, PN(C*H*(*i*Pr)$_2$)$_2$, OC$H_2$$CH_2$CN), 3.50 (q, *J* = 7.0 Hz, N(CH_2Me)$_2$), 2.94–2.92 (m,

1 H, CH_2CCH), 2.88–2.64 (m, 2 H, $CHCH_2CCH$), 2.85 (t, J = 5.9 Hz, 1 H, OCH_2CH_2CN), 2.67 (t, J = 5.9 Hz, 1 H, OCH_2CH_2CN), 1.24–1.04 (m, 18 H, $PN(CH(CH_3)_2)_2$, $N(CH_2CH_3)_2$).

$^{13}C\{^1H\}$-NMR: (126 MHz, $CDCl_3$): δ (ppm) = 172.0 (C-2), 171.9 (C-2), 155.5 (C-8a), 155.4 (C-8a), 151.9 (C-4), 151.7 (C-7), 151.6 (C-7), 125.6 (C-5), 117.8 (OCH_2CH_2CN), 117.7 (OCH_2CH_2CN), 114.9 ($C(CN)_2$), 114.7 ($C(CN)_2$), 114.2 ($C(CN)_2$), 110.6 (C-6), 107.1 (C-3), 106.8 (C-4a), 106.7 (C-4a), 97.7 (C-8), 78.9 (*C*CH), 72.3 (C*C*H), 70.2 ($CHCH_2CCH$), 70.1 ($CHCH_2CCH$), 69.7 ($CHCH_2CCH$), 69.6 ($CHCH_2CCH$), 58.7 (OCH_2CH_2CN), 58.5 (OCH_2CH_2CN), 58.3 (OCH_2CH_2CN), 55.3 ($C(CN)_2$), 55.2 ($C(CN)_2$), 45.0 ($N(CH_2CH_3)_2$), 43.7 ($PN(CH(CH_3)_2)_2$), 43.6 ($PN(CH(CH_3)_2)_2$), 43.5 ($PN(CH(CH_3)_2)_2$), 28.0 ($CHCH_2CCH$), 27.8 ($CHCH_2CCH$), 24.8 ($PN(CH(CH_3)_2)_2$), 24.7 ($PN(CH(CH_3)_2)_2$), 20.6 (OCH_2CH_2CN), 20.5 (OCH_2CH_2CN), 12.6 ($N(CH_2CH_3)_2$).

$^{13}C\{^1H\}$-NMR: (126 MHz, DMSO-d_6): δ (ppm) = 171.3 (C-2), 171.1 (C-2), 154.9 (C-8a), 154.8 (C-8a), 153.0 (C-4), 152.9 (C-4), 151.6 (C-7), 151.4 (C-7), 126.3 (C-5), 119.0 (OCH_2CH_2CN), 118.6 (OCH_2CH_2CN), 114.9 ($C(CN)_2$), 114.7 ($C(CN)_2$), 114.1 ($C(CN)_2$), 114.0 ($C(CN)_2$), 111.2 (C-6), 111.1 (C-6), 106.4 (C-3), 106.3 (C-3), 105.4 (C-4a), 105.1 (C-4a), 96.5 (C-8), 79.2 (*C*CH), 74.4 (C*C*H), 74.3 (C*C*H), 69.2 ($CHCH_2CCH$), 69.0 ($CHCH_2CCH$), 68.6 ($CHCH_2CCH$), 68.5 ($CHCH_2CCH$), 58.8 (OCH_2CH_2CN), 58.6 (OCH_2CH_2CN), 58.4 (OCH_2CH_2CN), 58.2 (OCH_2CH_2CN), 52.2 ($C(CN)_2$), 44.2 ($N(CH_2CH_3)_2$), 43.0 ($PN(CH(CH_3)_2)_2$), 42.9 ($PN(CH(CH_3)_2)_2$), 42.8 ($PN(CH(CH_3)_2)_2$), 27.2 ($CHCH_2CCH$), 24.4 ($PN(CH(CH_3)_2)_2$), 24.3 ($PN(CH(CH_3)_2)_2$), 24.2 ($PN(CH(CH_3)_2)_2$), 19.9 (OCH_2CH_2CN), 19.8 (OCH_2CH_2CN), 19.7 (OCH_2CH_2CN), 12.3 ($N(CH_2CH_3)_2$).

$^{31}P\{^1H\}$-NMR: (202 MHz, $CDCl_3$): δ (ppm) = 149.9 (s), 149.9 (s).

$^{31}P\{^1H\}$-NMR: (202 MHz, DMSO-d_6): δ (ppm) = 148.9 (s), 148.8 (s).

MALDI-HRMS: *m/z* calculated for $C_{29}H_{37}N_5O_3P$ $[M+H]^+$ 534.26286, found 534.26249 (Δ_m = 0.00037, Δ_m/m = 0.7 ppm).

2 Oligonucleotide Synthesis

RNase-free water was used for all works involving oligonucleotides. Therefore, Milli-Q water containing 0.1% diethyl pyrocarbonate (DEPC) was stirred overnight and autoclaved before usage.

Oligonucleotides were synthesized on an ABI 392 DNA/RNA synthesizer using DMTon strategy at 1 µmol scales. 0.3 M BTT in acetonitrile (emp Biotech) was used as activator together with UltraMild capping reagents (tetrahydrofuran/pyridine/phenoxyacetic anhydride, emp Biotech). The coupling time for BODIPY and coumarin phosphoramidites was extended to 12 min. DNA phosphoramidites were coupled within 30 s. 3% TCA in DCM (emp Biotech) was used for detritylation and *Oxidizing (ABI)* (J.T.Baker) was used for oxidation.

For **ON1**, CPG support containing a serinol alkyne modifier (*3'-Alkyne-Modifier Serinol CPG*, 1000 Å, Glen Research) was used. **ON2** and **ON4** were synthesized on *Alkin-Modifier CPG 500* (500 Å, Lumiprobe). Both modifiers are illustrated in Figure S2. A standard dT CPG solid support (500 Å, Glen Research) was used for the synthesis of **ON3**.

DNA amidites were bought from Linktech. Amidites were used at 0.1 M concentration in ACN except for BODIPY phosphoramidite **5** and coumarin phosphoramidite **14** that were used at higher concentrations of 0.12 M. Due to insufficient solubility of BODIPY phosphoramidite **5** in acetonitrile, a mixture of DCM/MeCN 1:1 was used.

Cleavage and deprotection of **ON1**, **ON2** and **ON3** was carried out using 0.05 M K_2CO_3 in methanol. A desalting step was performed after deprotection (*illustra NAP columns*, GE Healthcare). RNase-free water was used for elution. The crude products were concentrated under reduced pressure in a vacuum centrifuge and then purified by RP-HPLC.

In the case of **ON1**, the two diastereomers showed baseline separation. Further experiments were performed with one pure stereoisomer.

ON4 was incubated with 30% aqueous NH_3 solution overnight at room temperature for cleavage. After filtration, the solvent was evaporated in a vacuum concentrator. The residue was dissolved in water and purified by RP-HPLC. The DMT protecting group at the 5' terminus was then removed in 80% acetic acid within 20 min. After vacuum concentration, **ON4** was again purified by RP-HPLC.

Table S1: Overview of chemically synthesized oligonucleotides for this work.

	**Sequence (5'→ 3')**
**ON1**	**5** TTT TTT TT **a**
**ON2**	**14** TTT TTT TT **b**
**ON3**	**14** TTT TTT TT
**ON4**	TTT TTT TTT TTT TTT T **b**

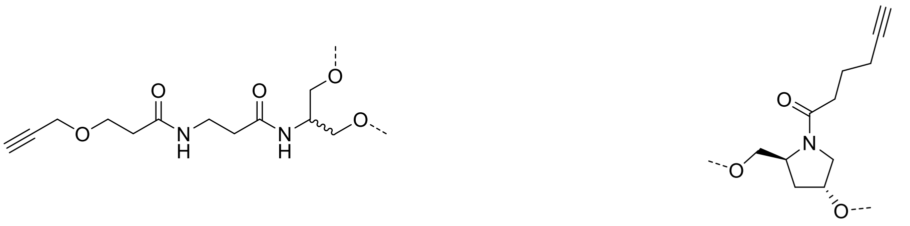

Serinol Alkyne Modifier **a**
(*3'-Alkyne-Modifier Serinol CPG*, Glen Research)

Hydroxyprolinol Alkyne Modifier **b**
(Alkin-Modifier CPG 500, Lumiprobe)

Figure S2: Alkyne modifiers used in solid-phase synthesis.

RP-HPLC Purification

Agilent 1200 and Agilent 1260 Infinity systems equipped with Waters XBridge columns were used for RP-HPLC purification and analysis. 400 mM hexafluoroisopropanol (HFIP), 16.3 mM TEA, pH 7.9 was used as a buffer system. The buffer was used against a methanol gradient to elute the oligonucleotide. If not stated otherwise, purification and analysis was performed at a temperature of 25 °C.

Table S2: Columns used in this work for RP-HPLC purification. All columns were purchased from Waters.

Column	Name
1	XBridge Peptide BEH C18 OBD Prep Column, 300 Å, 5 µm, 10 x 250 mm
2	XBridge BEH C18 OBD Prep Column, 130 Å, 5 µm, 10 x 50 mm
3	XBridge Peptide BEH C18 Column, 300 Å, 3.5 µm, 4.6 x 250 mm

*Table S3: Gradients used for RP-HPLC purification of the synthesized oligonucleotides **ON1-ON5**.*

	Column	Gradient
**ON1**	1	0–1 min 5–30% MeOH, 1–8 min 30–50% MeOH, 8–10 min 50–100% MeOH
**ON2**	2	0–3 min 5% MeOH, 3–12 min 5–60% MeOH, 12–15 min, 60–100% MeOH
**ON3**	2	0–2 min 5% MeOH, 2–14 min 5–60% MeOH, 14–16 min 60–100% MeOH
**ON4**	2	0–2 min 5% MeOH, 2–4 min 5–20% MeOH, 4–20 min 20–60% MeOH, 20–24 min 60–100% MeOH; 40 °C
**ON5**	3	0–2 min 5% MeOH, 2–4 min 5–20% MeOH, 4–29 min 20–70% MeOH, 29–31 min 70–100% MeOH

Mass Spectrometry

Purity and identity of all oligonucleotides was confirmed by LC-MS (LC: Agilent 1200 system equipped with Waters XBridge Peptide BEH C18 column (300 Å, 3.5 µm, 2.1 mm x 250 mm), buffer: 400 mM HFIP, 16.3 mM TEA, pH 7.9 / MeOH; MS: Bruker micrOTOF-QII ESI).

Polyacrylamide Gel Electrophoresis (PAGE)

Oligonucleotides were analyzed by polyacrylamide gel electrophoresis using a 20% polyacrylamide solution under denaturing conditions. Electrophoresis was performed at 240 V for 35 min. The used DNA ladder contained ssDNAs of different lengths (10mer, 15mer, 20mer, 25mer, 30mer, 35mer poly-dT) purchased from Biomers. SYBR Gold (ThermoFisher) was used for staining and photos were taken with a Bio-Rad Laboratories Gel Doc XR+.

3 Photochemical Experiments

3.1 Absorption and Fluorescence Spectra

Absorption spectra of compounds **4**, **13**, and **ON3** were taken on a Jasco V-650 UV-vis spectrophotometer using a 10.00 mm path length quartz glass cuvette (Hellma Analytics). Fluorescence spectra were recorded on a Hitachi F-4500 fluorometer using the same cuvette. For absorption spectra, OD values close to 1 were chosen. Fluorescence spectra were recorded at an OD between 0.1 - 0.15. Absorption and fluorescence spectra of **ON1** were taken on a Tecan Infinite M200 Pro plate reader.

For extinction coefficient determination, five data points at different concentrations were taken (Jasco V-650 UV-vis spectrophotometer). BODIPY alcohol **4** was measured in a 10.00 mm path length quartz glass cuvette and a 2.00 mm path length quartz glass cuvette (both Hellma Analytics) was used for coumarin alcohol **13**. All spectra were baseline corrected by subtraction of the absorbance minimum in a region where the compound does not absorb light (700–800 nm) from all data points. Absorbance was plotted against concentration and an interception at the origin of the graph was set for linear fits. Extinction coefficients were then calculated using the Beer-Lambert law.

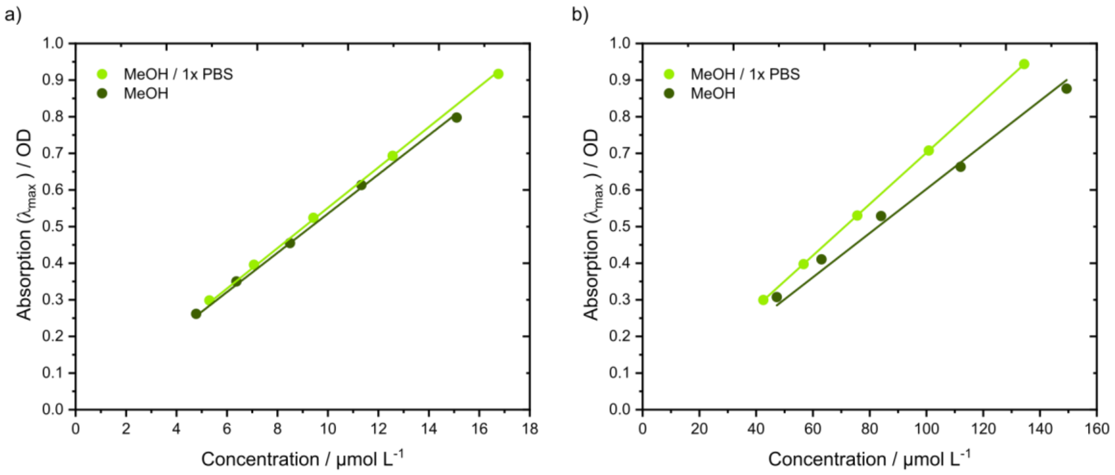

*Figure S3: Determination of extinction coefficient at absorption maxima in MeOH and MeOH/1x PBS (1:1) for a) BODIPY alcohol **4** (10 mm path length cuvette) and b) coumarin alcohol **13** (2 mm path length cuvette).*

3.2 Actinometry and Photolysis Quantum Yield

A 530 nm LED (M530L3, Thorlabs) was used for the determination of quantum yields. A concentrated solution of the indolyl fulgide photoswitch in toluene was switched from its closed form to the *Z*-form while the change in its absorption spectrum was tracked (Ocean Optics DH mini light source, Ocean Optics USB4000 or Thorlabs CCS200/M detector). Photon flux was then determined following our recently published fulgide actinometry method.[4] More information on the setup can also be found there. Photon flux for the irradiation of **ON1** was 9.52 nmol/s and 22.32 nmol/s for **ON3**.

Next, solutions of **ON1** (6 µM) and **ON3** (20 µM) in 1x PBS containing uridine as an internal standard were prepared. 50 µL aliquots of these solutions were irradiated in three series with multiple time points (**ON1**: 0 min, 5 min, 10 min, 20 min, 30 min, 60 min, 90 min; **ON3**: 0 s, 10 s, 20 s, 30 s, 60 s, 90 s, 120 s, 240 s) using the same setup as for photon flux determination. Each sample was then analyzed by RP-HPLC (Waters XBridge Peptide BEH C18 Column, 300 Å, 3.5 µm, 4.6 x 250 mm) and each run was referenced to the internal standard. Starting material consumption was then determined by peak integration. Exponential fitting of the starting material consumption plotted against the irradiation time gave the initial slope at t = 0. Quantum yields were then calculated as the ratio between the initial slope and the absorbed photon flux.

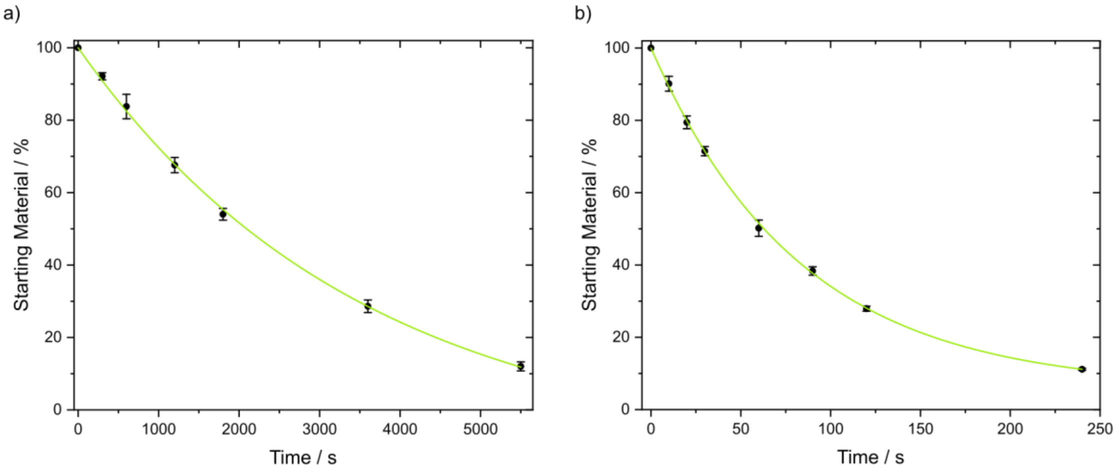

Figure S4: Photolysis curves of a) ***ON1*** *and b)* ***ON3****. Starting material consumption was calculated by integration of HPLC peak areas.*

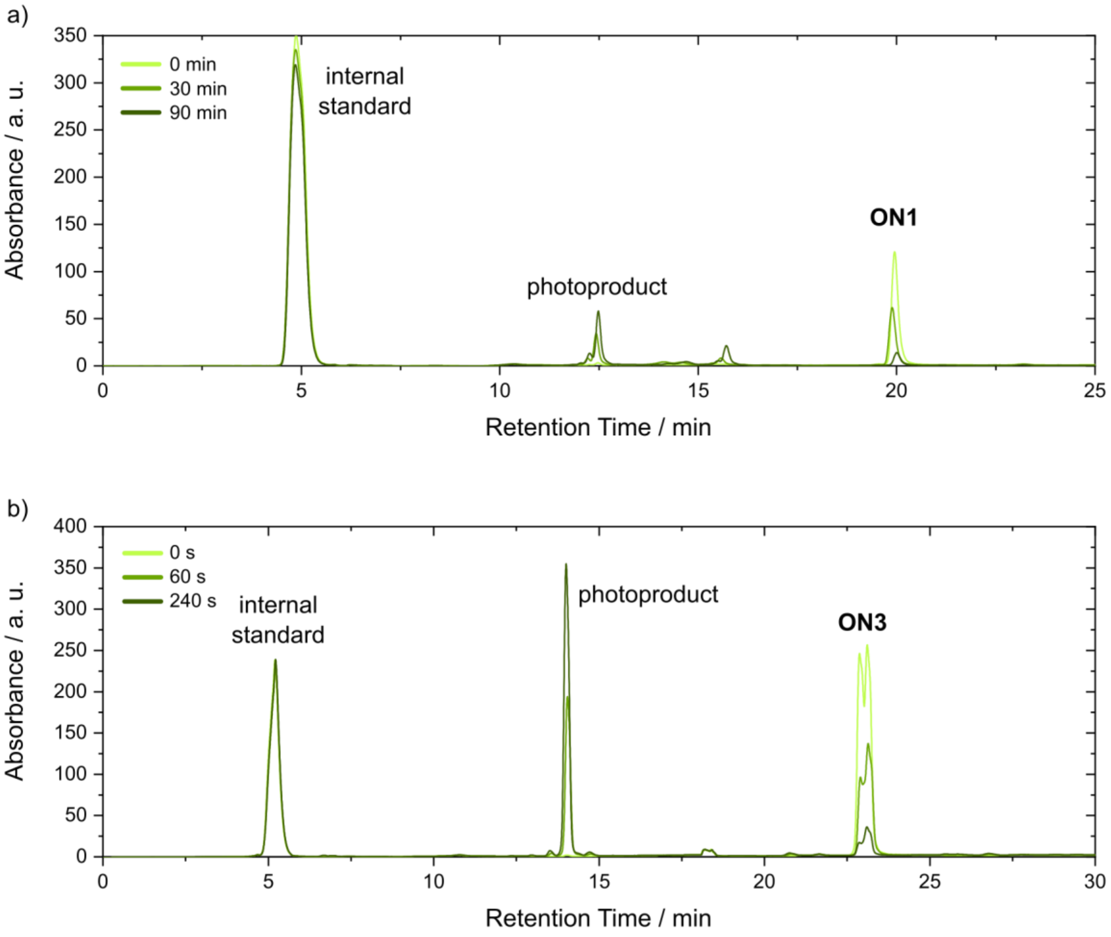

Figure S5: Exemplary traces for uncaging quantum yield determination of a) ***ON1*** *(gradient: 25–50% MeOH in 22 min) and b)* ***ON3*** *(gradient: 20–60% MeOH in 23 min).*

3.3 Uncaging Experiments

Uncaging Tests at 565 nm

Uncaging of the synthesized cages with a 565 nm LED (M565L3, Thorlabs) was tested with **ON1** and **ON2**. A solution of the respective oligonucleotide in 1x PBS (**ON1**: 400 µL, 5 µM; **ON2**: 600 µL, 5 µM) was placed under the focal area of the light source and irradiated (**ON1**: 100 mW, 10 min; **ON2**: 15 mW, 30 min). The solution was then analyzed by RP-HPLC (Waters XBridge Peptide BEH C18 Column, 300 Å, 3.5 µm, 4.6 x 250 mm) and retention times were compared to those of non-irradiated samples. Molecular masses of the photolysis products were confirmed by ESI-MS.

*Table S4: ESI-MS data for the characterized photoproducts of **ON1** and **ON2**.*

	Calculated Mass	Found Mass
Photoproduct of **ON1**	2783.4652 Da	2784.8052 Da
Photoproduct of **ON2**	2722.4487 Da	2723.8176 Da

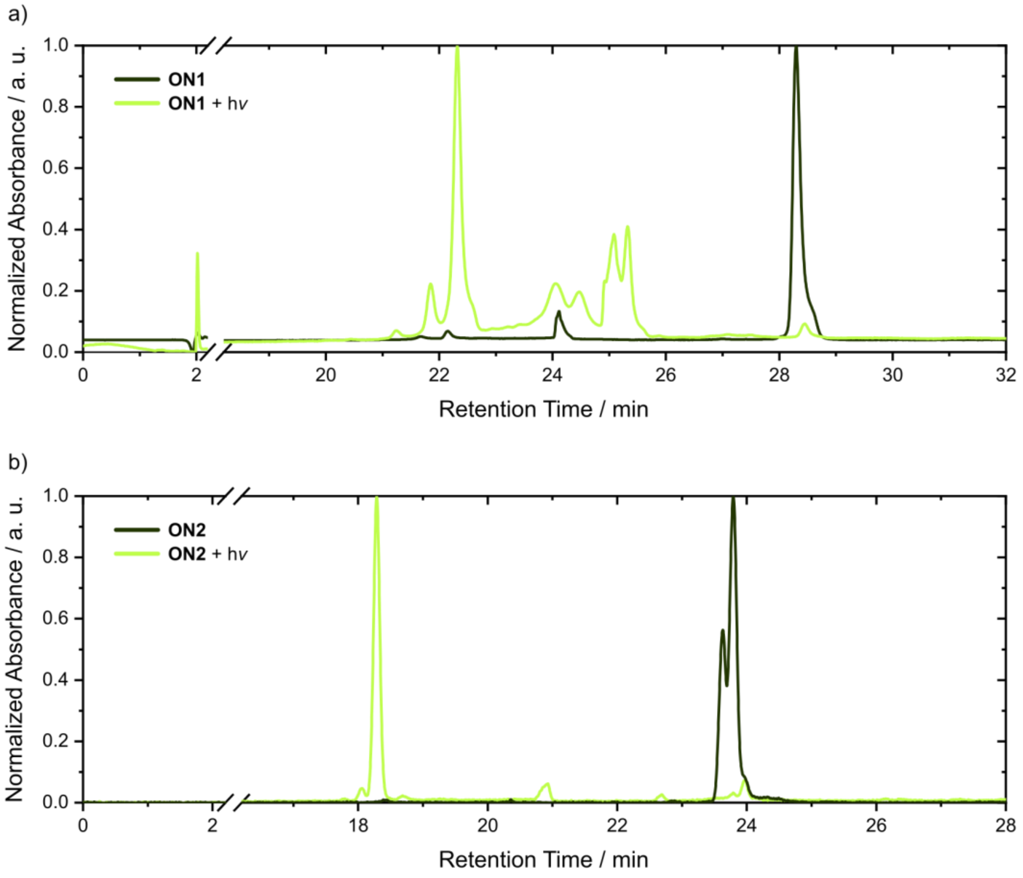

*Figure S6: Photolysis experiments with a) **ON1** (gradient: 5–60% MeOH in 22 min) and b) **ON2** (gradient: 5–80% MeOH in 30 min). A 565 nm LED was used in both cases for uncaging (**ON1**: 100 mW, 10 min; **ON2**: 15 mW, 30 min).*

Photolysis Comparison ON1 vs. ON2

BODIPY-modified **ON1** and coumarin-modified **ON2** were irradiated simultaneously under a 530 nm LED (M530L3, Thorlabs). The lamp was placed over a cover glass that had 3 wells glued to it. The focal area was then set to be wide enough to cover all wells. 13 µL of 1x PBS-buffered **ON1** and **ON2** stock solutions (83 µM each) containing uridine as an internal standard were pipetted in two adjacent wells and the lamp was switched on for a defined time period (27 mW). This procedure was repeated so that 10 samples per oligonucleotide with different irradiation times (0 min, 1 min, 2 min, 5 min, 10 min, 15 min, 20 min, 30 min, 60 min) were obtained. All samples were analyzed by RP-HPLC (Waters XBridge Peptide BEH C18 Column, 300 Å, 3.5 µm, 4.6 x 250 mm, gradient: 5–80% MeOH in 30 min) and the species were identified by ESI-MS. The setup is shown in Figure S7.

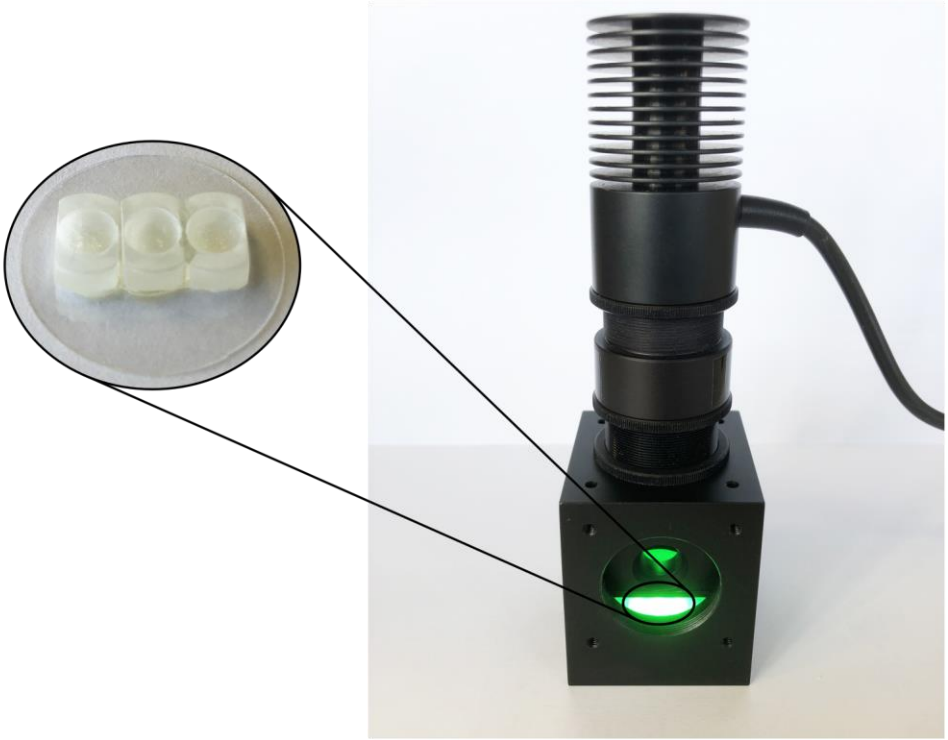

*Figure S7: Setup for the comparison of photolysis rates of **ON1** and **ON2**. The wells are glued to a cover glass and all placed within the focal area of the LED.*

Photocleavage of Photo-Tethered cON1/cON2 and Proof for Cyclization

Solutions of photo-tethered **cON1** (300 µL, 5 µM) or **cON2** (200 µL, 5 µM) were placed in the focal area of a 530 nm LED (M530L3, Thorlabs) and irradiated with green light (**cON1**: 10 min, 1000 mA; **cON2**: 20 min, 14.8 mW). The resulting solution was analyzed by RP-HPLC (Waters

XBridge Peptide BEH C18 Column, 300 Å, 3.5 µm, 4.6 x 250 mm) and retention times were compared to the respective linear and non-irradiated samples. Uncaging and therefore relinearization of **cON1**/**cON2** was confirmed by ESI-MS. Due to the 17 Da mass increase after uncaging, the method served as proof for successful cyclization.

Table S5: ESI-MS data for the characterized photoproducts of ***cON1*** *and* ***cON2****.*

	**Calculated Mass**	**Found Mass**
Photoproduct of **cON1**	3288.6475 Da	3287.2 Da
Photoproduct of **cON2**	3244.6230 Da	3245.1093 Da

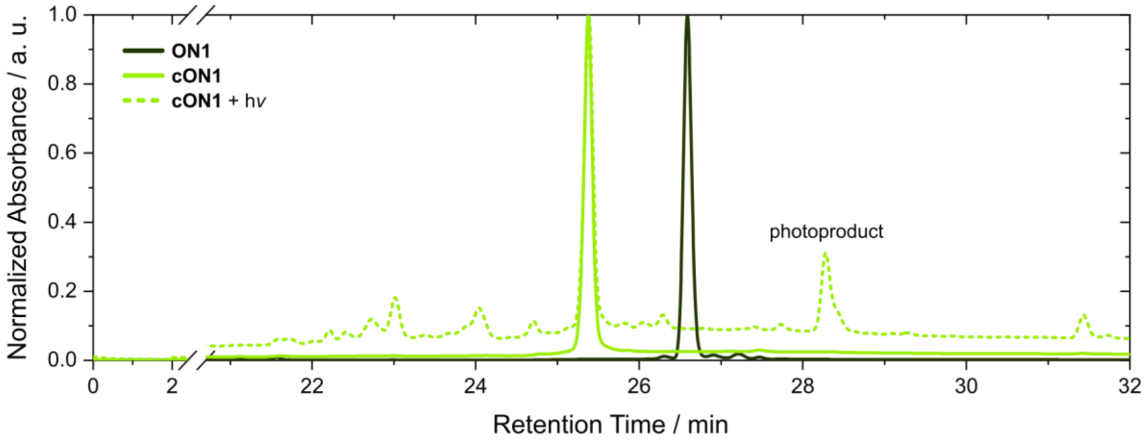

Figure S8: Retention times of linear ***ON1****, photo-tethered* ***cON1*** *and* ***cON1*** *after irradiation for 10 min (530 nm LED, 1000 mA) (internal uridine standard not shown, gradient: 5–60% MeOH in 22 min).*

Strand Break Induction in ON5

A 530 nm LED (90 mW, M530L3, Thorlabs) was focused on a reaction tube containing a 1x PBS-buffered solution of **ON5** (100 µL, 20 µM) with uridine as an internal standard. The sample was irradiated for 20 min and an aliquot was purified by RP-HPLC (Waters XBridge Peptide BEH C18 Column, 300 Å, 3.5 µm, 4.6 x 250 mm, gradient: 5–80% MeOH in 30 min). Both photoproducts isolated by RP-HPLC and identified by ESI-MS were then applied to a polyacrylamide gel as a reference together with another aliquot of the irradiated solution.

Table S6: ESI-MS data for the characterized strand break photoproducts of ***ON5****.*

	**Calculated Mass**	**Found Mass**
Photoproduct 1 (8mer + PO_4^{3-})	2449.3711 Da	2450.6755 Da
Photoproduct 2 (16mer + AMB + coumarin)	5597.0245 Da	5597.7395 Da

4 CuAAC Click Reactions

General Procedure for CuAAC Click Reactions

CuAAC reactions were performed at 5-10 nmol scales. Cu(I) solutions were freshly prepared and used directly. All other reagents were used from stock solutions stored at −21 °C. Stock concentrations, as well as final concentrations in the reaction mixture for the photo-tethering reactions and the chemical ligation reactions, are given in the tables below.

In both cases, water was pipetted to the dry oligonucleotide (vacuum concentrator) and AMB linker solution was added in the next step. A CuI solution was prepared freshly by addition of DMSO to CuI and vortexed for 2 seconds. The remaining solid was centrifuged and the supernatant was used to prepare a mixture of CuI and Tris[(1-benzyl-1*H*-1,2,3-triazol-4-yl)methyl]amin (TBTA) (1:2 ratio). The CuI-TBTA solution was then added to the reaction and the reaction mixture was filled up with DMSO to reach an oligonucleotide concentration of 769 µM. After 2 h at 45 °C, the reaction was stopped by addition of HPLC buffer (100 µL total volume) and then purified by RP-HPLC (Waters XBridge Peptide BEH C18 Column, 300 Å, 3.5 µm, 4.6 x 250 mm).

Photo-Tethering Reactions

Photo-tethering reactions were carried out following the general procedure given above. **cON1** was purified by HPLC using a 5–60% MeOH in 33 min gradient and **cON2** was purified using a 15–50% MeOH in 40 min gradient.

Table S7: Concentrations and solvents for CuAAC reagents used for photo-tethering reactions. An example for a CuAAC photo-tethering reaction at a 10 nmol scale is given.

	**c(stock)**	**Solvent**	**c(final)**	**At 10 nmol scale:**
**Oligonucleotide**	2 mM	H_2O	769 µM	5.00 µL
**AMB linker**	10 mM	H_2O/DMSO/*t*BuOH 4:3:1	769 µM	1.000 µL
**TBTA**	100 mM	DMSO/*t*BuOH 3:1	15.4 mM	3.00 µL (as pre-mixed CuI-TBTA solution)
**CuI**	100 mM	DMSO	7.69 mM	
			add DMSO:	4.00 µL
			**total volume:**	**13.00 µL**

Chemical CuAAC Click Ligation

Chemical ligation reactions involved two consecutive steps. The first step was the 3' elongation of **ON4** through addition of the AMB linker. In the next step, **ON3** was attached in a second CuAAC reaction.

Step 1: AMB conjugation

The first step was performed as described in the general procedure but needed more equivalents of AMB linker than the photo-tethering reaction. Also, the final reaction volume was increased to prevent dimerization. A 20–60% MeOH in 16 min HPLC gradient was used for purification.

Table S8: Concentrations and solvents for CuAAC reagents used for AMB-conjugation as the first step of chemical ligation. An example of an AMB-conjugation reaction at a 10 nmol scale is given.

	c(stock)	Solvent	c(final)	At 10 nmol scale:
**Oligonucleotide**	2 mM	H_2O	385 µM	5.00 µL
**AMB linker**	10 mM	H_2O/DMSO/*t*BuOH 4:3:1	1.92 mM	5.00 µL
**TBTA**	100 mM	DMSO/*t*BuOH 3:1	7.69 mM	3.00 µL (as pre-mixed CuI-TBTA solution)
**CuI**	100 mM	DMSO	3.84 mM	
			add H_2O:	6.50 µL
			add DMSO:	6.50 µL
			**total volume:**	**26.00 µL**

Step 2: CuAAC ligation

For the second step, equal amounts of each oligonucleotide (still 5–10 nmol scale) were combined and dried in a vacuum concentrator. The original volume of AMB solution was replaced by DMSO. The crude product was purified using a 20–70% MeOH in 25 min HPLC gradient.

Table S9: Concentrations and solvents for CuAAC reagents used for CuAAC ligation as the second step of chemical ligation. An example of a CuAAC ligation reaction at a 10 nmol scale is given.

	c(stock)	Solvent	c(final)	At 10 nmol scale:
**AMB-conjugated Oligonucleotide A**	2 mM	H_2O	769 µM	5.00 µL
**Oligonucleotide B**	2 mM	H_2O	769 µM	
**TBTA**	100 mM	DMSO/*t*BuOH 3:1	15.4 mM	3.00 µL (as pre-mixed CuI-TBTA solution)
**CuI**	100 mM	DMSO	7.69 mM	
			add DMSO:	5.00 µL
			**total volume:**	**13.00 µL**

5 Exonuclease VII Stability Assay

Exonuclease VII used for the stability assay was purchased from NEB (*M0379*, 10,000 U/mL). The poly-dT 20mer ssDNA used as a control was purchased from Biomers.

200 pmol of the respective oligonucleotide were diluted with water to a volume of 15 µL. 4 µL 5x Exo VII Reaction Buffer (supplied with exonuclease) were added and the solution was gently mixed. Last, 1 µL (5 U) of exonuclease VII was added and the mixture was again gently mixed before placing it in a thermo shaker at 37 °C and 200 U/min. After each time point, an aliquot of 2 µL was taken from the mixture, pipetted into 8 µL of denaturing loading buffer and heated to 95 °C for 5 min to destroy any enzyme activity. The aliquots were then stored at −21 °C until they were analyzed by PAGE.

As a control, each oligonucleotide was incubated in 1x PBS (20 pmol, 10 µM) at 37 °C for 24 h.

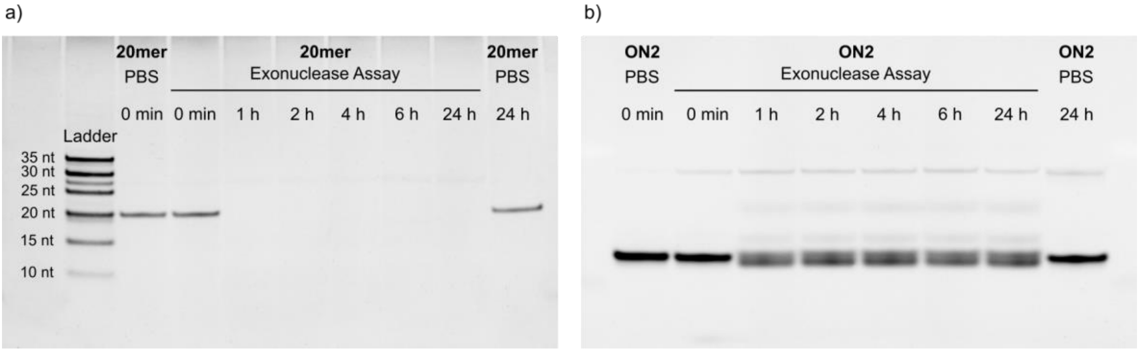

Figure S9: 20% denaturing polyacrylamide gel showing oligonucleotide resistance against exonuclease VII. The respective oligonucleotide was applied as a reference in 1x PBS. a) Exonuclease VII assay with poly-dT 20mer. b) Exonuclease VII assay with linear ***ON2****.*

To confirm the presence of an alkyne-alkyne coupled Glaser side product in the **cON2** sample, the formation of Glaser product was triggered by incubation of **ON2** under CuAAC conditions. AMB solution in this case was replaced by water and the reaction time was extended to 15 h. The resulting RP-HPLC chromatogram (Waters XBridge Peptide BEH C18 Column, 300 Å, 3.5 µm, 4.6 x 250 mm, gradient: 20–60% MeOH in 23 min) of the reaction mixture is shown in Figure S10b. A sharp peak with the same retention time as **cON2** was isolated and analyzed by PAGE. The formation of Glaser product was confirmed by ESI-MS. Irradiation of **cON2** and the Glaser product was performed with 4 µM 1x PBS solutions under a 530 nm LED (50 mW, 10 min). Both oligonucleotides could be relinearized and the photoproducts were identified by ESI-MS.

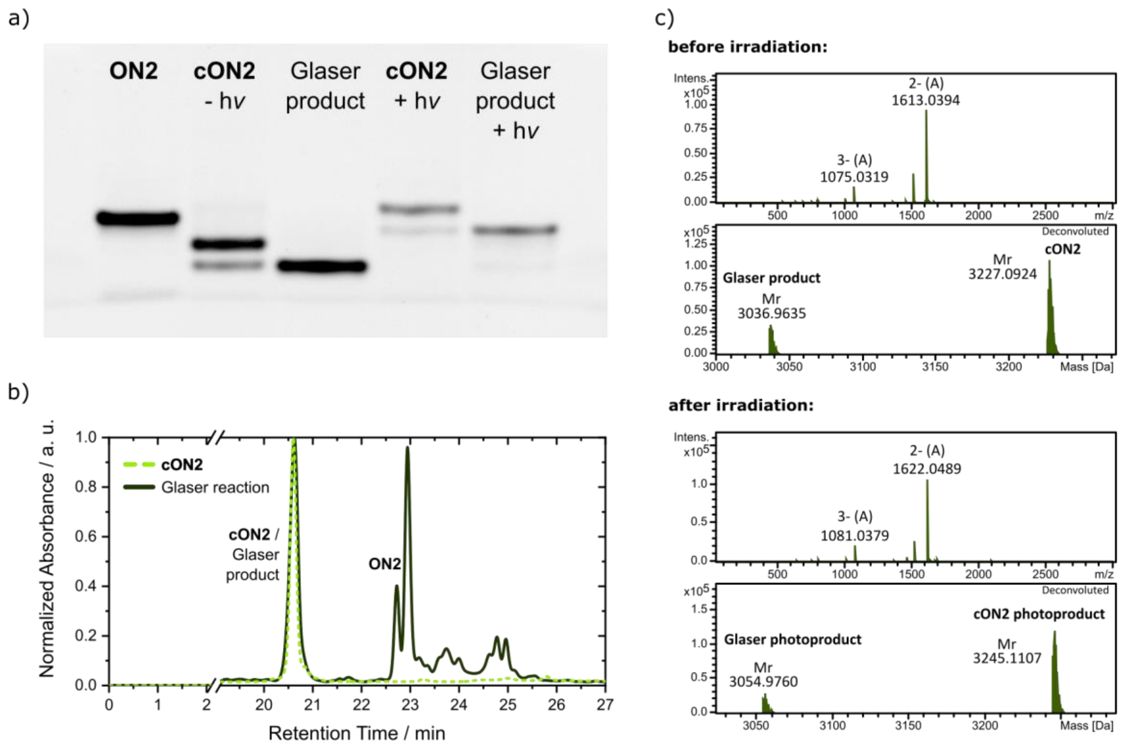

Figure S10: a) 20% denaturing gel of ***ON2***, ***cON2,*** *and the Glaser product as well as irradiated samples of both cyclic oligonucleotides. b) RP-HPLC chromatogram of the Glaser reaction compared to* ***cON2***. *c) ESI-MS data of the* ***cON2*** *sample used in the exonuclease VII stability assay before and after irradiation with green light.*

6 NMR Spectra

6.1 BODIPY NMR Spectra

8-Acetoxymethyl-4,4’-difluoro-1,3,5,7-tetramethyl-4-bora-3a,4a-diaza-*s*-indacene (1)

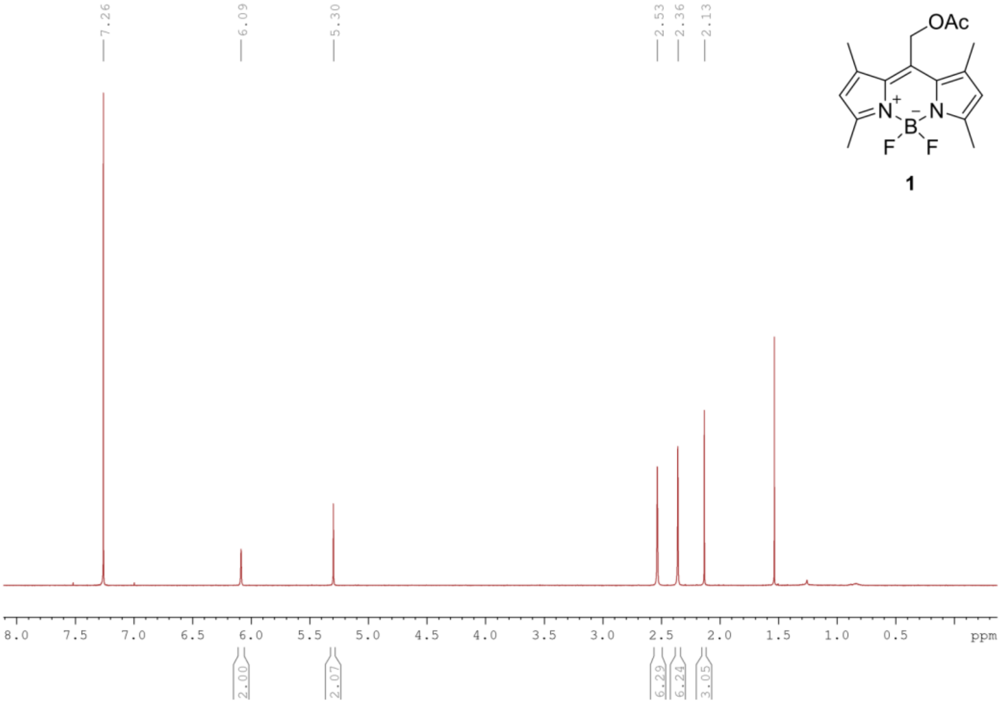

4,4’-Difluoro-8-hydroxymethyl-1,3,5,7-tetramethyl-4-bora-3a,4a-diaza-*s*-indacene (2)

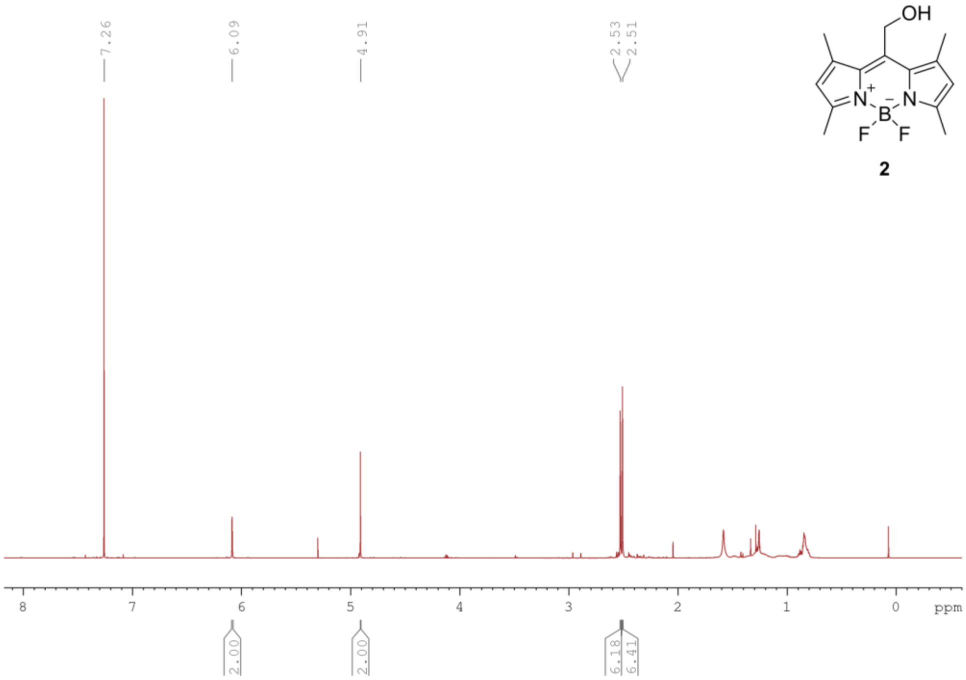

4,4’-Difluoro-8-formyl-1,3,5,7-tetramethyl-4-bora-3a,4a-diaza-*s*-indacene (3)

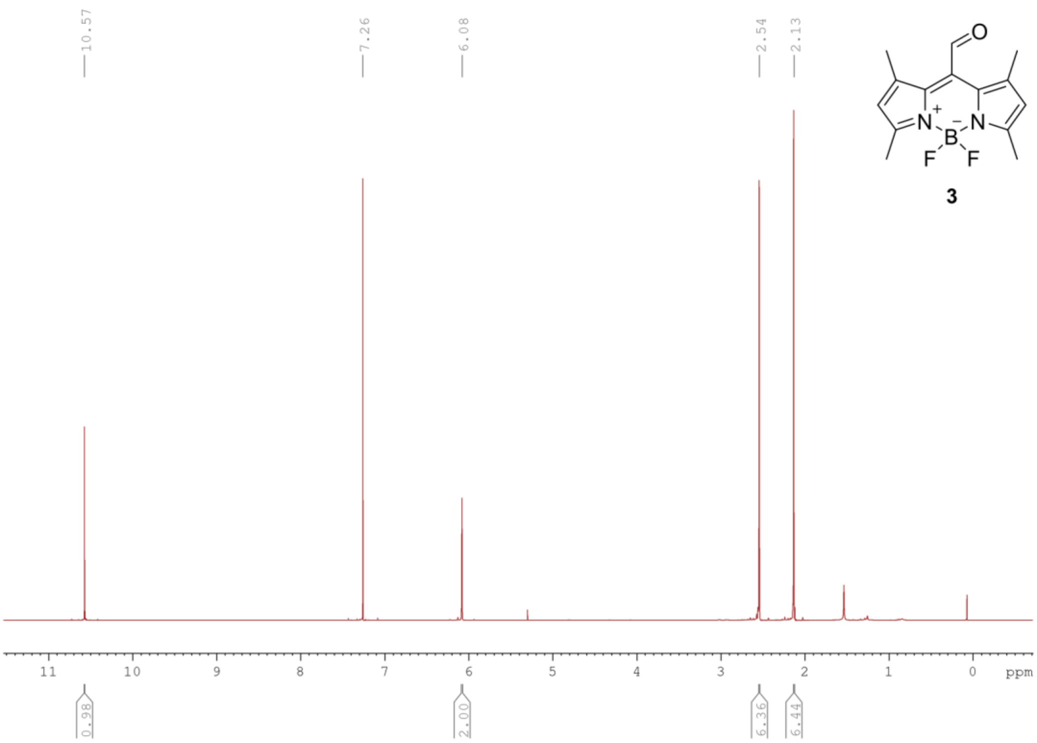

4,4’-Difluoro-8-(1-hydroxybut-3-in-1-yl)-1,3,5,7-tetramethyl-4-bora-3a,4a-diaza-*s*-indacene (4)

^1H ($CDCl_3$):

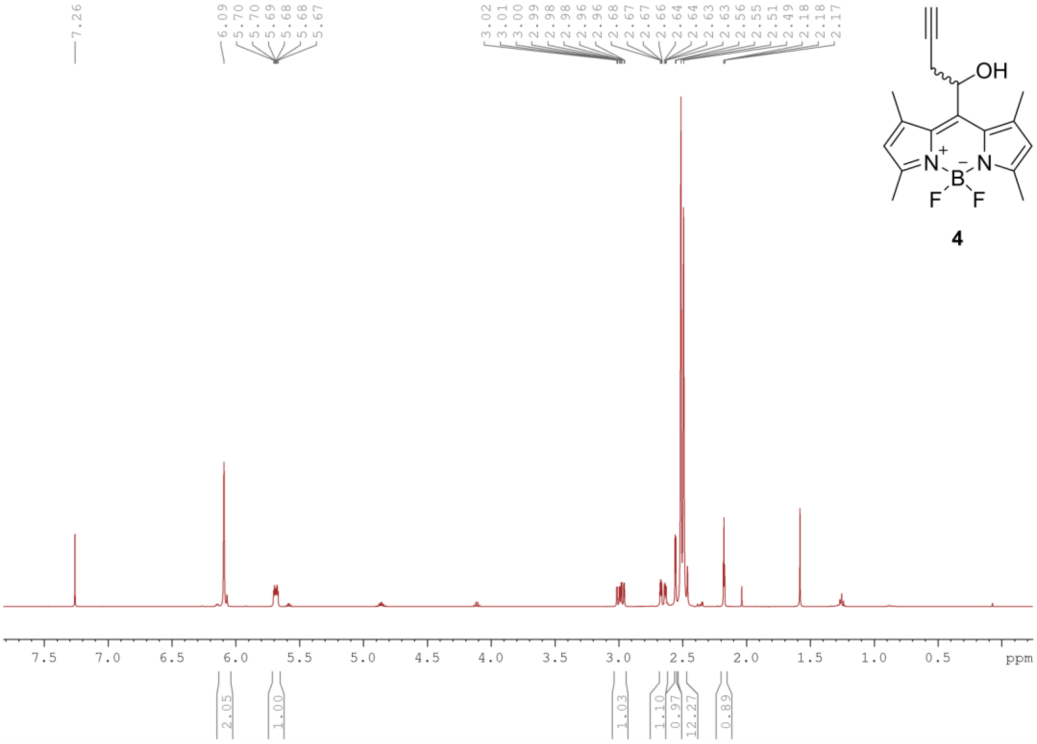

[13]C{[1]H} (CDCl$_3$):

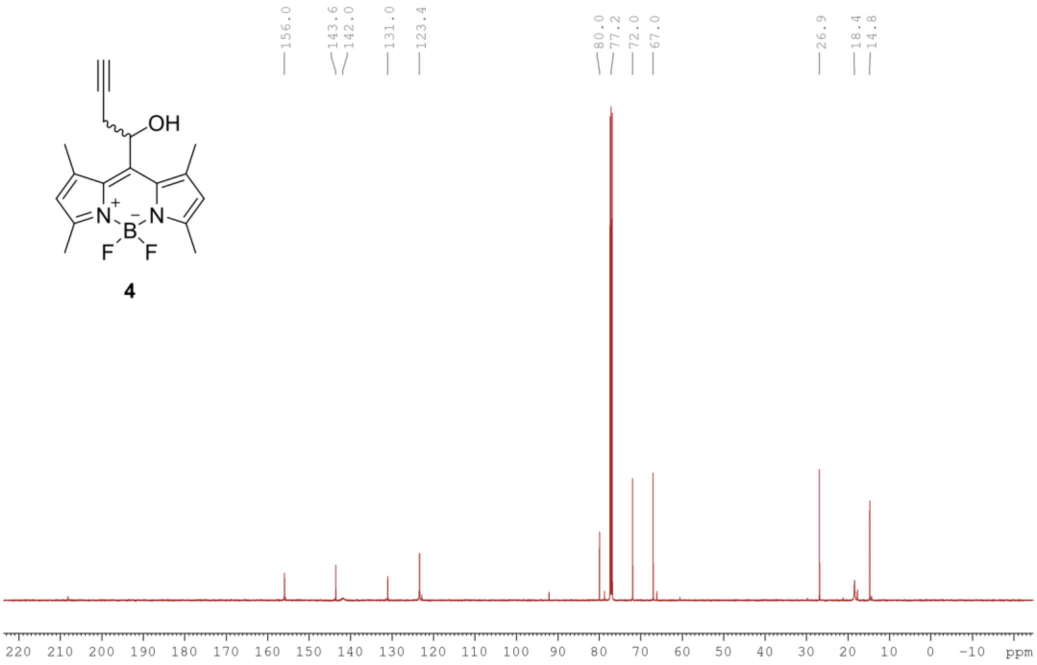

2-Cyanoethyl-[1-(4,4'-difluoro-1,3,5,7-tetramethyl-4-bora-3a,4a-diaza-*s*-indacene-8-yl)but-3-yn-1-yl]-*N*,*N*-diisopropylphosphoramidite (5)

[1]H (CDCl$_3$):

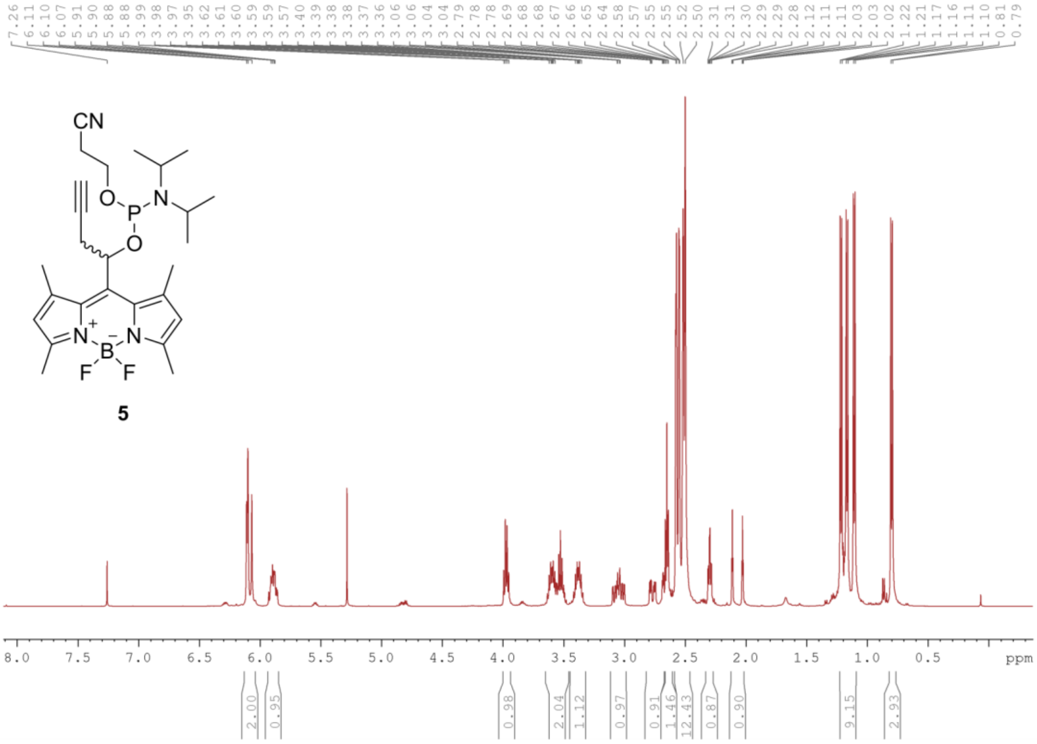

$^{13}C\{^{1}H\}$ *(CDCl$_3$):*

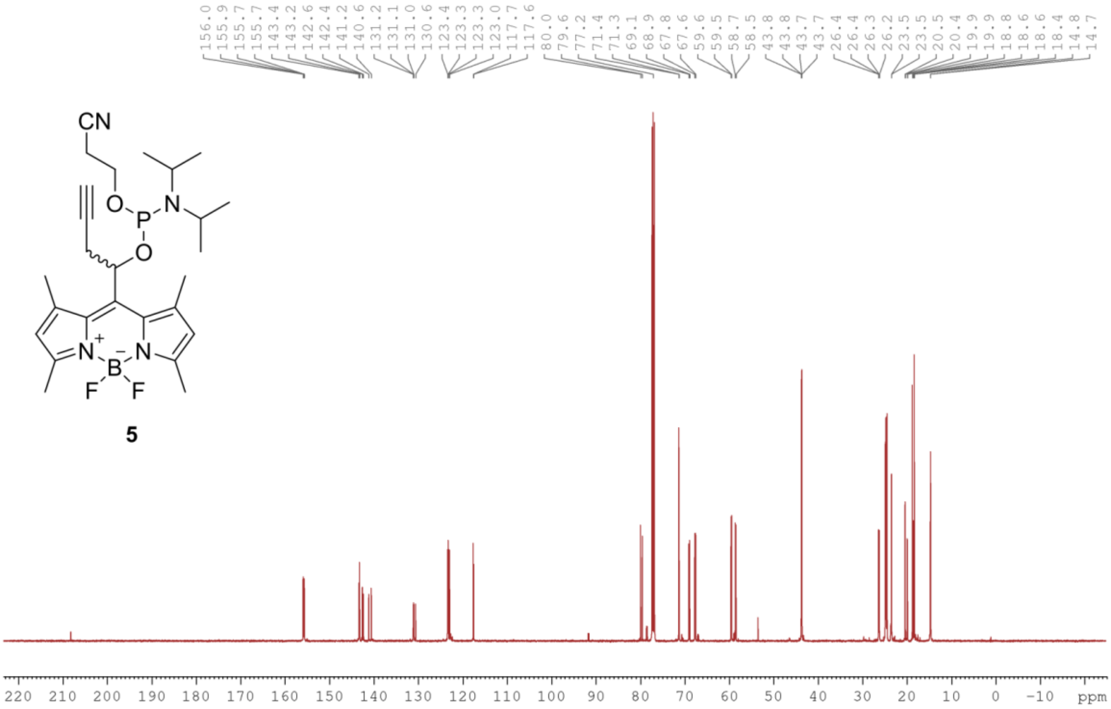

$^{31}P\{^{1}H\}$ *(CDCl$_3$):*

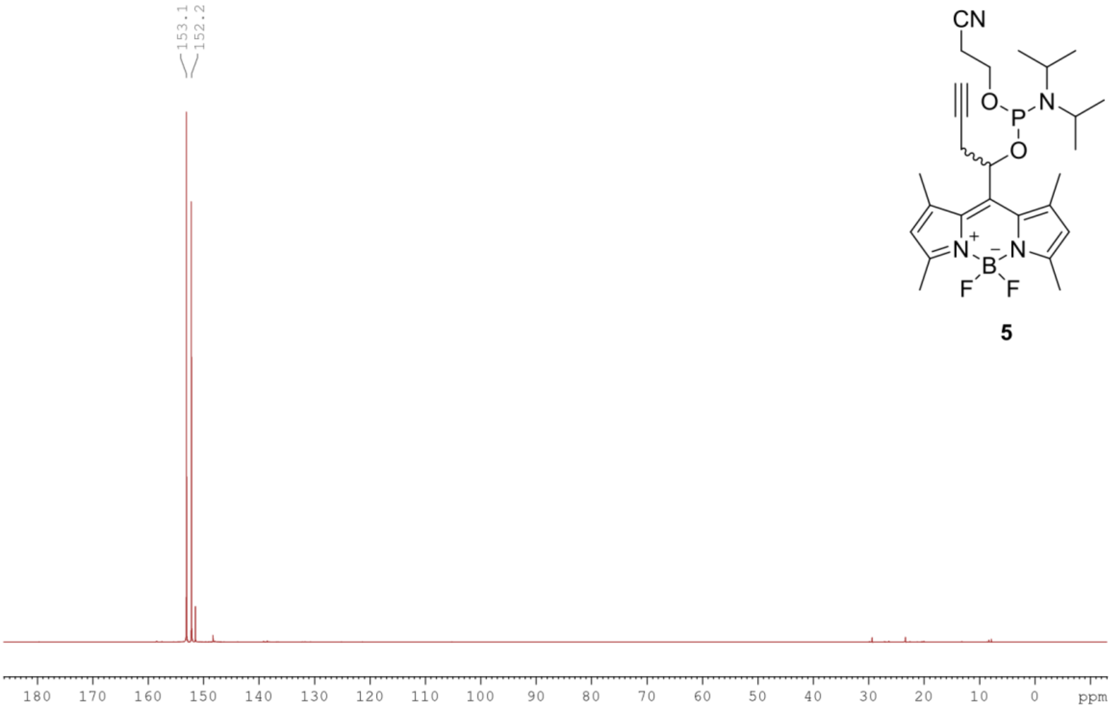

6.2 Coumarin NMR Spectra

(*E*)-7-(Diethylamino)-4-[2-(dimethylamino)vinyl]-*2H*-chromen-2-one (7)

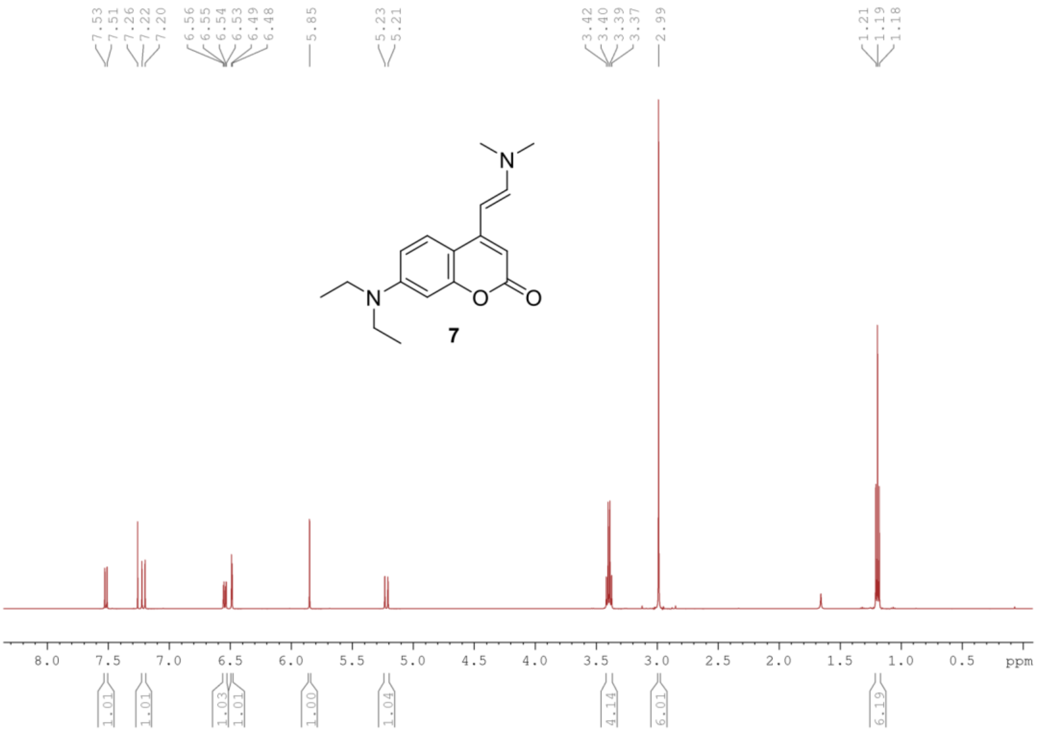

7-(Diethylamino)-2-oxo-*2H*-chromene-4-carbaldehyde (8)

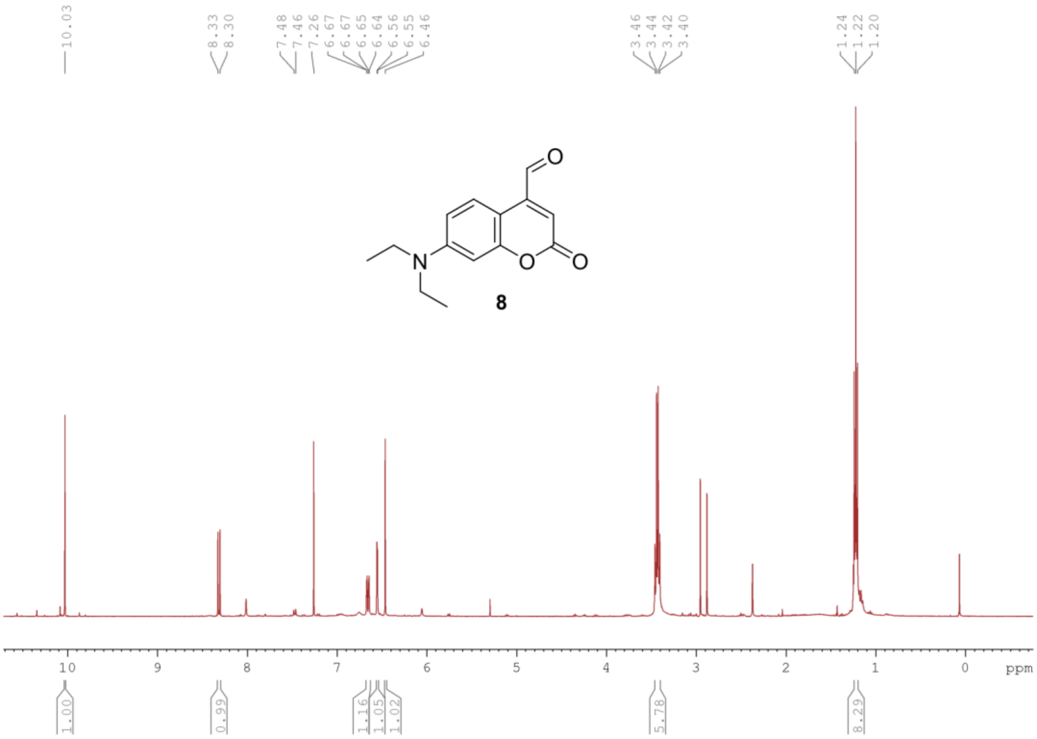

7-(Diethylamino)-4-(1-hydroxybut-3-in-1-yl)-*2H*-chromen-2-one (9)

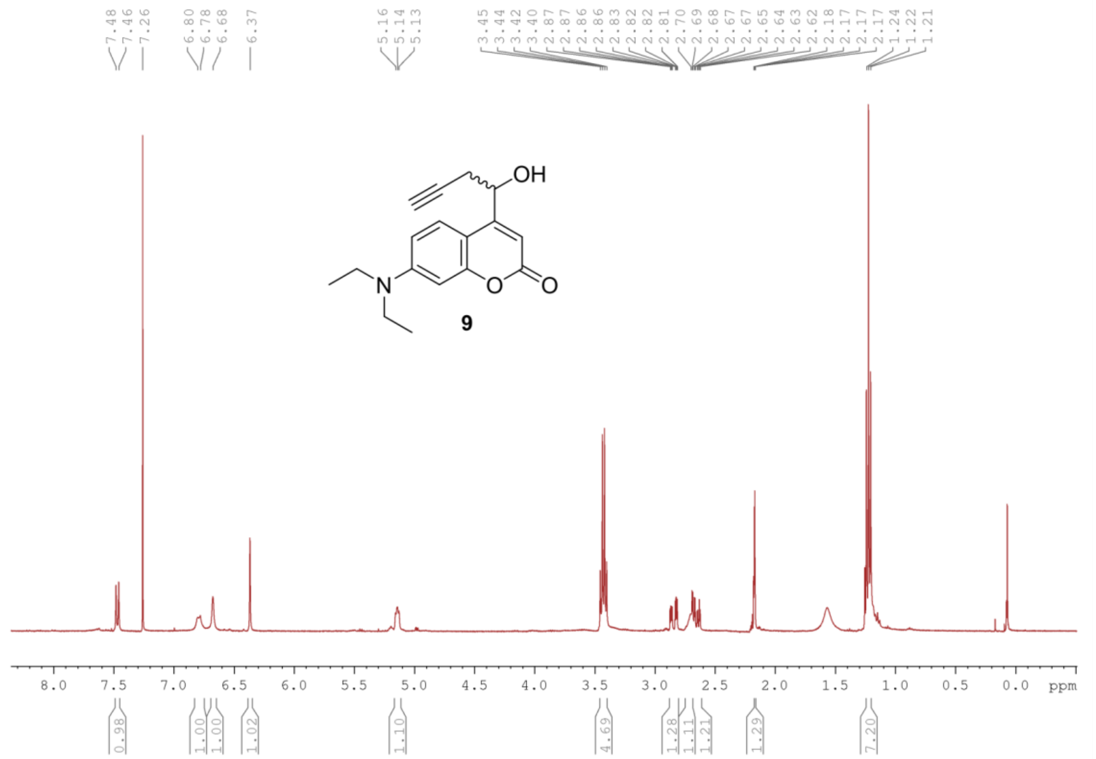

4-{1-[(*tert*-Butyldimethylsilyl)oxy]but-3-yn-1-yl}-7-(diethylamino)-*2H*-chromen-2-one (10)

^{1}H ($CDCl_3$):

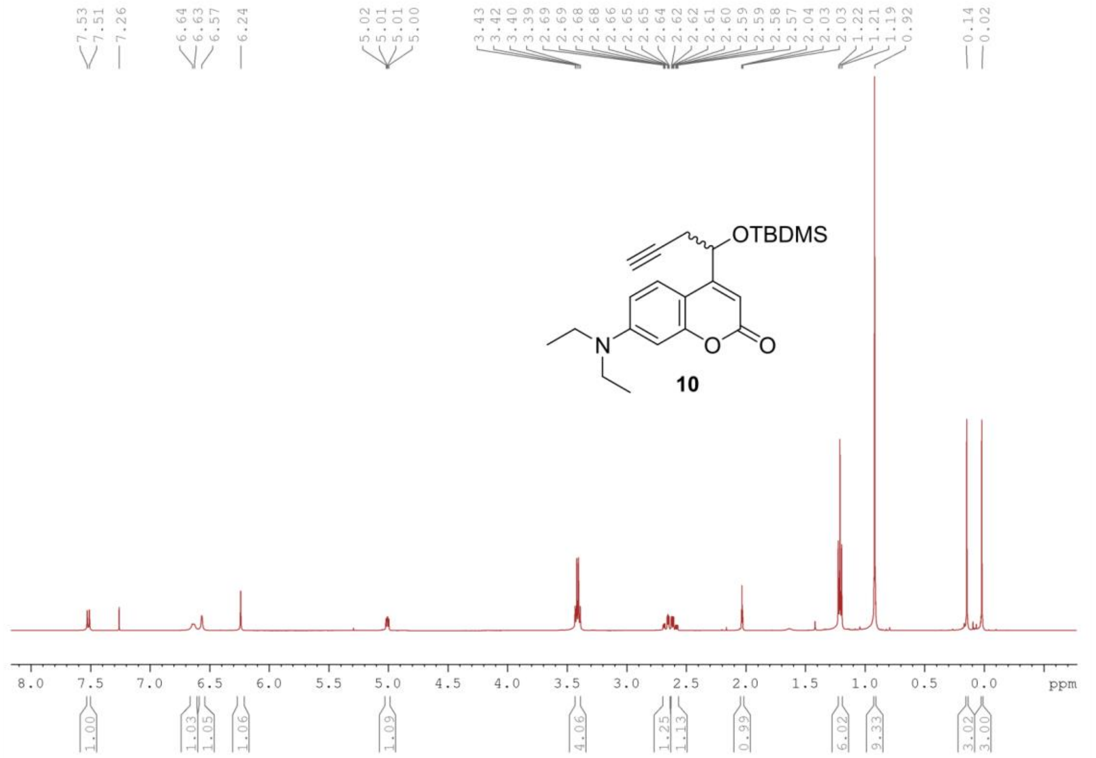

[13]C{[1]H} (CDCl$_3$):

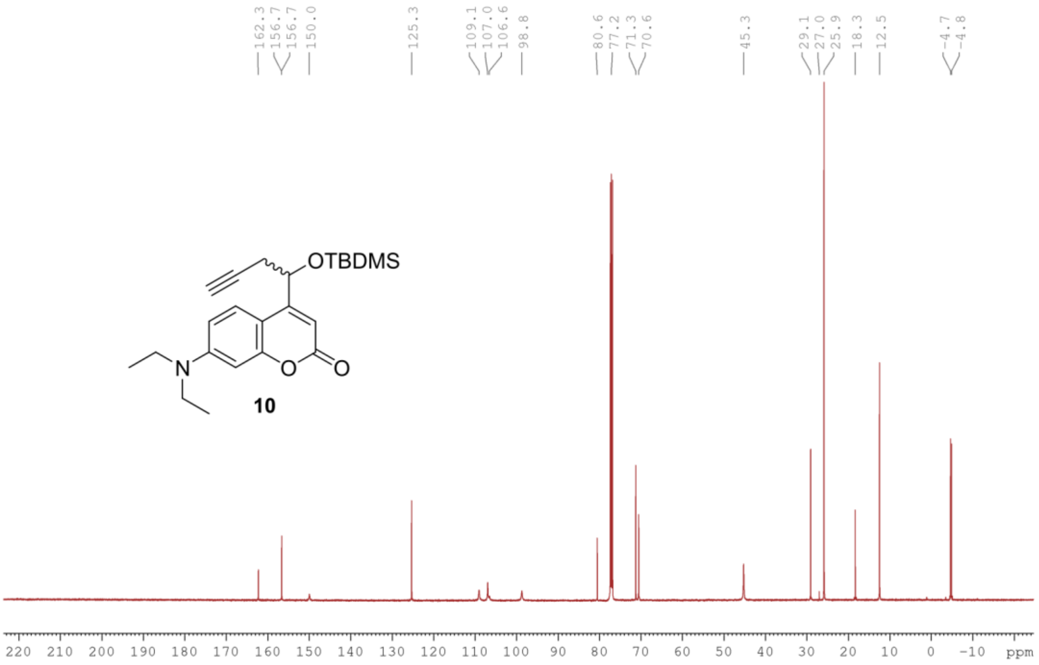

4-{1-[(*tert*-Butyldimethylsilyl)oxy]but-3-yn-1-yl}-7-(diethylamino)-*2H*-chromene-2-thione (11)

[1]H (CDCl$_3$):

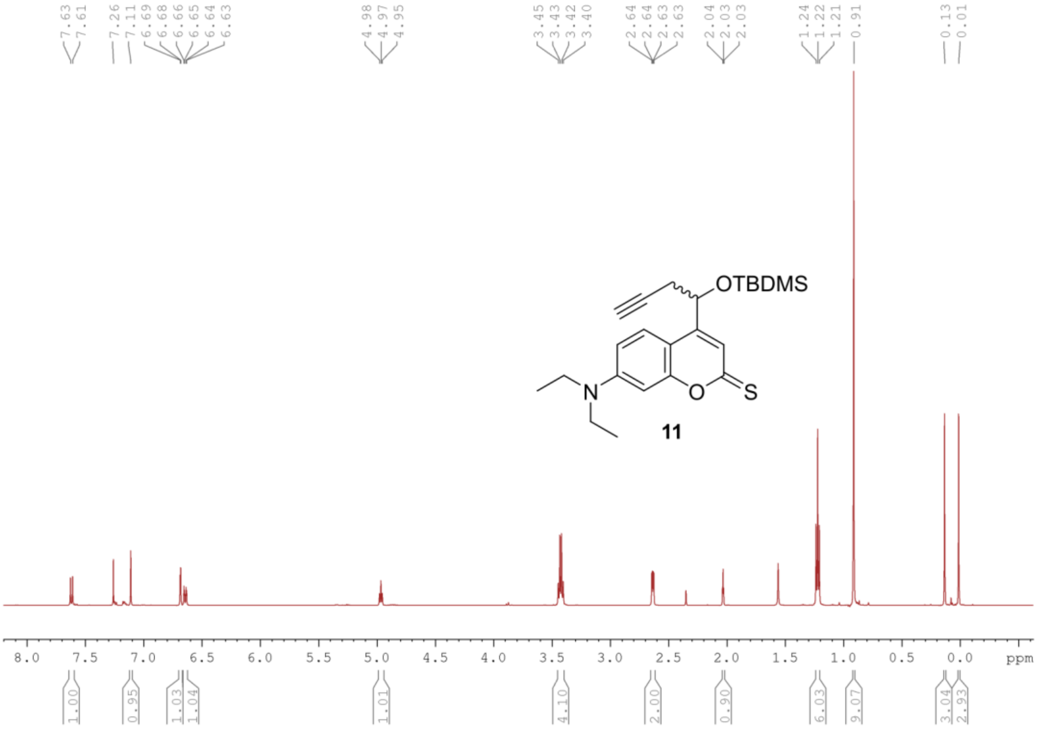

$^{13}C\{^{1}H\}$ (CDCl$_3$):

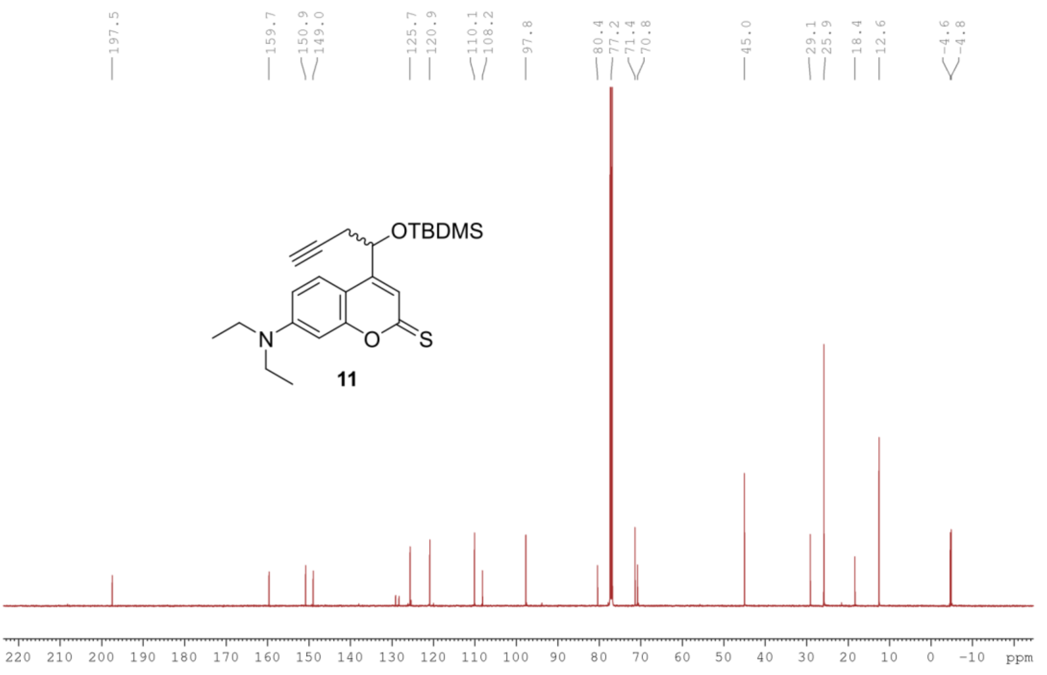

2-(4-{1-[(*tert*-Butyldimethylsilyl)oxy]but-3-yn-1-yl}-7-(diethylamino)-*2H*-chromen-2-ylidene)malononitrile (12)

^{1}H (CDCl$_3$):

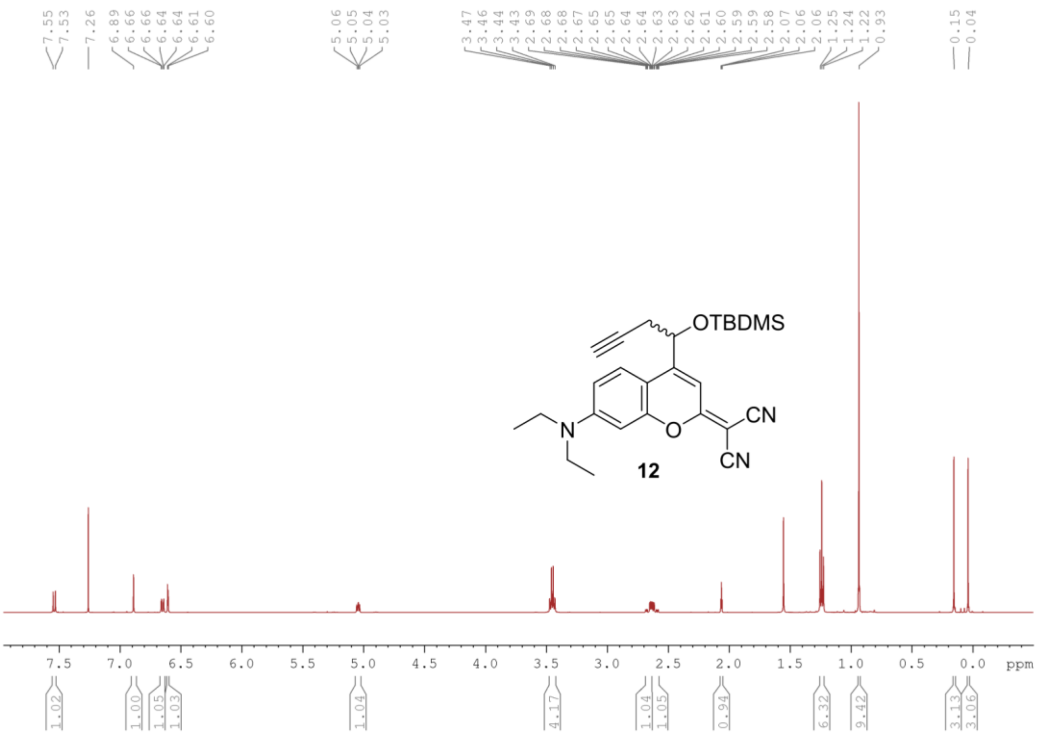

$^{13}C\{^{1}H\}$ *(CDCl$_3$):*

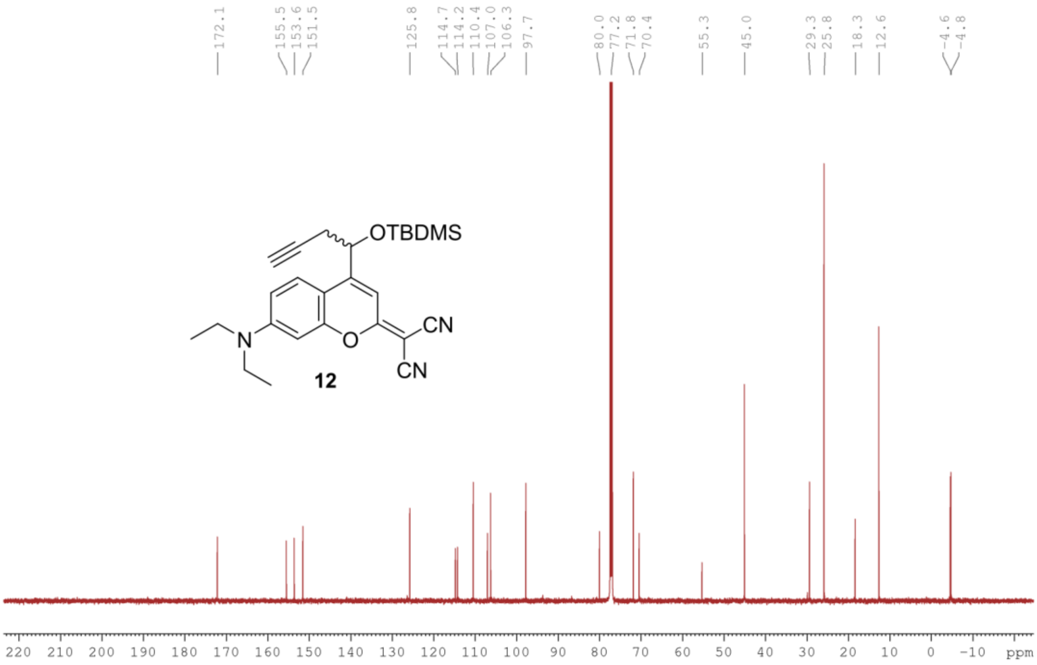

2-[7-(Diethylamino)-4-(1-hydroxybut-3-yn-1-yl)-*2H*-chromen-2-ylidene]malononitrile (13)

^{1}H *(CDCl$_3$):*

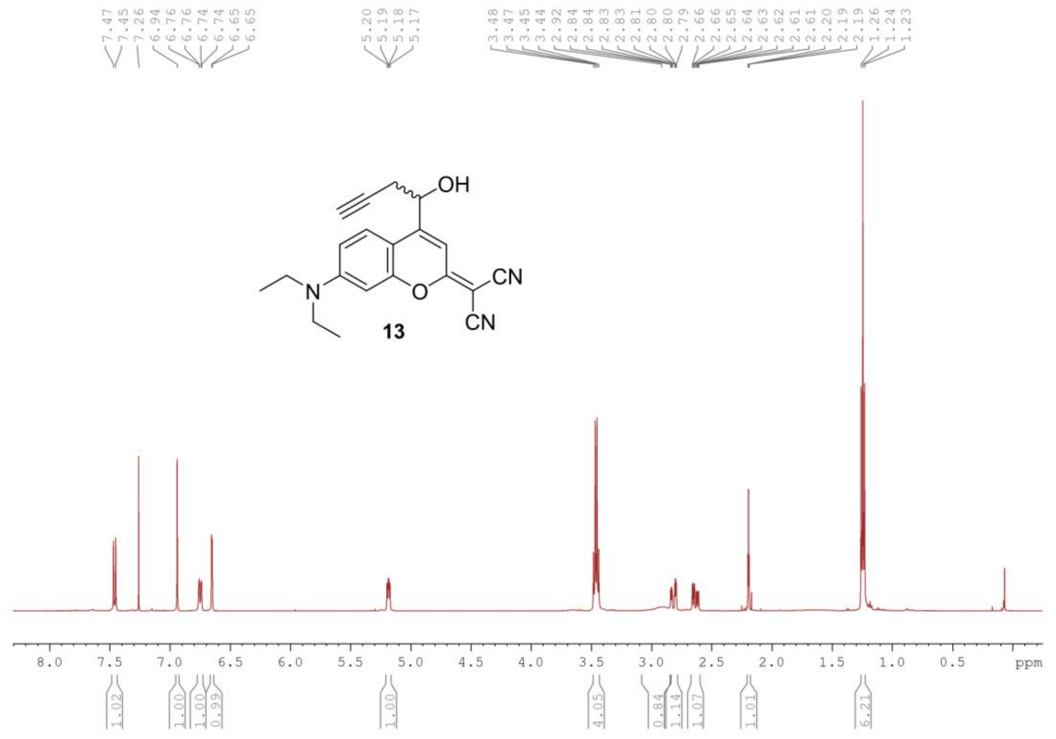

[13]C{[1]H} (CDCl$_3$):

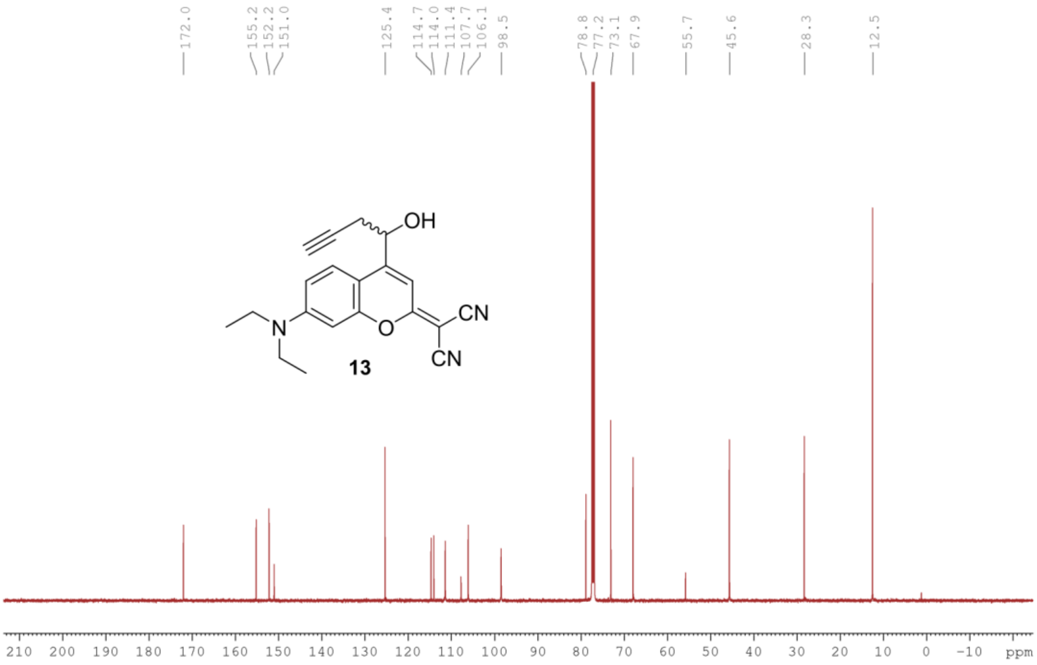

2-Cyanoethyl-{1-[2-(dicyanomethylene)-7-(diethylamino)-*2H*-chromen-4-yl]but-3-yn-1-yl}-*N*,*N*-diisopropylphosphoramidite (14)

[1]H (CDCl$_3$):

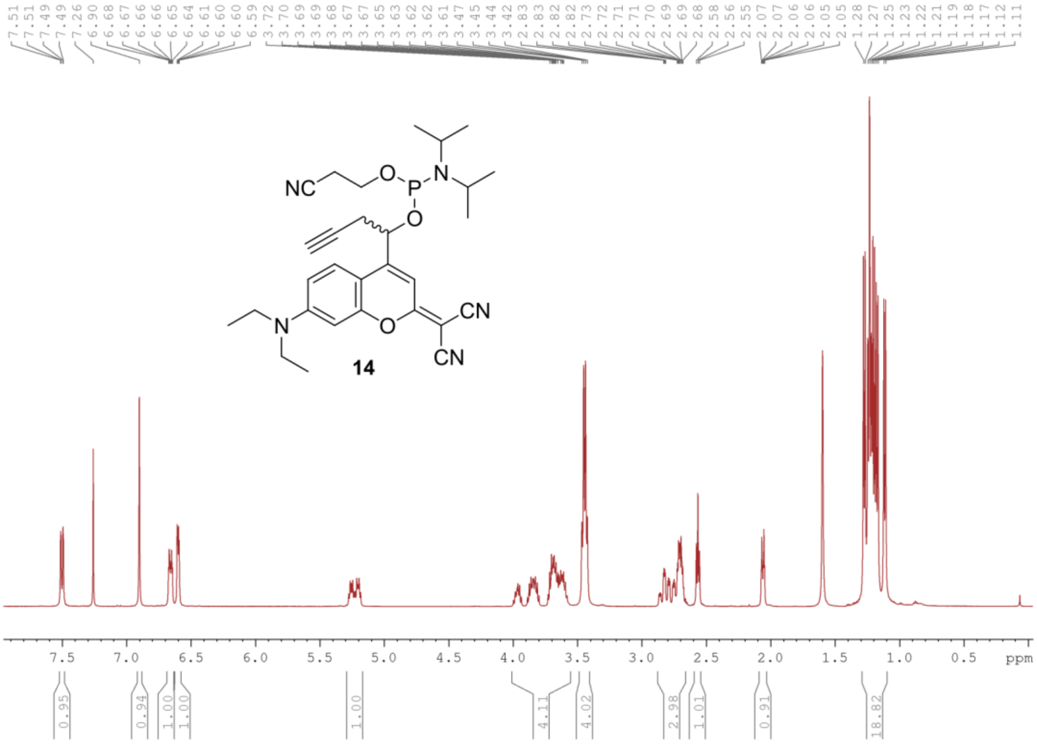

[1]H (DMSO-d6):

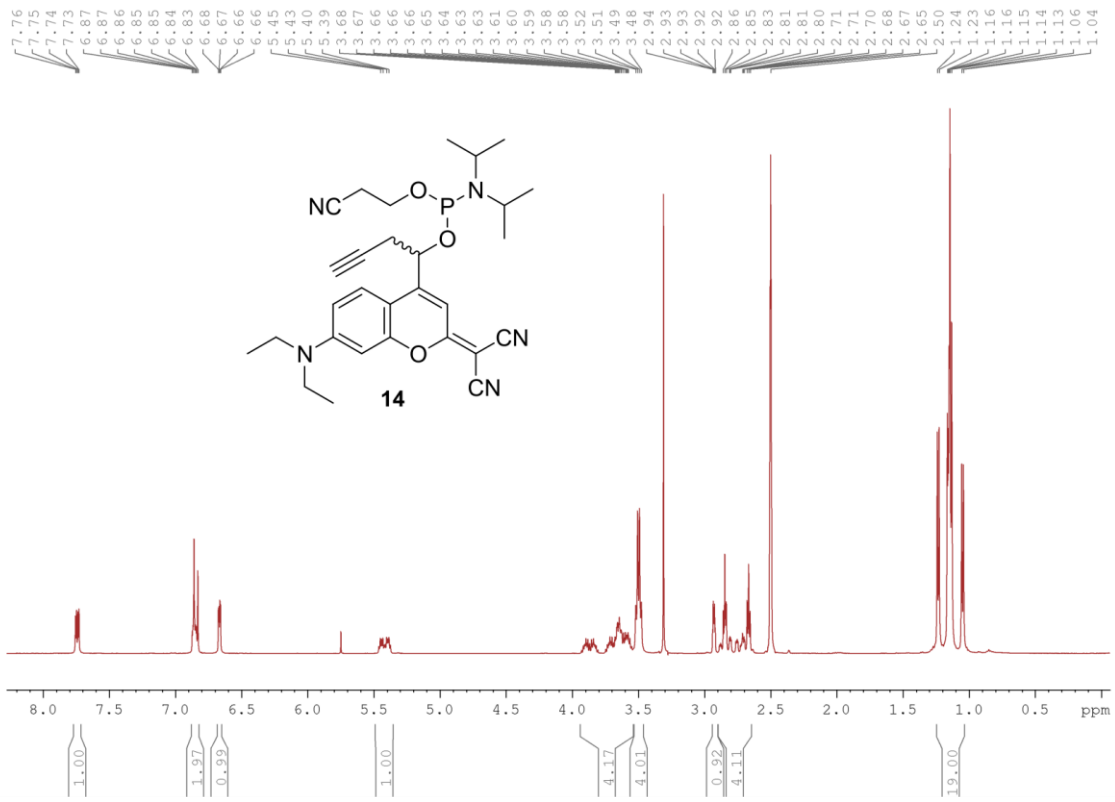

[13]C{[1]H} (CDCl3):

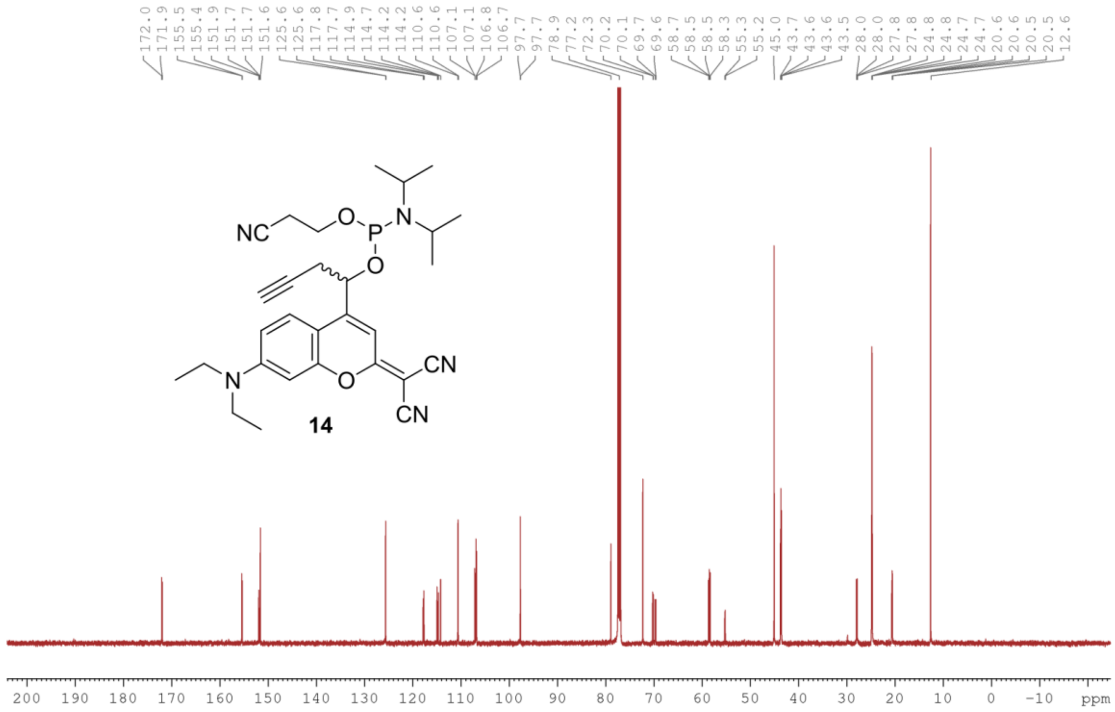

$^{13}C\{^1H\}$ (DMSO-d_6):

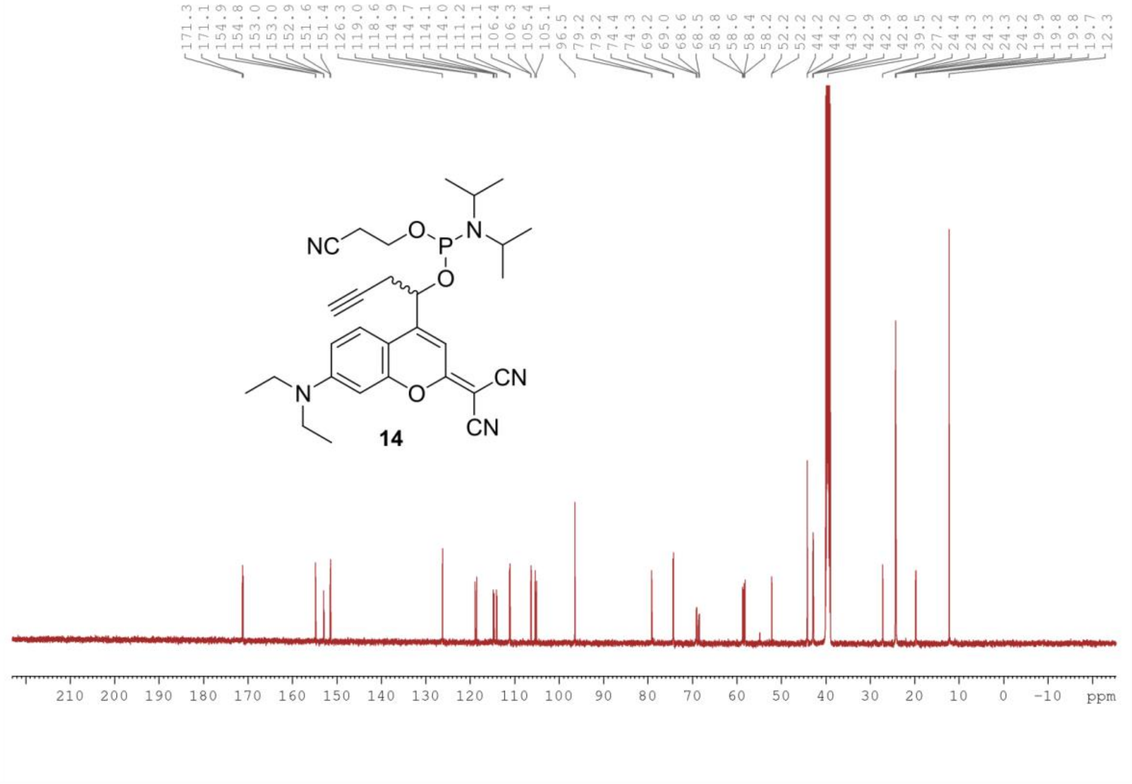

$^{31}P\{^1H\}$ ($CDCl_3$):

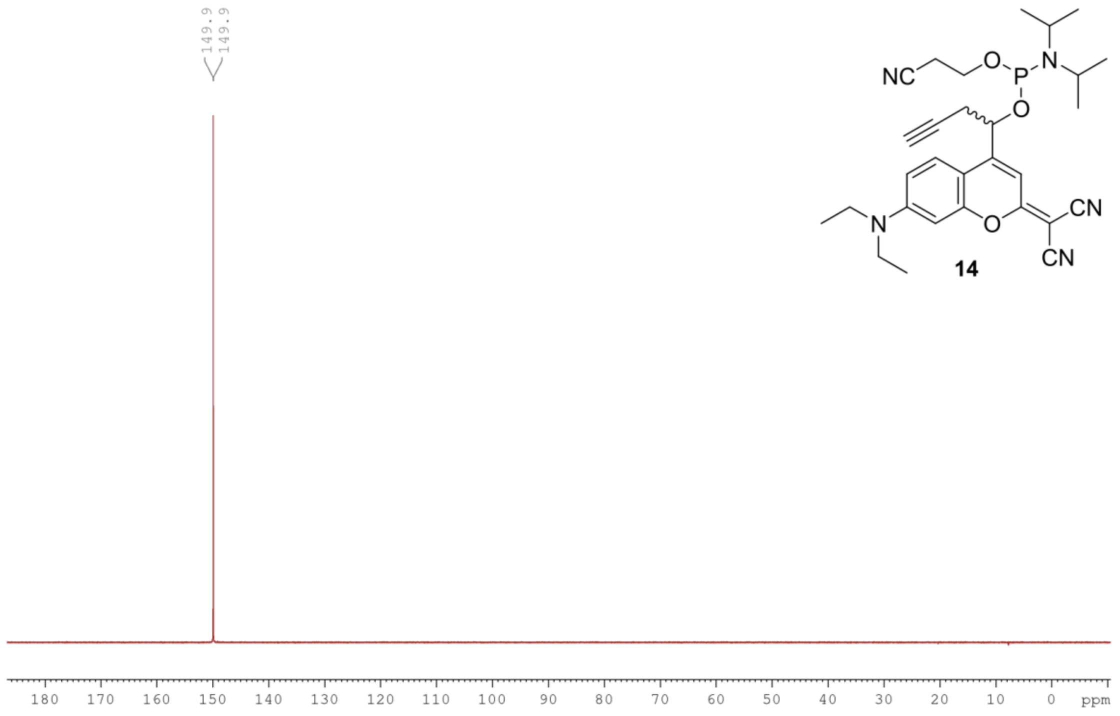

$^{31}P\{^{1}H\}$ (DMSO-d$_6$):

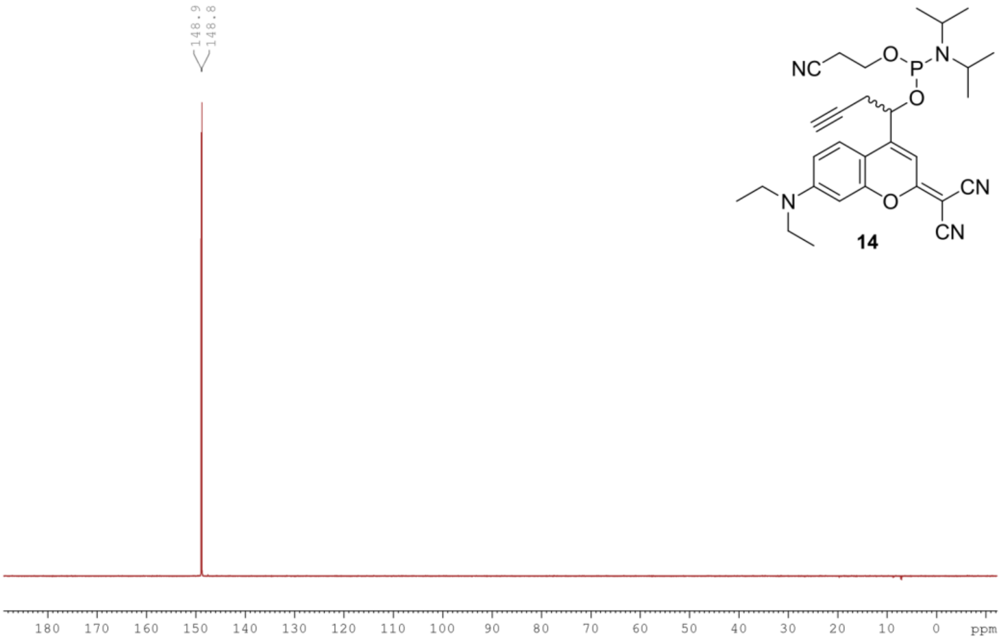

7 Mass Spectra

7.1 BODIPY Mass Spectra

8-Acetoxymethyl-4,4'-difluoro-1,3,5,7-tetramethyl-4-bora-3a,4a-diaza-*s*-indacene (1)

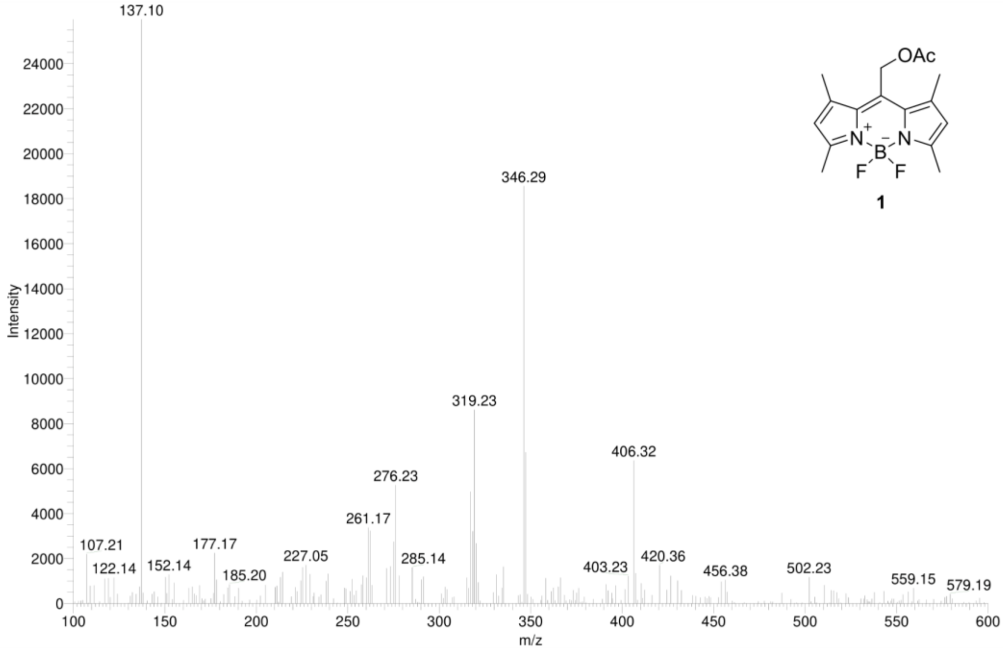

4,4'-Difluoro-8-hydroxymethyl-1,3,5,7-tetramethyl-4-bora-3a,4a-diaza-*s*-indacene (2)

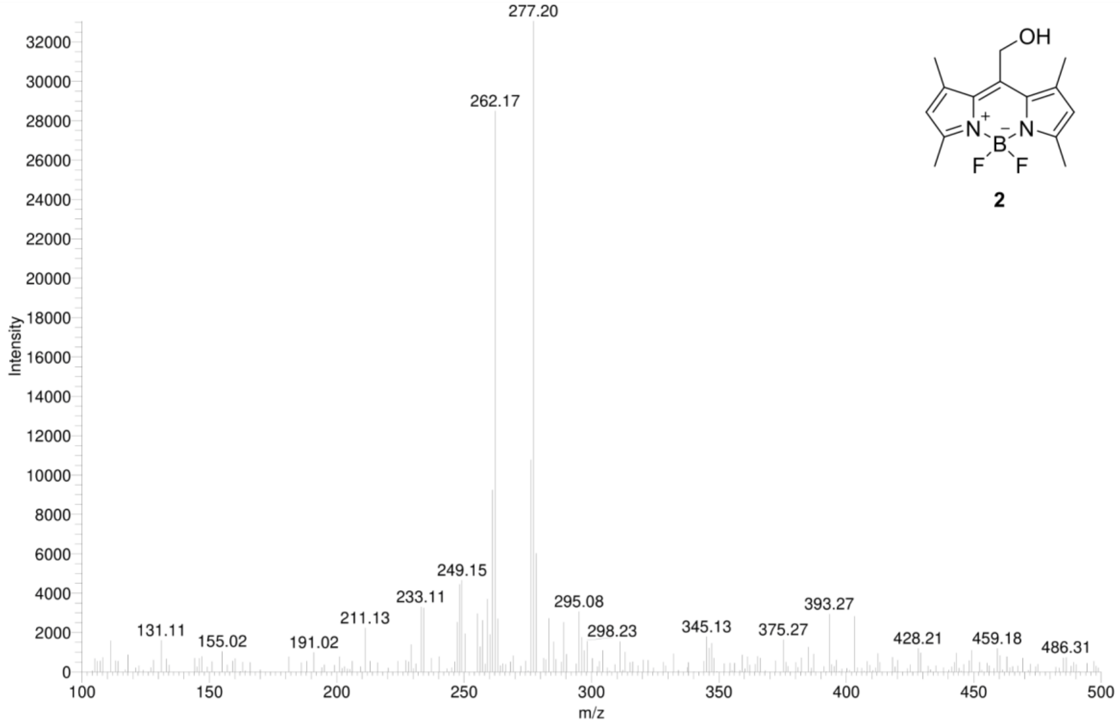

4,4'-Difluoro-8-(1-hydroxybut-3-in-1-yl)-1,3,5,7-tetramethyl-4-bora-3a,4a-diaza-*s*-indacene (4)

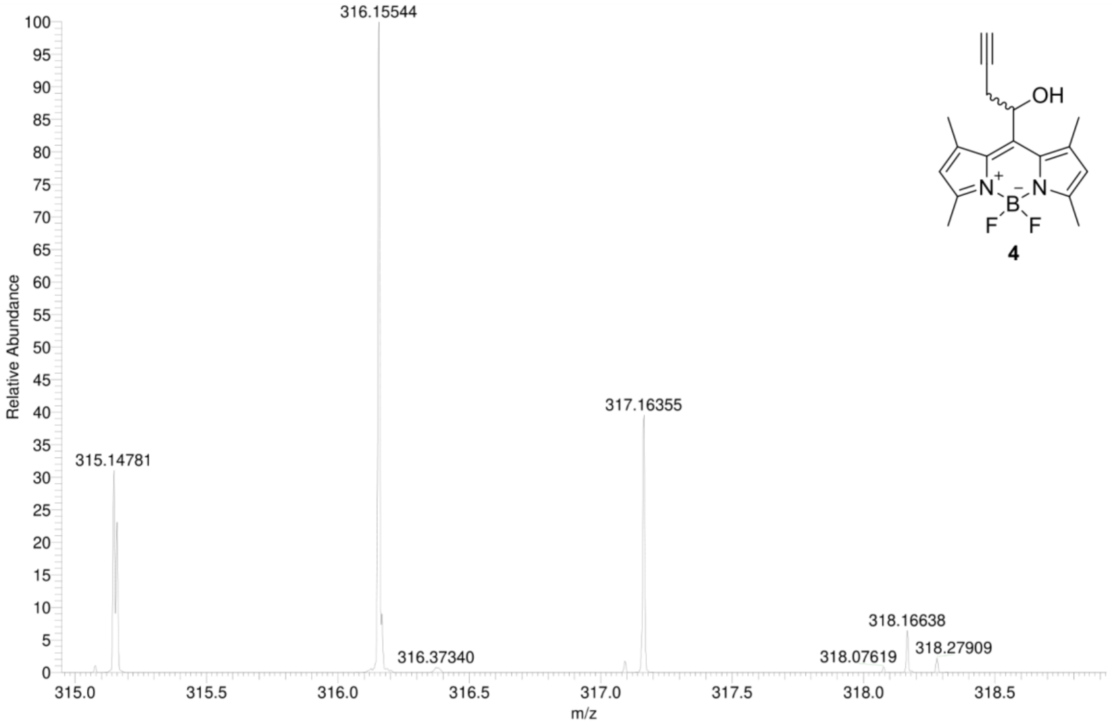

2-Cyanoethyl-[1-(4,4’-difluoro-1,3,5,7-tetramethyl-4-bora-3a,4a-diaza-*s*-indacene-8-yl)but-3-yn-1-yl]-*N*,*N*-diisopropylphosphoramidite (5)

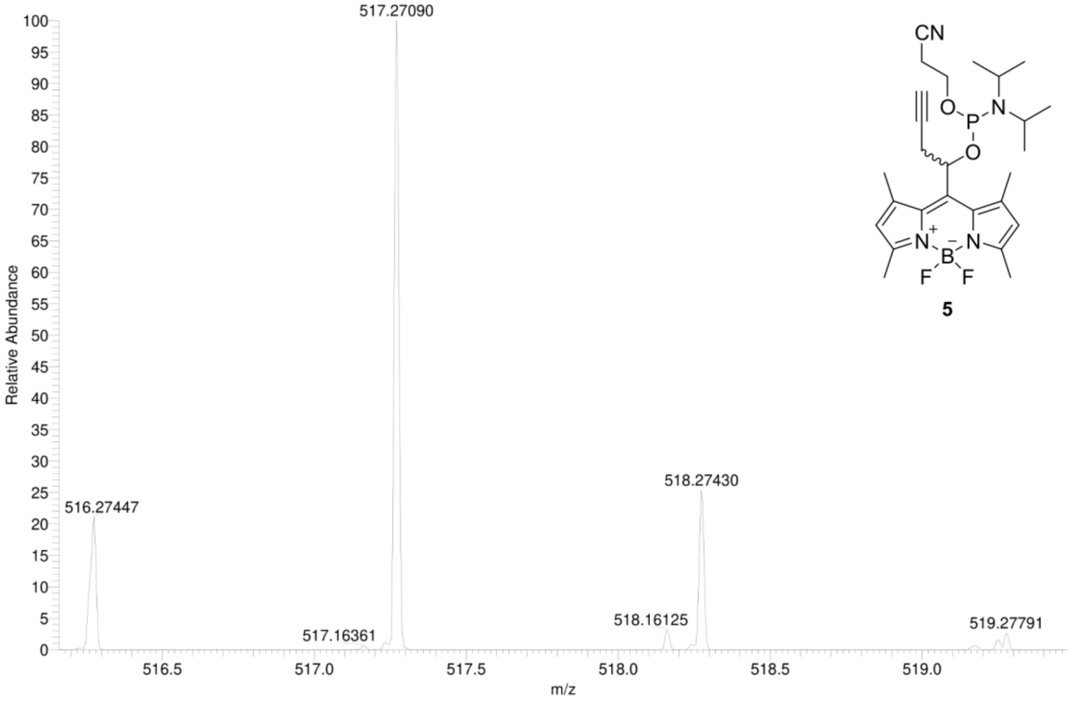

7.2 Coumarin Mass Spectra

(*E*)-7-(Diethylamino)-4-[2-(dimethylamino)vinyl]-*2H*-chromen-2-one (7)

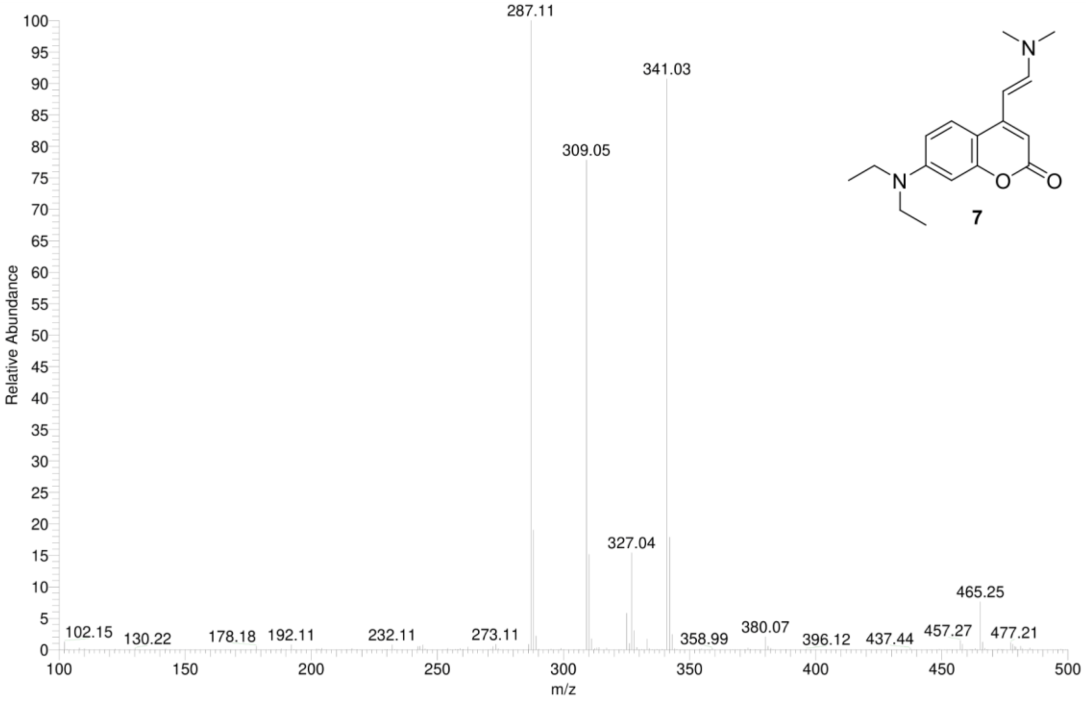

7-(Diethylamino)-2-oxo-*2H*-chromene-4-carbaldehyde (8)

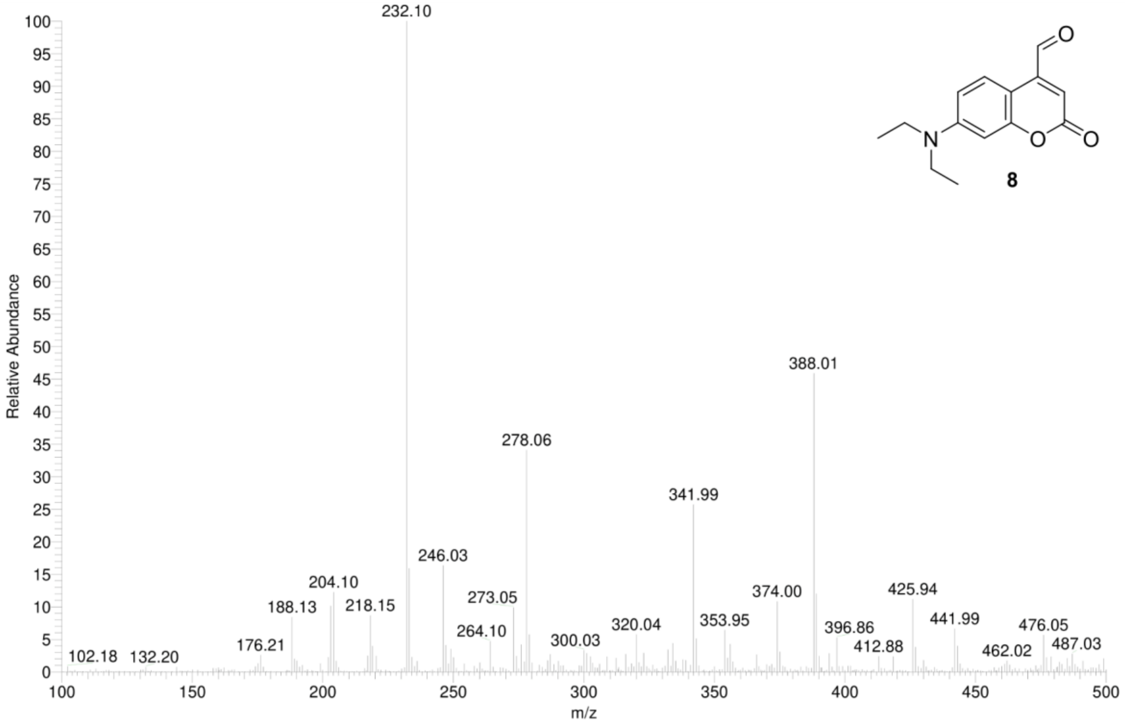

7-(Diethylamino)-4-(1-hydroxybut-3-in-1-yl)-*2H*-chromen-2-one (9)

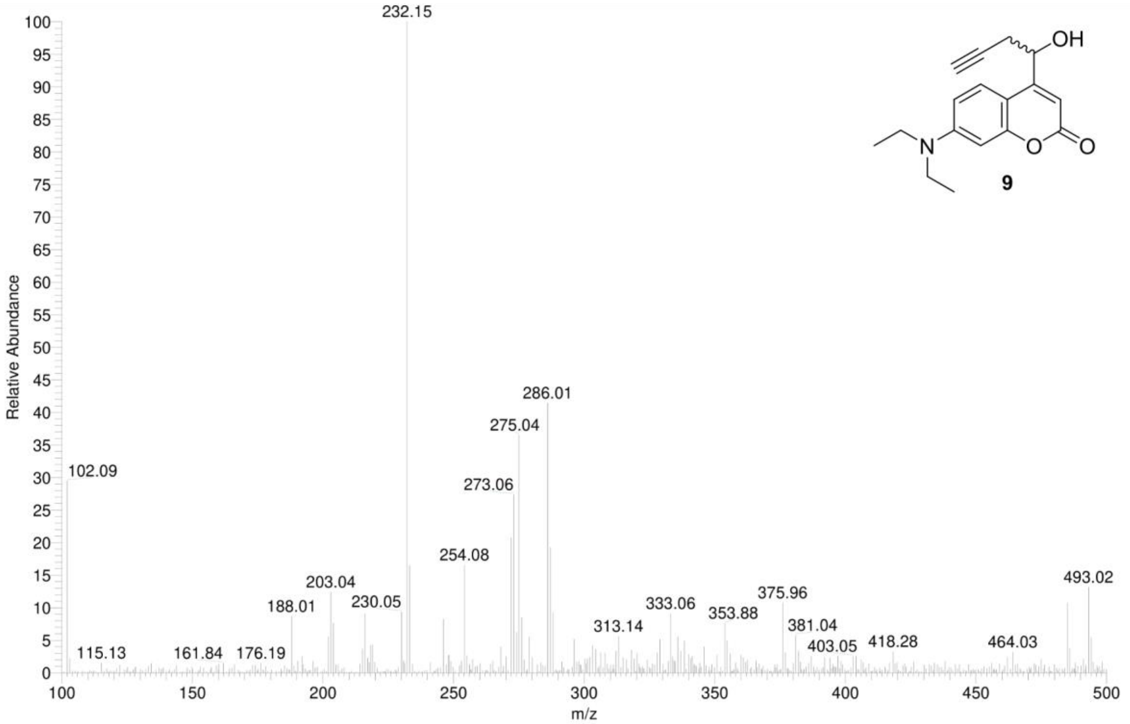

4-{1-[(*tert*-Butyldimethylsilyl)oxy]but-3-yn-1-yl}-7-(diethylamino)-*2H*-chromen-2-one (10)

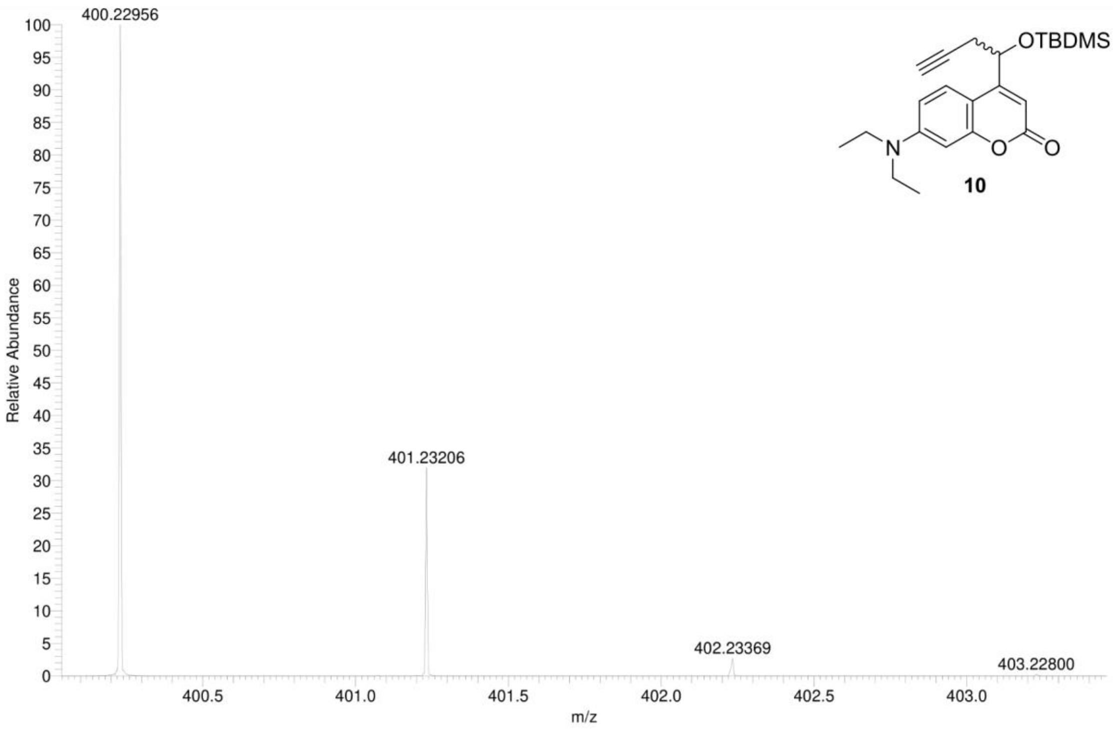

4-{1-[(*tert*-Butyldimethylsilyl)oxy]but-3-yn-1-yl}-7-(diethylamino)-*2H*-chromene-2-thione (11)

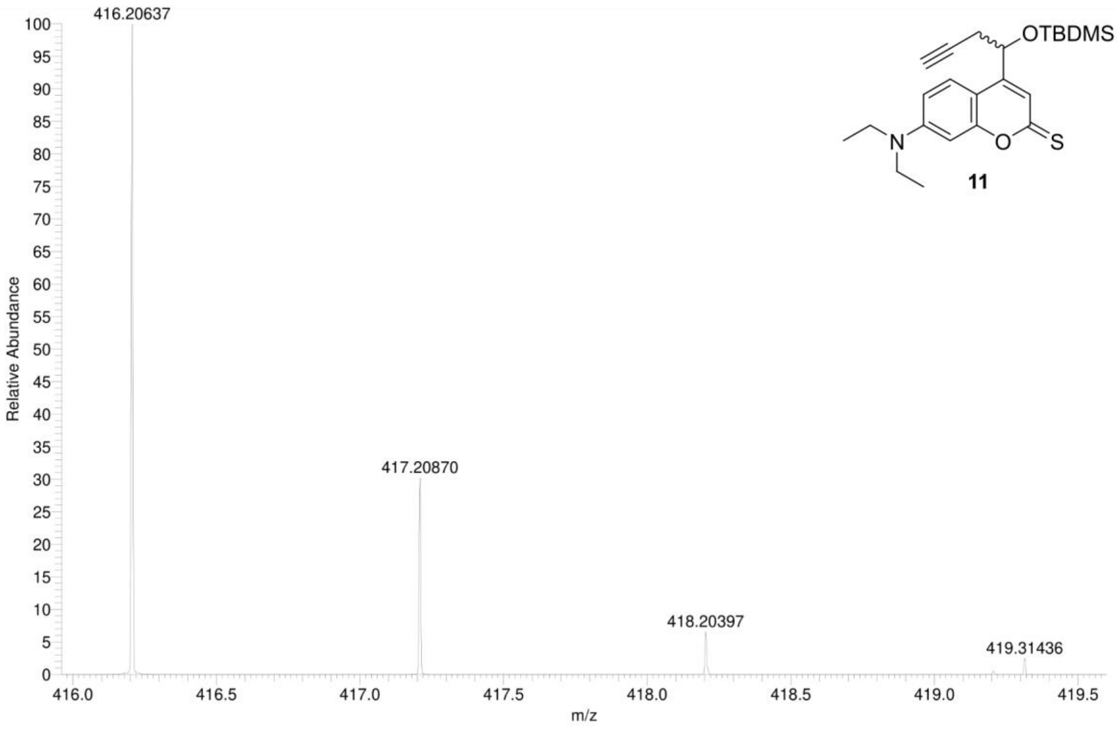

2-(4-{1-[(*tert*-Butyldimethylsilyl)oxy]but-3-yn-1-yl}-7-(diethylamino)-*2H*-chromen-2-ylidene)malononitrile (12)

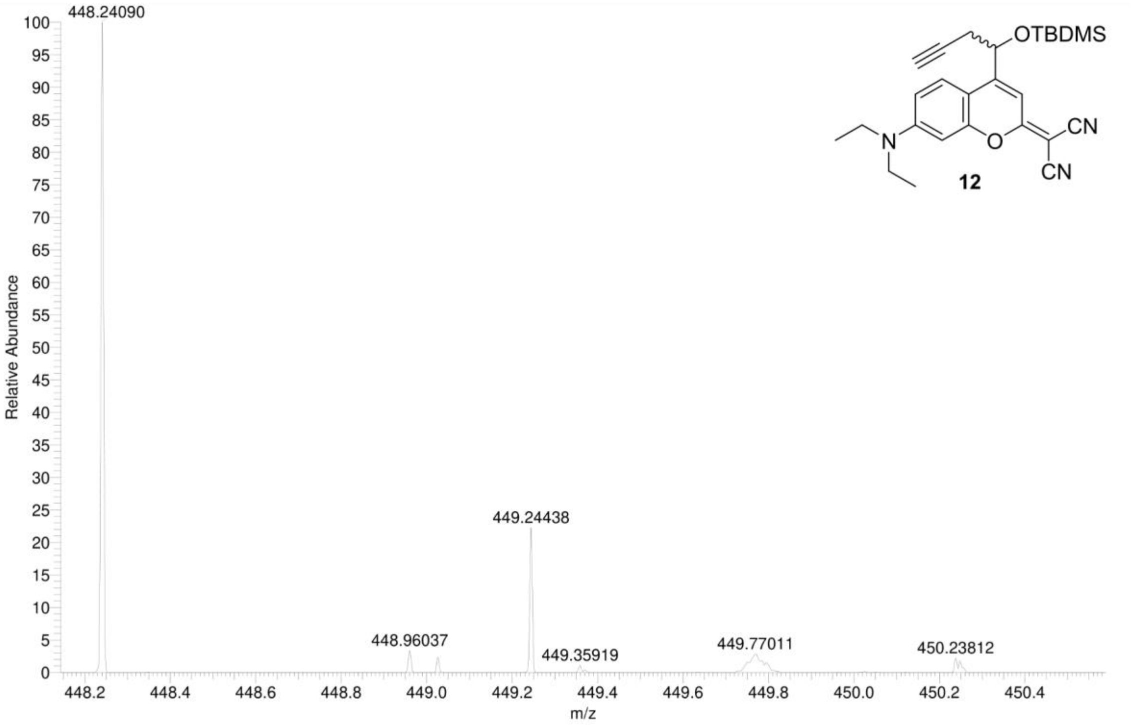

2-[7-(Diethylamino)-4-(1-hydroxybut-3-yn-1-yl)-*2H*-chromen-2-ylidene]malononitrile (13)

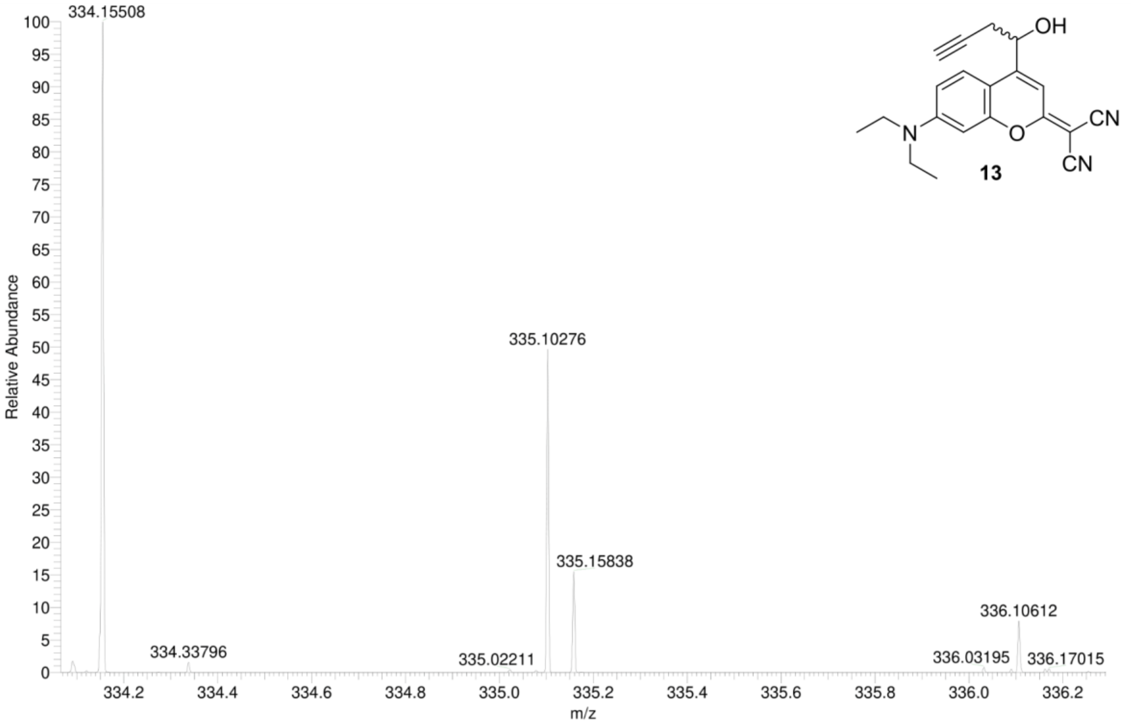

2-Cyanoethyl-{1-[2-(dicyanomethylene)-7-(diethylamino)-*2H*-chromen-4-yl]but-3-yn-1-yl}-*N*,*N*-diisopropylphosphoramidite (14)

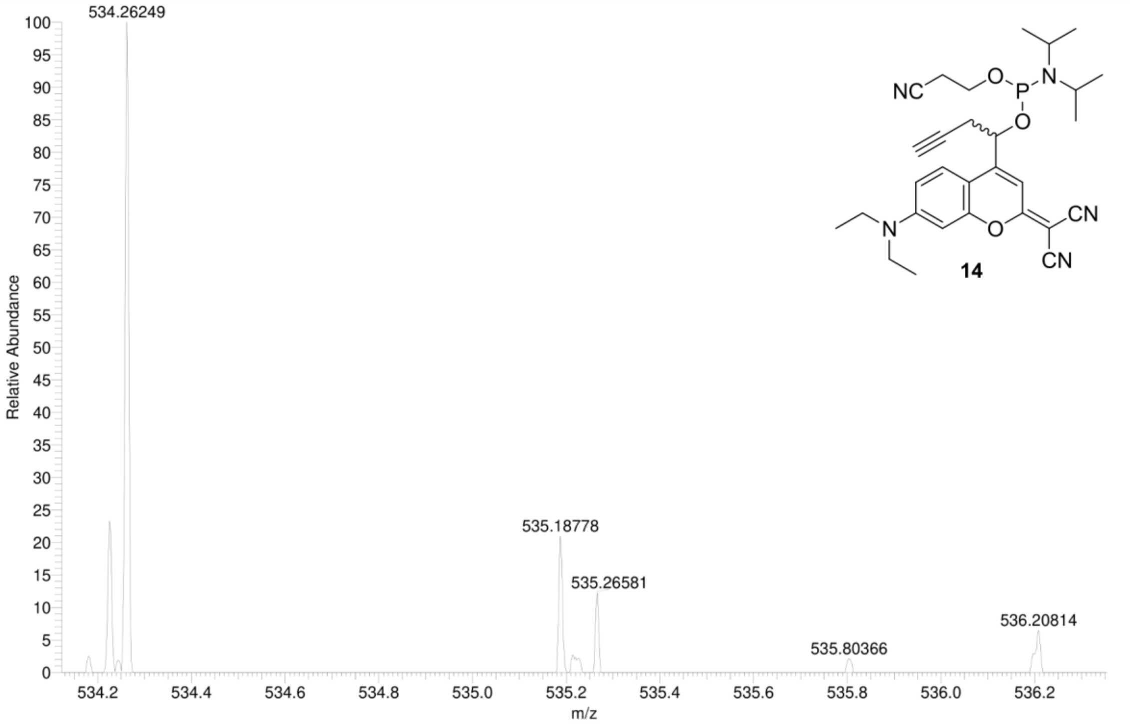

7.3 Oligonucleotide Mass Spectra

Table S10: Calculated and found masses for all synthesized oligonucleotides.

	Calculated Mass	Found Mass
**ON1**	3082.6189 Da	3081.9794 Da
**cON1**	3270.6369 Da	3271.0986 Da
**ON2**	3038.5944 Da	3039.0058 Da
**cON2**	3226.6124 Da	3227.1316 Da
**ON3**	2765.5167 Da	2765.8663 Da
**ON4**	5075.8588 Da	5076.5264 Da
**ON4** (AMB-conjugated)	5263.8768 Da	5264.6145 Da
**ON5**	8029.3035 Da	8030.4065 Da

ON1:

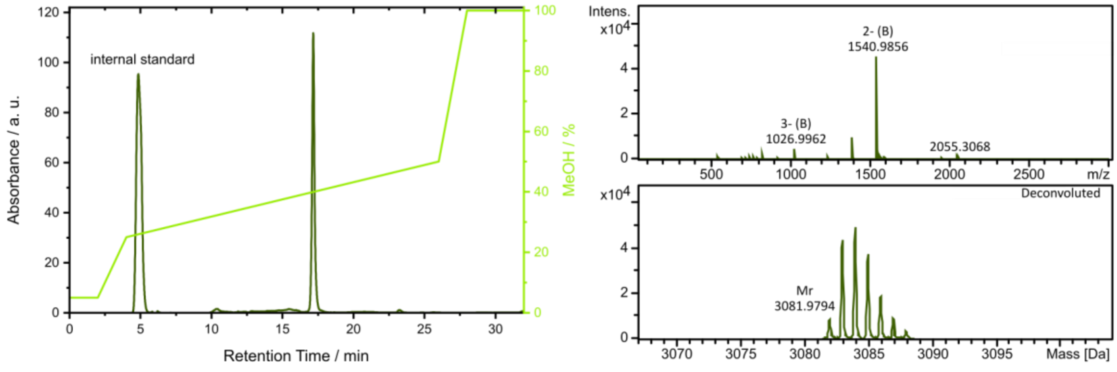

internal standard
Absorbance / a. u.
Retention Time / min
MeOH / %
Intens.
2- (B)
1540.9856
3- (B)
1026.9962
2055.3068
m/z
Deconvoluted
Mr
3081.9794
Mass [Da]

cON1:

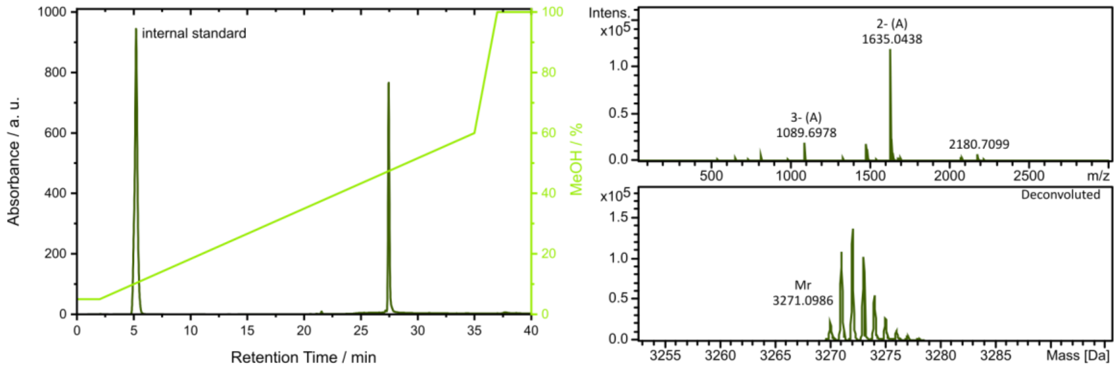

internal standard
Absorbance / a. u.
Retention Time / min
MeOH / %
Intens.
2- (A)
1635.0438
3- (A)
1089.6978
2180.7099
m/z
Deconvoluted
Mr
3271.0986
Mass [Da]

ON2:

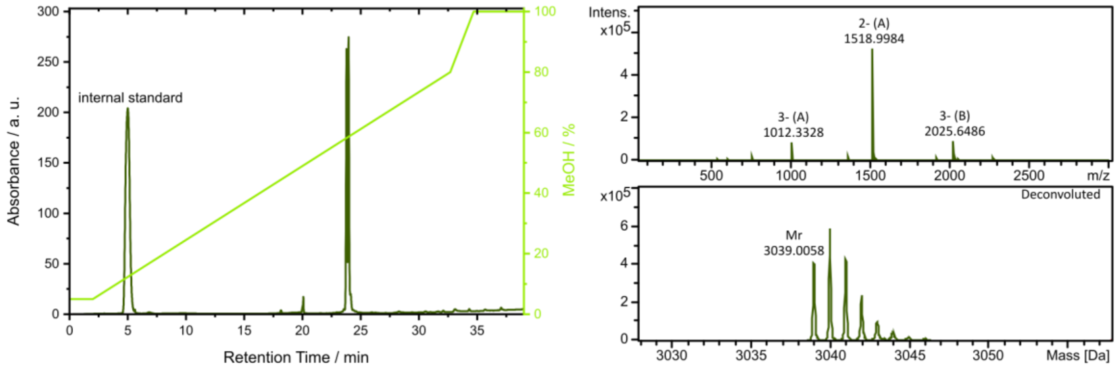

internal standard
Absorbance / a. u.
Retention Time / min
MeOH / %
Intens.
2- (A)
1518.9984
3- (A)
1012.3328
3- (B)
2025.6486
m/z
Deconvoluted
Mr
3039.0058
Mass [Da]

cON2:

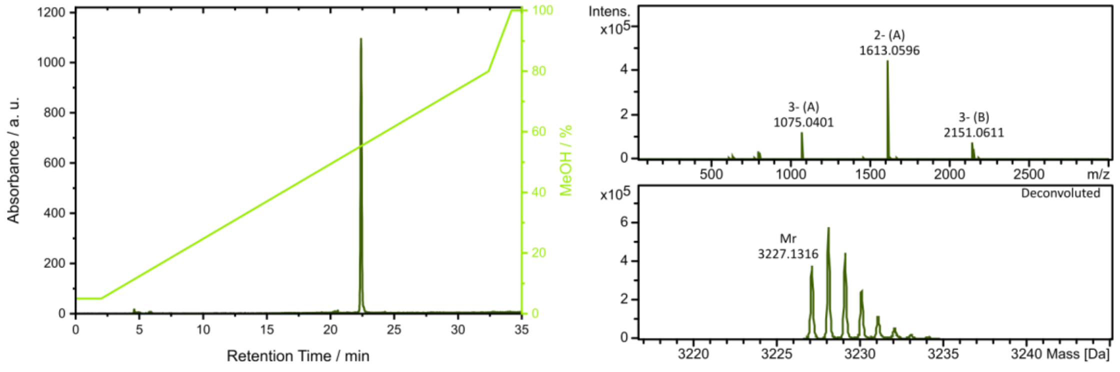
Absorbance / a. u.
Retention Time / min
MeOH / %
Intens. x10^5
2- (A) 1613.0596
3- (A) 1075.0401
3- (B) 2151.0611
m/z
Deconvoluted
Mr 3227.1316
Mass [Da]

ON3:

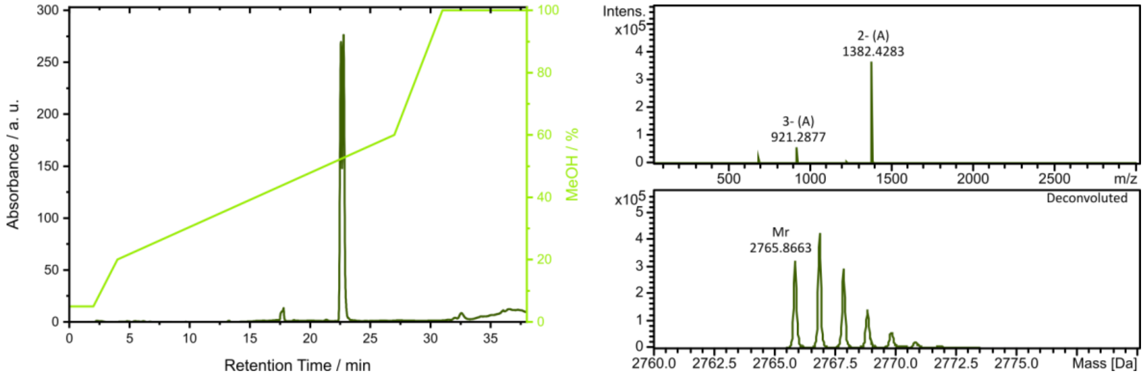
Absorbance / a. u.
Retention Time / min
MeOH / %
Intens. x10^5
2- (A) 1382.4283
3- (A) 921.2877
m/z
Deconvoluted
Mr 2765.8663
Mass [Da]

ON4:

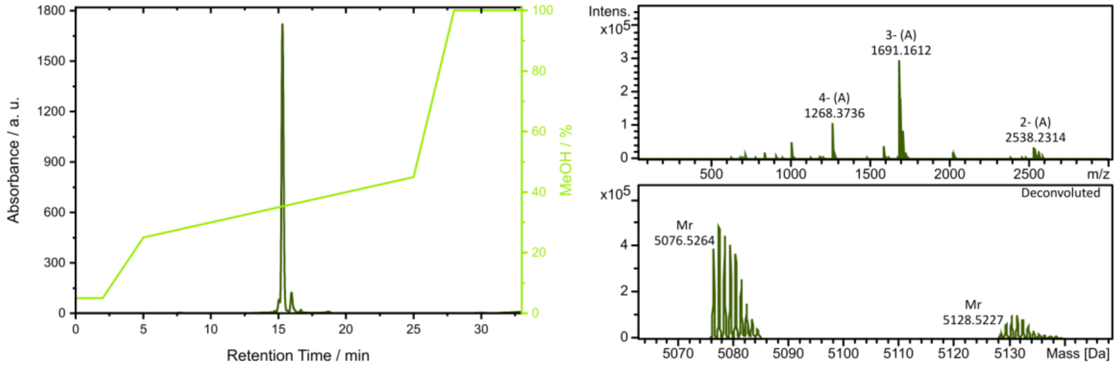
Absorbance / a. u.
Retention Time / min
MeOH / %
Intens. x10^5
3- (A) 1691.1612
4- (A) 1268.3736
2- (A) 2538.2314
m/z
Deconvoluted
Mr 5076.5264
Mr 5128.5227
Mass [Da]

ON4 (AMB-conjugated):

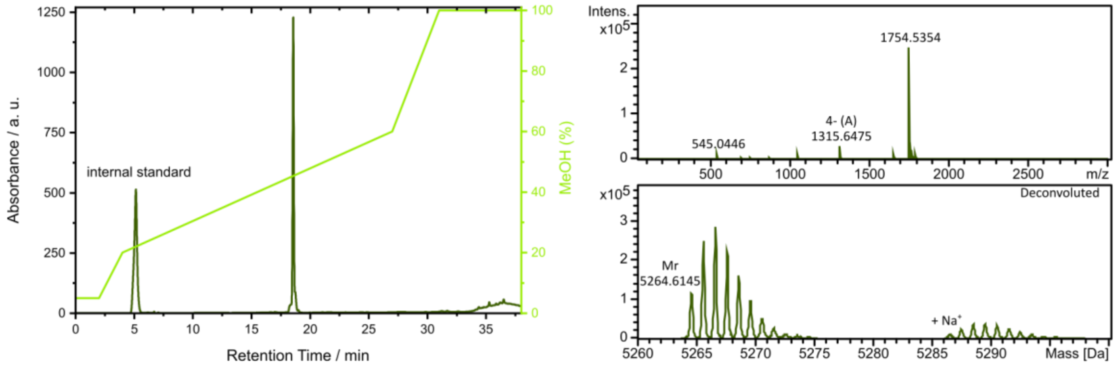

ON5:

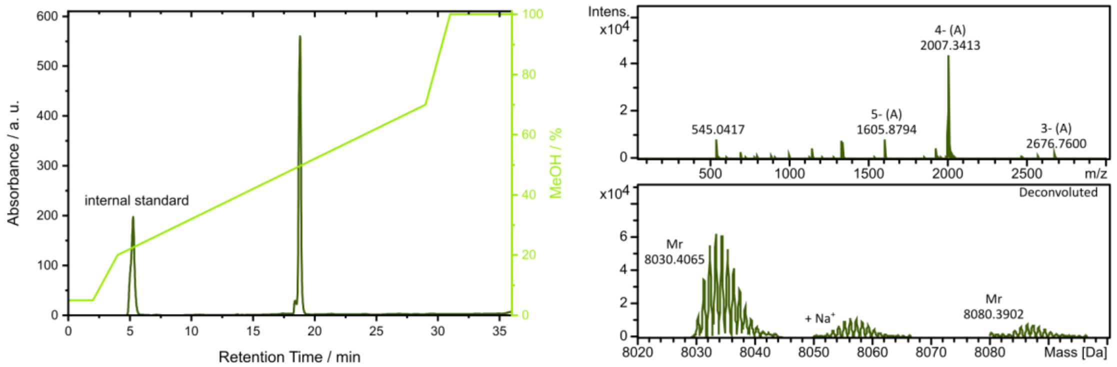

8 References

[1] K. Krumova, G. Cosa, *J. Am. Chem. Soc.* **2010**, *132*, 17560–17569.

[2] T. Weinrich, M. Gränz, C. Grünewald, T. F. Prisner, M. W. Göbel, *Eur. J. Org. Chem.* **2017**, *2017*, 491–496.

[3] P. Seyfried, L. Eiden, N. Grebenovsky, G. Mayer, A. Heckel, *Angew. Chem. Int. Ed.* **2017**, *56*, 359–363.

[4] M. Reinfelds, V. Hermanns, T. Halbritter, J. Wachtveitl, M. Braun, T. Slanina, A. Heckel, *ChemPhotoChem* **2019**, *3*, 441–449.
